# A phase I study of talazoparib (BMN 673) combined with carboplatin and paclitaxel in patients with advanced solid tumors (NCI9782)

**DOI:** 10.1002/cam4.4724

**Published:** 2022-04-08

**Authors:** Ticiana A. Leal, Marina N. Sharifi, Nancy Chan, Robert Wesolowski, Anita A. Turk, Justine Y. Bruce, Ruth M. O'Regan, Jens Eickhoff, Lisa M. Barroilhet, Jyoti Malhotra, Janice Mehnert, Eugenia Girda, Elizabeth Wiley, Natalie Schmitz, Shannon Andrews, Glenn Liu, Kari B. Wisinski

**Affiliations:** ^1^ Winship Cancer Institute Emory University Georgia; ^2^ University of Wisconsin Carbone Cancer Center Madison Wisconsin USA; ^3^ Rutgers Cancer Institute of New Jersey New Brunswick New Jersey USA; ^4^ Ohio State University Comprehensive Cancer Center Columbus Ohio USA; ^5^ Indiana University Simon Comprehensive Cancer Center Indianapolis Indiana USA; ^6^ Department of Medicine University of Rochester Rochester New York USA; ^7^ Department of Biostatistics University of Wisconsin School of Medicine and Public Health Madison Wisconsin USA; ^8^ Department of Obstetrics and Gynecology University of Wisconsin School of Medicine and Public Health Madison Wisconsin USA; ^9^ Perlmutter Cancer Center New York University Grossman School of Medicine New York New York City USA; ^10^ School of Pharmacy University of Wisconsin Madison Wisconsin USA

**Keywords:** BMN673, carboplatin, paclitaxel, PARP, talazoparib

## Abstract

**Background:**

Inhibitors of poly(ADP‐ribose) polymerase (PARP) proteins potentiate antitumor activity of platinum chemotherapy. This study sought to determine the safety and tolerability of PARP inhibitor talazoparib with carboplatin and paclitaxel.

**Methods:**

We conducted a phase I study of talazoparib with carboplatin AUC5‐6 and paclitaxel 80 mg/m^2^ days 1, 8, 15 of 21‐day cycles in patients with advanced solid tumors. Patients enrolled using a 3 + 3 design in two cohorts with talazoparib for 7 (schedule A) or 3 days (schedule B). After induction with 4–6 cycles of triplet therapy, patients received one of three maintenance options: (a) continuation of triplet (b) carboplatin/talazoparib, or (c) talazoparib monotherapy.

**Results:**

Forty‐three patients were treated. The MTD for both schedules was talazoparib 250mcg daily. The main toxicity was myelosuppression including grade 3/4 hematologic treatment‐related adverse events (TRAEs). Dose modification occurred in 87% and 100% of patients for schedules A and B, respectively. Discontinuation due to TRAEs was 13% in schedule A and 10% in B. Ten out of 22 evaluable patients in schedule A and 5/16 patients in schedule B had a complete or partial response. Twelve out of 43 patients received ≥6 cycles of talazoparib after induction, with a 13‐month median duration of maintenance.

**Conclusion:**

We have established the recommended phase II dose of Talazoparib at 250mcg on a 3‐ or 7‐day schedule with carboplatin AUC6 and paclitaxel 80 mg/m^2^ on days 1, 8, 15 of 21‐day cycles. This regimen is associated with significant myelosuppression, and in addition to maximizing supportive care, modification of the chemotherapy component would be a consideration for further development of this combination with the schedules investigated in this study.

## Graphical abstract

Carboplatin, paclitaxel and talazoparib followed by talazoparib maintenance is feasible with appropriate supportive care, and warrants further investigation across solid tumor types, particularly those with DNA repair pathway alterations, and supports further development of PARP inhibitor maintenance strategies in the advanced disease setting.

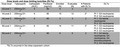

## 1 INTRODUCTION

Poly (ADP‐ribose) polymerase (PARP) 1 and 2 are nuclear enzymes that play a crucial role in initiating the DNA damage response (DDR) to maintain genomic integrity.^1^, ^2^ Inhibition of PARP1/2 leads to the accumulation of single strand breaks, causing double strand breaks that require repair by homologous recombination (HR), and tumor cells lacking BRCA1 or BRCA2, which play key roles in the HR pathway, are uniquely sensitive to PARP inhibition.^3^, ^4^

PARP inhibitors (PARPi) catalytically inhibit PARP enzymes and trap PARP on DNA, creating stable PARP‐DNA complexes that interfere with DNA replication and cause cytotoxicity.^5^ Talazoparib (BMN 673) is one of the most potent and selective PARPi both in terms of catalytic activity and PARP trapping.^6^, ^7^ Talazoparib demonstrated single agent clinical activity with an MTD of 1000mcg daily in small cell lung cancer (SCLC), pancreatic cancer and germline *BRCA1/2* deficient (gBRCA) breast and ovarian cancer in phase I/II trials,^8^, ^9^ and is FDA approved for gBRCA‐mutated advanced breast cancer based on the phase III EMBRACA trial.^10^ Other PARPi, such as olaparib, niraparib, and rucaparib, are now FDA approved for advanced gBRCA‐mutated breast, pancreatic and prostate cancer, as well as ovarian cancer with and without gBRCA mutations.^11^, ^12^, ^13^, ^14^, ^15^, ^16^, ^17^, ^18^, ^19^, ^20^

Independent of underlying DNA repair mutations, PARP inhibition sensitizes tumor cells to DNA damage‐inducing cytotoxic agents in vitro,^21^, ^22^ while PARP overexpression has been linked to chemoresistance.^23^, ^24^, ^25^, ^26^ However, while combinations of cytotoxic chemotherapy and continuously dosed PARPi have been associated with responses in early phase trials, myelosuppressive toxicities have limited tolerability.^27^, ^28^, ^29^, ^30^, ^31^ Given the goal of PARP inhibition in this setting to potentiate chemotherapy‐ induced DNA damage, pulsed dose PARP inhibition may preserve antitumor synergy while reducing toxicity.^32^, ^33^, ^34^ Carboplatin is an alkylating agent known to induce PARP activity and is potentiated by PARP inhibition in preclinical models,^22^, ^35^ and the carboplatin/paclitaxel doublet is a well‐tolerated regimen with activity across multiple solid tumor types including ovarian,^36^ head and neck,^37^ non‐small cell lung,^38^ esophageal,^39^ breast,^40^ and bladder cancers.^37^, ^41^

We hypothesized that the combination of carboplatin and paclitaxel with intermittently dosed talazoparib would improve tolerability of combination therapy, while enhancing antitumor efficacy. Here, we report the safety profile, maximum tolerated dose (MTD), and efficacy of carboplatin and paclitaxel combined with talazoparib on a 3‐ or 7‐day schedule.

## 2 PATIENTS AND METHODS

### 2.1 Eligibility

Eligible patients had a histologically confirmed advanced solid malignancy and one of the following criteria: (1) reasonable expectation of response to carboplatin and paclitaxel per treating physician (2) deleterious germline *BRCA1/2* mutation or (3) deleterious somatic *BRCA1/2* mutation (see protocol for permitted *BRCA1/2* testing in Appendix S2). There was no limit on prior lines of therapy. Prior carboplatin was not allowed unless in the neoadjuvant or adjuvant setting ≥6 months prior to enrollment, except in the case of relapsed ovarian cancer, where patients were eligible if ≥6 months since last carboplatin dose. Prior PARPi was permitted. Patients were age ≥ 18 years with an ECOG performance status of ≤2, life expectancy of ≥12 weeks, adequate organ and marrow function and measurable or evaluable disease, as defined by RECIST version 1.1. Patients with peripheral neuropathy >CTCAE grade 1 were excluded, as were patients receiving therapeutic anticoagulation or antiplatelet therapy. Patients with brain metastases were eligible if clinically stable without neurologic symptoms after local therapy for ≥4 weeks and off steroid treatment for ≥2 weeks.

### 2.2 Study design

This was a multicenter, open‐label phase 1 study. Patients were enrolled at three study sites under an IRB‐approved protocol (NCT02317874). Written informed consent was obtained from all participants prior to enrollment in the trial. The study was conducted in compliance with the Declaration of Helsinki. Patients enrolled in two independent cohorts with separate dosing schedules (Table 1), with a separate MTD determined for each schedule. For both schedules, carboplatin was administered on Day 1 and paclitaxel on Days 1, 8, 15 of a 21‐day cycle. Paclitaxel was dosed on a weekly rather than every 3 week schedule given equivalent or superior activity of this schedule across phase III studies in ovarian, non‐small cell lung, and breast cancer with more flexibility for dose modification.^38^, ^42^, ^43^, ^44^

**TABLE 1:** Dose schedules

Dose escalation schedule A			
Dose Level	Dose		
	Carboplatin (AUC) IV Day 1	Paclitaxel (mg/m2) IV Days 1, 8, 15	Talazoparib (BMN 673) (mcg) PO once daily Days 1–7
Level − 1	5	80	100
Level 1^a^	5	80	250
Level 2	6	80	250
Level 3	6	80	350
Level 4	6	80	500
Level 5	6	80	750
Level 6	6	80	1000

Dose escalation schedule B			
Dose level	Dose		
	Carboplatin (AUC) IV Day 1	Paclitaxel (mg/m2) IV Days 1, 8, 15	Talazoparib (BMN 673) (mcg) PO once daily Days 1–3
Level 1	5	80	250
Level 1^a^	6	80	250
Level 2	6	80	350
Level 3	6	80	500
Level 4	6	80	750
Level 5	6	80	1000

^a^ Starting dose level for each schedule.

Pulsed dose schedules in early phase trials of the PARP inhibitors olaparib and veliparib have ranged from 7 to 14 days,^32^, ^34^, ^45^ and given talazoparib's significantly longer mean terminal half‐life (90 ± 58 h),^46^ talazoparib was dosed daily starting on Day 1 of each cycle prior to carboplatin and paclitaxel, and continued for 7 (schedule A) or 3 days (schedule B). The schedule A dose level 1 was carboplatin AUC 5, paclitaxel 80 mg/m,^2^ and talazoparib 250 mcg once daily (25% of single agent MTD) (Table 1A). The schedule B dose level 1 was determined by the schedule A MTD as described below. Talazoparib was supplied by the Cancer Therapy Evaluation Program (CTEP) of the National Cancer Institute (NCI). Toxicity was evaluated at every cycle for the duration of enrollment for all participants who received at least one dose of study medication. CTCAE v4.0 was used for toxicity grading until March 31, 2018; CTCAE v5.0 was utilized beginning April 1, 2018. Tumor response was assessed with CT or MRI every two cycles (extended to every 6–12 weeks after 4 cycles) by RECIST version 1.1 criteria.^47^ After 4–6 cycles of combination therapy, patients with clinical benefit, defined as response or stable disease, were allowed to extend therapy as follows at the discretion of the treating provider: (a) continue triplet regimen (b) stop paclitaxel while continuing carboplatin and intermittent talazoparib, (c) stop carboplatin and paclitaxel and transition to continuous talazoparib at single agent MTD of 1000 mcg daily, or d) observation.

### 2.3 Study objectives and statistical methods

The primary objectives were to determine MTD and RP2D of (1) talazoparib on a 7‐day schedule in combination with carboplatin and paclitaxel, and (2) talazoparib on a 3‐day schedule in combination with carboplatin and paclitaxel, in patients with advanced solid malignancies.

A standard 3 + 3 design^48^ was used for dose escalation within each schedule, and after the MTD of each schedule was determined, a dose expansion at that level to a total of 12 patients was planned. Dose escalation was conducted sequentially starting with schedule A, and the starting dose level for schedule B was the MTD from Schedule A (Table 1). If at any time during the dose expansion period the lower limit of the one‐sided 90% confidence interval for the DLT rate exceeded 20%, accrual to the dose expansion cohort was to be terminated early. If no more than 33% of total patients treated at the MTD in the expanded dose cohort experienced a DLT, this would be the RP2D for each schedule.

Descriptive summaries of demographics, incidence, and severity of toxicities and disease response are presented. Dose intensity during induction for each agent was defined as percent of full dose for the number of induction cycles received. Talazoparib plasma concentrations were summarized using descriptive statistics.

### 2.4 Definition of MTD and DLT and management of toxicities

The MTD was defined as the highest dose level where <33% of patients experienced a DLT prior to cycle 2. Growth factor support was not permitted in cycle 1, but was permitted in subsequent cycles per the treating physician's discretion. DLTs were defined as: absolute neutrophil count (ANC) < 1000/mcL lasting >7 days, grade 3/4 febrile neutropenia, grade 4 thrombocytopenia lasting >7 days or requiring platelet transfusion, or grade 3/4 thrombocytopenia associated with clinically significant bleeding, grade ≥3 nausea, vomiting, diarrhea, or electrolyte abnormalities lasting >48 h despite maximal medical therapy, grade ≥3 fatigue lasting >7 days despite maximal medical therapy, any other grade ≥3 non‐hematologic toxicity, or delay in starting cycle 2 by ≥2 weeks due to toxicity. Patients must have completed at least day 1 through day 8 of planned cycle 1 treatment to be considered evaluable, unless a DLT occurred in this timeframe, in which case they would be considered evaluable and classified as a DLT regardless of whether day 8 treatment was completed. Patients who did not complete cycle 1 for reasons other than safety (e.g., withdrawal of consent, noncompliance, disease progression) OR experienced a ≥ 7‐day treatment delay within Cycle 1 for reasons other than safety, were also deemed inevaluable. Dose modification was required for grade 3/4 ANC lasting >7 days or with fever, grade 4 thrombocytopenia >7 days or requiring platelet transfusion. Dose modifications of paclitaxel were required for intolerable grade 2 or ≥ grade 3 peripheral neuropathy. Up to two dose reductions were allowed.

### 2.5 Pharmacokinetic and pharmacodynamic methods

For pharmacokinetic (PK) analyses, plasma samples were collected before and approximately 4 h after talazoparib administration on C1D1, C1D7 (schedule A), or C1D3 (schedule B), C2D1, and D1 of cycles 3–5 for cycles coinciding with radiographic assessment. On each day when pharmacodynamic (PD) or plasma samples were collected, talazoparib was dosed in clinic (prior to paclitaxel and carboplatin administration). Plasma concentrations of talazoparib were evaluated by a validated LC/MS assay. For PD analyses, whole blood samples were obtained before and approximately 4 h after talazoparib administration on C1D1, C1D3 (schedule B), or C1D7 (schedule A), and C2D1, for assessment of PAR inhibition and DNA damage response pathway activation (γH2AX, RAD51 foci) in peripheral blood mononuclear cells (PBMCs) (see Data S1).

### 2.6 Exploratory analysis of germline/somatic mutation status

Standard of care germline genetic testing was available for 30/43 patients and archival tumor tissue (available for 18/43 patients) was submitted for somatic next‐generation sequencing (NGS) with the Foundation One NGS panel. The following genes were included in the analysis: *ARID1A, ATM, ATRX, BARD1, BLM, BRCA1, BRCA2, BRIP1 (FANCJ), CDK12, CHEK1, CHEK2, FANCA, FANCD2, FANCE, FANCF, FANCL, FANCM, MRE11, MSH2, MSH6, NBN, PALB2, PPP2R2A, RAD50, RAD51B, RAD51C, RAD51D, RAD54L, WRN*, and *XRCC3*. When feasible, the NGS results were sent to the patient's treating physician for referral to genetic counseling if indicated.

## 3 RESULTS

### 3.1 Demographics and enrollment

Forty‐three patients enrolled between August of 2015 and July of 2019 (Table 2), with the most common tumor types being breast (25.6%) and gastrointestinal/hepatobiliary (25.6%) cancers. The majority had received prior chemotherapy (34.8% 1 regimen, 32.5% >2 regimens), 39.5% had germline *BRCA1/2* mutations, and 11.6% had received prior PARP inhibitor. Patients in both schedules received a median of four cycles of induction therapy with carboplatin, paclitaxel, and talazoparib (range 1–9), and 37.2% went on to receive one of the protocol‐approved maintenance therapy regimens (Table 3). Six patients (13.9%) received carboplatin plus talazoparib maintenance for median of two cycles (range 2–8), while 13 patients (30.2%) received talazoparib dosed continuously for a median of eight cycles (range 1–63). Of note, four patients who initially received carboplatin plus talazoparib maintenance were subsequently transitioned to talazoparib monotherapy (Table 3). Median duration of follow‐up on study was 4.3 months (range 0.6–56.6), and two patients remained on maintenance talazoparib at the end of the study.

**TABLE 2:** Patient demographics

	No. of Patients (%)		
Characteristic	All	Schedule A	Schedule B
N	43	23	20
Age, years			
Median	56	55	56
Range	37–76	37–70	43–76
Gender			
Male	21 (48.8)	9 (39.1)	12 (60)
Female	22 (51.1)	14 (60.8)	8 (40)
ECOG PS			
0	17 (39.5)	10 (43.4)	7 (35)
1	24 (55.8)	11 (47.8)	13 (65)
2	2 (4.6)	2 (8.7)	0 (0)
Primary cancer			
Breast	11 (25.6)	9 (39.1)	2 (10)
Pancreas	7 (16.3)	3 (13)	4 (20)
Colorectal	4 (9.3)	0 (0)	4 (20)
Non‐melanoma skin	3 (6.9)	3 (13)	0 (0)
Non‐small cell lung	3 (6.9)	0 (0)	3 (15)
Ovarian	3 (6.9)	3 (13)	0 (0)
Other^a^	12 (27.9)	5 (21.7)	7 (35)
Prior lines of cytotoxic therapy			
0	14 (32.5)	9 (39.1)	5 (25)
1	15 (34.8)	9 (39.1)	6 (30)
≥2	14 (32.5)	5 (21.7)	9 (45)
Prior PARP inhibitor	5 (11.6)	2 (8.7)	3 (15)
Germline BRCA mutation status			
gBRCA1mut	7 (16.3)	5 (21.7)	2 (10)
gBRCA2mut	10 (23.2)	7 (30.4)	3 (15)

^a^ Includes prostate (2), urothelial (2), adenoid cystic carcinoma (2), head and neck squamous cell carcinoma (1), melanoma (1), uterine leiomyosarcoma (1), and one patient each with duodenal, esophageal, and gallbladder carcinoma.

**TABLE 3:** Treatment

Characteristic	All	Schedule A	Schedule B
Induction cycles			
Median	4	4	4
Range	1–9	1–8	1–9
Maintenance cycles			
No of patients (%)	16 (37.2)	12 (52.2)	4 (20)
Talazoparib/carboplatin			
No of patients (%)^a^	6 (13.9)	4 (17.4)	2 (10)
Median cycles	2	3.5	2
Range	2–9	2–9	2–2
Talazoparib monotherapy			
No of patients (%)^a^	13 (30.2)	9 (39.1)	2 (10)
Median cycles	8	15	10
Range	1–63	1–63	2–18
Dose modification at MTD (No of patients, %)			
Ever	27 (96.4)	14 (93)	13 (100)
During induction	27 (96.4)	14 (93)	13 (100)
Chemotherapy dose reduction	27 (96.4)	14 (93)	13 (100)
BMN dose reduction	1 (3.5)	1 (7)	0 (0)
Induction % dose intensity at MTD (mean, SD)^b^			
Carboplatin	91 (9)	93 (7)	89 (10)
Paclitaxel	68 (17)	69 (18)	68 (17)
BMN	92 (20)	89 (24)	94 (14)
Supportive care (No of patients, %)			
Growth factor	15 (34.8)	11 (47.8)	4 (20)
Any transfusion	14 (32.5)	6 (26.1)	8 (40)
pRBC transfusion	12 (27.9)	6 (26.1)	6 (30)
Platelet transfusion	2 (4.6)	0 (0)	2 (10)
Reason for treatment discontinuation (No of patients, %)			
Disease progression	26 (60.4)	14 (60.8)	12 (60)
Adverse event	5 (11.6)	3 (13)	2 (10
Intercurrent illness	3 (6.9)	1 (4.3)	2 (10)
Death on study	1 (2.3)	0 (0)	1 (5)
Study completion/transition to commercial drug	2 (4.66)	2 (8.7)	0 (0)
Other	6 (13.9)	3 (13)^c^	3 (15)^d^

^a^ Patients who received both talazoparib/carboplatin and subsequent talazoparib monotherapy maintenance are reported in both groups.

^b^ Percent of full dose based on number of induction cycles.

^c^ Schedule A (one patient each): Physician's decision, Patient not tolerating therapy, and Switched to alternative treatment.

^d^ Schedule B (one patient each): Refused further treatment, late determination of eligibility, and treatment held for more than 3 weeks.

### 3.2 Toxicity

In the schedule A dose escalation, 0/3 patients at dose level 1, 0/3 patients at dose level 2, and 2/3 patients at dose level 3 experienced DLTs (Table 4). Thus, the MTD for schedule A was determined to be dose level 2 (carboplatin AUC 6, paclitaxel 80 mg/m,^2^ talazoparib 250 mcg day 1–7). In the schedule A MTD expansion cohort, 2/9 additional evaluable patients experienced DLTs, remaining below the 33% threshold at the MTD, and confirming dose level 2 as the MTD for schedule A. The reported DLTs in schedule A were grade 3 and 4 neutropenia and grade 4 febrile neutropenia (Table 4). Five out of 23 patients were unevaluable for DLT due to omission of cycle 1 day 8 paclitaxel without meeting protocol‐defined DLT toxicity criteria. In the dose level 1 escalation cohort, this included one patient with grade 3 nausea in the setting of suboptimal antiemetic support, one patient who was hospitalized with a urinary tract infection without fever or neutropenia deemed unrelated to study treatment, and one patient with grade 2 total bilirubin elevation ultimately identified as Gilbert's disease. In the dose level 2 escalation cohort this included one patient with grade 3 abdominal pain and one patient hospitalized with a venous thromboembolic event, both deemed to be attributable to disease progression by treating investigators and PI.

**TABLE 4:** Dose levels and dose limiting toxicities (DLTs)

Dose level	Talazoparib	Carboplatin (AUC)	Paclitaxel (mg/m2)	Enrolled (N)	Evaluable (N)	# Patients with DLTs	DLTs
A/Level 1	250mcg D1‐7	5	80	5	3	0	
A/Level 2	250mcg D1‐7	6	80	15	12	2^a^	Pt 1: G3 neutropenia Pt 2: G4 neutropenia
A/Level 3	350mcg D1‐7	6	80	3	3	2	Pt 1: G3 febrile neutropenia Pt 2: G3 neutropenia
B/Level 1	250mcg D1‐3	6	80	14	11	3^a^	Pt 1: G3 neutropenia Pt 2: G3 neutropenia Pt 3: G3 neutropenia
B/Level 2	350mcg D1‐3	6	80	6	6	2	Pt 1: G3 febrile neutropenia, G4 neutropenia, G4 thrombocytopenia Pt 2: G4 neutropenia

^a^ DLTs occurred in the dose expansion cohort.

In the schedule B dose escalation, 0/3 patients experienced DLTs at dose level 1, however 2/6 experienced DLTs at dose level 2, exceeding the MTD (Table 4), with subsequent dose expansion at dose level 1. In the overall schedule B dose level 1 (MTD) cohort comprising both escalation and expansion cohorts, 3/11 evaluable patients experienced DLTs. Since the prespecified 33% or less threshold to confirm the MTD would still have been met if a 12th evaluable patient were to enroll and experience a DLT, enrollment in this schedule was not re‐opened. Thus, the confirmed MTD was dose level 1 (carboplatin AUC 6, paclitaxel 80 mg/m,^2^ talazoparib 250 mcg day 1–3). All three DLTs at dose level 1 (MTD) were grade 3 neutropenia, while the DLTs at dose level 2 included grade 3 febrile neutropenia, grade 4 neutropenia and grade 4 thrombocytopenia (Table 4). Three out of 20 patients in schedule B were unevaluable for DLT. This included one patient in the dose level 1 expansion cohort who missed the third dose of talazoparib due to administrative error, and one patient in the dose level 1 escalation cohort for whom timing of follow‐up CBC was not within the window to determine duration of neutropenia. One additional patient in the dose level 1 escalation cohort did not receive study therapy due to determination of ineligibility post‐registration so was not included in DLT, toxicity and efficacy analyses.

Common AEs of any grade experienced by at least 10% of participants and at least possibly related to the study medications are summarized in Table 5. Myelosuppression was the most common toxicity, and over half of the participants experienced a grade 3/4 hematologic toxicity, but febrile neutropenia was uncommon (Table 5). Fatigue, nausea, and diarrhea were also common but primarily grade 1/2. Peripheral neuropathy was common, but mild in nature. Growth factor support was administered to 35% of patients after cycle 1, in the form of non‐PEGylated G‐CSF on an intermittent weekly schedule, and 32% of patients required at least one transfusion (Table 3), primarily during the induction phase of treatment (Table S1). Dose delay/modification occurred in 86.6% and 100% of patients treated at the MTD for schedules A and B, respectively, however mean dose intensity during the induction phase was 91% for carboplatin, 68% for paclitaxel, and 92% for talazoparib, and the rate of treatment discontinuation due to adverse events was low, occurring in only three patients (13%) in schedules A and two (10%) in B (Table 3). One patient developed myelodysplastic syndrome (MDS) while on maintenance talazoparib, 16 months after starting induction therapy. While MDS is a rare (0.3%) complication of talazoparib therapy,^46^ the patient had prior exposure to alkylating agents including temozolomide 4 years prior and cisplatin 2 years prior to the MDS diagnosis, which was investigator attributed as more likely related to these prior therapies. One patient experienced a grade 5 intracranial hemorrhage associated with persistent grade 3 thrombocytopenia in the setting of extensive malignant bone marrow involvement. Non‐contrast CT was concerning for brain metastases, but it was decided to attribute patient's death as at least possibly related to study therapy.

**TABLE 5:** Toxicities at least possibly related to treatment. Number of patients (%); n = 42^a^

Event	Any grade	Grade 1	Grade 2	Grade 3	Grade 4
Any adverse event	42 (100)	41 (98)	39 (93)	41 (98)	14 (33)
Neutrophil count decreased	38 (90)	14 (33)	29 (69)	27 (64)	13 (31)
Anemia	38 (90)	16 (38)	34 (81)	22 (52)	0
Platelet count decreased	33 (79)	25 (60)	27 (64)	10 (24)	5 (12)
Fatigue	29 (69)	25 (60)	12 (29)	6 (14)	0
Nausea	22 (52)	19 (45)	7 (17)	2 (5)	0
Diarrhea	19 (45)	15 (36)	4 (10)	3 (7)	0
Lymphocyte count decreased	17 (40)	12 (29)	12 (29)	9 (21)	0
Peripheral neuropathy	12 (29)	12 (29)	3 (7)	0	0
Vomiting	11 (26)	8 (19)	2 (5)	2 (5)	0
Alopecia	11 (26)	6 (14)	5 (12)	0	0
Anorexia	10 (24)	8 (19)	3 (7)	1 (2)	0
Constipation	9 (21)	8 (19)	1 (2)	0	0
Hypophosphatemia	8 (19)	2 (5)	6 (14)	2 (5)	0
Mucositis	8 (19)	7 (17)	2 (5)	0	0
Myalgias	7 (17)	6 (14)	1 (2)	1 (2)	0
Abdominal pain	6 (14)	4 (10)	1 (2)	2 (5)	0
Hypomagnesemia	6 (14)	4 (10)	2 (5)	1 (2)	0
Dizziness	6 (14)	6 (14)	0	0	0
Rash	6 (14)	6 (14)	0	0	0
Hypertension	5 (12)	0	4 (10)	1 (2)	0
ALT increased	5 (12)	3 (7)	2 (5)	0	0
Dysgeusia	5 (12)	3 (7)	2 (5)	0	0
Dyspepsia	5 (12)	3 (7)	2 (5)	0	0
Headache	5 (12)	5 (12)	0	0	0
Alkaline phosphatase increased	4 (10)	3 (7)	1 (2)	0	0
Dyspnea	4 (10)	3 (7)	1 (2)	0	0
Weight loss	4 (10)	4 (10)	2 (5)	0	0
Dehydration	4 (10)	1 (2)	3 (7)	0	0
Hypoalbuminemia	4 (10)	1 (2)	3 (7)	0	0
Sinus tachycardia	4 (10)	4 (10)	0	0	0

^a^ One patient in schedule B who did not receive any study medication due to determination of ineligibility post‐registration is not included in this analysis.

### 3.3 Clinical efficacy

Among the 38 patients evaluable for disease response, there were 3/38 complete (CR) and 12/38 partial (PR) responses. All three patients with CRs were enrolled in schedule A. An additional 17 patients across both cohorts had stable disease (SD) (Table 6). Among patients with prior PARP inhibitor exposure, 1/5 had a PR, 3/5 had SD, and 1/5 had progressive disease as best response.

**TABLE 6:** Efficacy

	Number of patients (%)		
	All	Schedule A	Schedule B
Best response			
CR	3/38 (7.9)	3/22 (13.6)	0/16 (0)
PR	12/38 (31.6)	7/22 (31.8)	5/16 (31.2)
SD	17/38 (44.7)	9/22 (40.9)	8/16 (50)
PD	6/38 (15.7)	3/22 (13.6)	3/16 (18.7)
Clinical benefit (CR, PR, or SD)	32/38 (84.2)	19/22 (86.3)	13/16 (81.2)
Best response, prior PARPi			
CR	0/5 (0)		
PR	1/5 (20)		
SD	3/5 (60)		
PD	1/5 (20)		
Clinical benefit (CR, PR, or SD)	4/5 (80)		
Patients receiving ≥6 cycles of maintenance therapy			
Total	12/38 (31.6)	10/22 (45.4)	2/16 (12.5)
Duration of maintenance (months)			
Median	12.86		
Range	5.5–55.5+		
Tumor type			
Breast	5		
Ovarian	2		
Cutaneous SCC	2		
Adenoid cystic carcinoma	1		
Uterine leiomyosarcoma	1		
Urothelial	1		

Twelve patients received at least six cycles of talazoparib maintenance therapy (with or without carboplatin) after completing at least four cycles of initial triplet therapy. In this group, the median duration of maintenance therapy was 12.9 months, with a range from 5.5 to 55.5+ months (Table 6); two patients remained on maintenance talazoparib at time of study completion. These patients represented a range of tumor types with the most common being breast, ovarian, and cutaneous squamous cell carcinoma (Table 6).

### 3.4 PK/PD

The mean plasma concentrations of talazoparib 4 h after the first dose of cycle 1 were 0.612 ng/ml (44.1 CV%) and 0.58 ng/ml (44.9 CV%) for schedule A and B, respectively. For Schedule A, the mean talazoparib plasma concentration on C1D7 4 h post‐dose was 2.95 ng/ml (44.8 CV%). For Schedule B, the mean talazoparib plasma concentration was 2.19 (55.7 CV%). Plasma concentrations 4 h following the first dose of cycles 2 and 3 were similar for both schedules.

Pharmacodynamic analyses in schedule A demonstrated no significant change in markers of DNA damage response (Rad51 foci and γH2AX intensity) or PARP inhibition (PAR intensity) in peripheral blood mononuclear cells (PBMCs) from baseline to C1D7 or C2D1 (Figure S1) and as such no pharmacodynamic analysis of schedule B was undertaken.

### 3.5 Germline/somatic DNA damage response pathway alterations

An exploratory analysis of DNA damage response (DDR) pathway mutation status was conducted for patients with standard of care germline testing (30/43 patients) and/or sufficient archival tumor tissue for Foundation One NGS panel sequencing (18/43 patients), which does not distinguish between germline and somatic mutations (Figure S2). Seven patients had *gBRCA1* and 10 patients had *gBRCA2* mutations. Two patients had germline mutations in other DNA damage response (DDR) pathway genes (*ATM* and *ARID1A*), and five patients had deleterious mutations in DDR pathway genes (*PALB2, FANCA, ARID1A, BRIP1*, and *MSH6*) identified on Foundation One NGS. Two out of 3 complete responses and 8/10 partial responses occurred in patients with DDR pathway alterations (Figure S2). Six out of 12 patients who received ≥6 cycles of maintenance therapy had *BRCA1/2* alterations on germline testing, while two additional patients in this group had DDR pathway alterations detected on Foundation One NGS, including a patient with cutaneous squamous cell cancer and a pathogenic *PALB2* mutation and a patient with breast cancer and a pathogenic *FANCA* mutation.

## 4 DISCUSSION

This phase I study identified a recommended phase 2 dose of carboplatin AUC 6, paclitaxel 80 mg/m^2^, and talazoparib 250 mcg for both the 3‐day and 7‐day schedule. However, this regimen was associated with significant myelosuppression during the induction phase, with a third of patients receiving growth factor after cycle 1, and a third requiring transfusion support. Dose modifications were also frequently required, most commonly omitting one weekly paclitaxel dose. Unexpectedly, there was no evidence of improved tolerability for the 3‐day versus 7‐day schedule. However, the rate of febrile neutropenia was low, and supportive care allowed patients to complete induction chemotherapy with relatively preserved chemotherapy intensity and few discontinuations due to toxicity, similar to what has been reported in other studies evaluating PARPi with chemotherapy.^28^, ^45^ Pharmacokinetic analyses revealed no differences in plasma concentration of talazoparib between schedules.

Germline *BRCA1/2* mutations are well‐established predictive biomarkers of PARPi sensitivity,^3^, ^4^, ^49^ and predict sensitivity to PARPi monotherapy in metastatic breast^10^, ^50^ and ovarian^17^, ^51^, ^52^ cancer, and to PARPi maintenance therapy in metastatic ovarian^15^, ^18^ and pancreatic^14^ cancer. Subsequent trials have identified additional somatic or germline alterations in DNA damage repair (DDR) pathway genes associated with PARPi sensitivity^53^ including germline *PALB2* mutations and somatic *BRCA1/2* mutations in metastatic breast cancer^54^ and germline or somatic *BRCA1/2* and *ATM* alterations in metastatic castrate resistant prostate cancer^13^’.^55^ In the GOG‐3005 study investigating veliparib in combination with chemotherapy, the benefit of the PARP inhibitor was also extended to all patients with newly diagnosed ovarian cancer.^56^

The majority of patients on this study with objective responses did have DDR pathway genomic alterations (Figure S2). However, the subset of 12 patients who received at least six cycles of maintenance therapy after induction included five patients with solid tumors in which PARP inhibition has been less well studied, including two with cutaneous squamous cell carcinoma, one of whom had a somatic pathogenic PALB2 alteration. This supports our clinical observations, which suggest further consideration of development of PARPi in other solid tumor types beyond breast and ovarian cancer, particularly those with DDR alterations.

The phase III BROCADE3 trial found that the addition of intermittent veliparib to carboplatin plus paclitaxel in advanced gBRCA‐mutated breast cancer with 0–2 prior lines of therapy improved PFS and duration of response,^57^ but in contrast to the induction‐maintenance strategy utilized in our study, patients in BROCADE received a median of 10 cycles of cytotoxic chemotherapy, with only 41% of patients in the veliparib arm and 34% of patients in the placebo arm transitioning to blinded veliparib/placebo monotherapy prior to progression. Our data suggest that patients who complete 4–6 cycles of induction combination therapy without disease progression may be able to transition to maintenance monotherapy, which also draws support from the maintenance PARPi post‐platinum induction approach in advanced pancreatic^14^ and ovarian^15^, ^18^ cancer. Furthermore, we observed responses with this strategy in tumor types beyond breast and ovarian cancer in our cohort, suggesting that it may have value across HRD‐deficient tumors.

Overall, the induction‐maintenance design in our study did facilitate a shorter duration of cytotoxic chemotherapy in the advanced disease setting, at the expense of significant toxicity during the induction phase in both schedules. The exploratory analysis of somatic and germline DDR mutation status, available for the majority of enrolled patients, did demonstrate disease responses in patients with DDR alterations in tumor types such as head and neck squamous cell cancer in which the impact on DDR mutation status on PARPi/platinum sensitivity is less well characterized. Limitations include the finding that the MTD for talazoparib was significantly lower for both the 3‐ and 7‐day schedules compared to monotherapy PARPi studies. However, while maximal PARP inhibition is seen with doses ≥600mcg in the monotherapy setting, pharmacodynamic studies demonstrated significant inhibition at doses as low as 200 mcg, suggesting that the MTD here would be expected to result in significant target engagement.^8^

In conclusion, the combination of talazoparib with carboplatin and paclitaxel is feasible with an intermittent, lower dose schedule of talazoparib and appropriate supportive care. However, modification of the cytotoxic chemotherapy component of the regimen would be a consideration in further development, given the challenging toxicity profile. Future studies of PARPi combined with single agent platinum or other DNA damaging chemotherapy may maximize the effect of DNA damage with a more favorable toxicity profile, potentially expanding use of PARPi to other solid tumors. Patient selection for PARPi based on genomic alterations affecting DNA repair should continue to be explored in future trials.

## CONFLICT OF INTEREST

Ticiana A. Leal: Consulting ‐ Boehringer‐Ingelheim, Jazz Pharmaceuticals, Genentech, Lilly; Advisory Boards ‐ AstraZeneca, EMD Serono, Merck, Boehringer‐Ingelheim, Blueprint, Bayer, Janssen, Mirati. Lisa M. Barroilhet: Advisory boards ‐ Clovis, Astrazeneca, Kiayatec. Kari B. Wisinski: Research support ‐ Pfizer, Context, Novartis; Scientific Advisory boards ‐ Eisai, AstraZeneca, Pfizer, Sanofi, Daiichi Sankyo, Novartis. The other authors declare no potential conflict of interest.

## AUTHOR CONTRIBUTIONS

Ticiana A. Leal: Conceptualization, resources, funding acquisition, investigation, methodology, supervision, writing‐original draft, writing‐review, and editing. Marina N. Sharifi: Data curation, formal analysis, investigation, visualization, methodology, writing‐original draft, writing‐review, and editing. Nancy Chan: Conceptualization, resources, methodology, writing‐review, and editing. Robert Wesolowski: Conceptualization, resources, methodology, writing‐review, and editing. Anita A. Turk: Data curation, formal analysis, visualization, methodology, writing‐review, and editing. Justine Y. Bruce: Resources, writing‐review, and editing. Ruth M. O'Regan: Resources, writing‐review, and editing. Jens Eickhoff: Resources, methodology, writing‐review, and editing. Lisa M. Barroilhet: Resources, writing‐review, and editing. Jyoti Malhotra: Conceptualization, resources, methodology, writing‐review, and editing. Janice Mehnert: Resources, writing‐review, and editing. Eugenia Girda: Resources, writing‐review, and editing. Elizabeth Wiley: Resources and investigation. Natalie Schmitz: Resources, formal analysis, investigation, visualization, methodology, writing‐review, and editing. Shannon Andrews: Resources, formal analysis, investigation, and methodology. Glenn Liu: Conceptualization, resources, methodology, writing‐review, and editing. Kari B. Wisinski: Conceptualization, resources, supervision, funding acquisition, methodology, writing‐original draft, writing‐review, and editing.

## ETHICS STATEMENT

This study was conducted under an IRB‐approved protocol (NCT02317874). Written informed consent was obtained from all participants prior to enrollment in the trial. The study was conducted in compliance with the Declaration of Helsinki.

## Supporting information


Appendix S1

Appendix S2

## Data Availability

Data available on request due to privacy/ethical restrictions
