# Supplementary material for: A phase I study of talazoparib (BMN 673) combined with carboplatin and paclitaxel in patients with advanced solid tumors (NCI9782)
Source: Cancer Med. 2022 Apr 8;11(21):3969–81. doi: 10.1002/cam4.4724 (PMC9636507; doi:10.1002/cam4.4724)
Supplement: Supplementary file 1 — Appendix S1 [file CAM4-11-3969-s001.docx]

**Supplementary Methods**

*Assessment of PAR inhibition and DNA damage response pathway activation in peripheral blood mononuclear cells*

Peripheral blood mononuclear cells (PBMCs) were obtained before and approximately 4 hours after talazoparib administration on C1D1, C1D3 (schedule B) or C1D7 (schedule A), and C2D1. PBMCs were fixed (BD Cytofix^TM^), permeabilized with 0.1% triton, then stained with fluorescently labeled primary antibodies, including anti-PAR (clone 10H, EMD-Millipore), anti-RAD51 (clone 14B4, Invitrogen) and anti-yH2AX (clone N1-431, BD Biosciences) as well as the DRAQ5 nuclear stain (eBioscience). Samples were imaged using an ImageStreamX MarkII (Luminex Corporation, Austin, TX) which combines single cell fluorescence detection with high-resolution microscopy, and single cell mean fluorescence intensity (yH2AX, PAR) or number of fluorescent nuclear foci (RAD51) were quantified.

**Supplementary Table 1. Treatment**

| Characteristic | All | Schedule A | Schedule B |
| --- | --- | --- | --- |
| Dose modification at MTD  (No of patients, %) |  |  |  |
| Ever | 27 (96.4) | 14 (93) | 13 (100) |
| During induction | 27 (96.4) | 14 (93) | 13 (100) |
| Chemotherapy dose reduction | 27 (96.4) | 14 (93) | 13 (100) |
| Carboplatin dose reduction | 18 (64.2) | 9 (60) | 9 (69) |
| Paclitaxel dose reduction | 27 (96.4) | 14 (93) | 13 (100) |
| BMN dose reduction | 1 (3.5) | 1 (7) | 0 (0) |
| Transfusion support by treatment phase (No of patients, %) |  |  |  |
| During triplet only | 10 (23.2) |  |  |
| Triplet and BMN maintenance | 3 (6.9) |  |  |
| Carboplatin/BMN maintenance | 1 (2) |  |  |
| Growth factor support by treatment phase (No of patients, %) |  |  |  |
| During triplet only | 14 (32.5) |  |  |
| Carboplatin/BMN maintenance | 1 (2) |  |  |

**
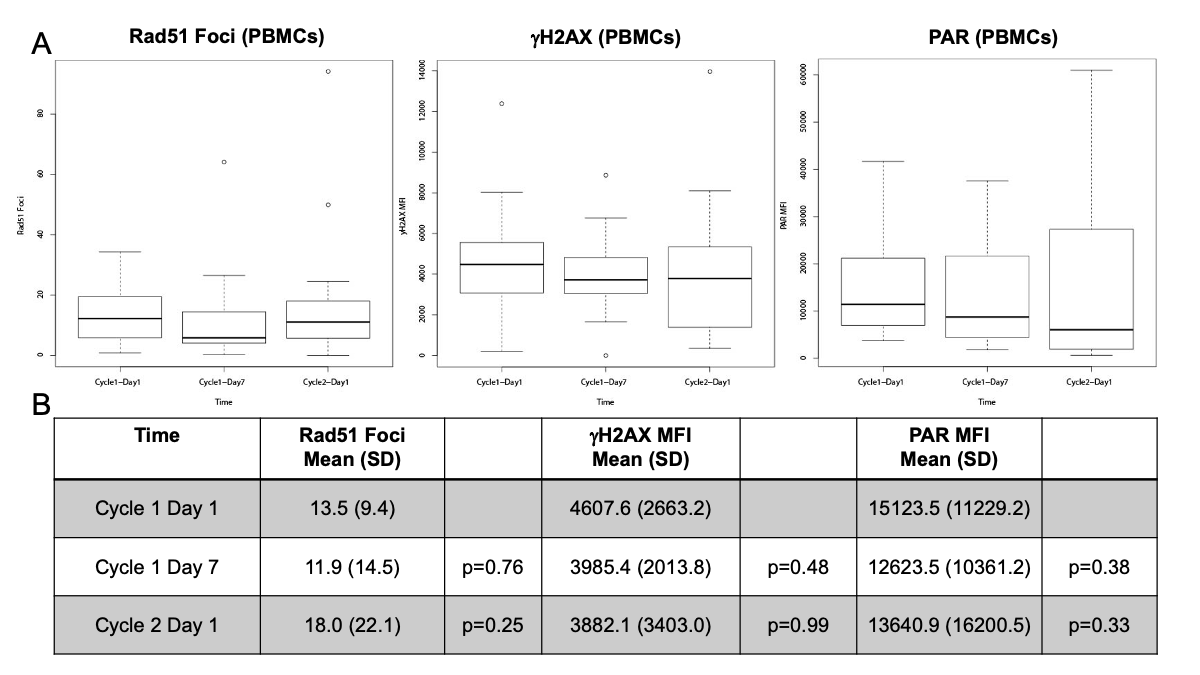
**

**Supplementary Figure 1. Pharmacodynamics of talazoparib dosed on a 7-day schedule.** Peripheral blood mononuclear cells (PBMCs) were isolated from patients enrolled on the schedule A dose escalation and dose expansion cohorts prior to talazoparib administration on Cycle 1 Day 1, and 4 hours after talazoparib administration on Cycle 1 Day 7 and Cycle 2 Day 1 and markers of DNA damage (Rad51 Foci, γH2AX) and PARP activity (PAR) were quantified by immunofluorescent staining. **(A)** Mean number of Rad51 foci (left), γH2AX mean fluorescence intensity (MFI) (middle, and PAR MFI (right) in PBMCs from each patient at each time point. **(B)** There was no significant difference in markers of DNA damage or PAR activity in PBMCs from baseline to Cycle 1 Day 7 or from Cycle 1 Day 7 to Cycle 2 Day 1 in schedule A patients.

**Supplementary Figure 2. Swimmers plot of response and DNA damage repair mutation status**. Treatment as well as time and type of best response (CR: complete response, PR: partial response, SD: stable disease, PD: progressive disease) are noted, along with dose schedule (A: schedule A, B: schedule B, DL1: dose level 1, DL2: dose level 2, DL3: dose level 3). All known germline or somatic alterations affecting the DNA damage repair pathway(see methods for list of included genes) are noted, with germline alterations in bold. Somatic alterations include variant allele frequency (VAF) where available.
