# Supplementary material for: A phase I study of talazoparib (BMN 673) combined with carboplatin and paclitaxel in patients with advanced solid tumors (NCI9782)
Source: Cancer Med. 2022 Apr 8;11(21):3969–81. doi: 10.1002/cam4.4724 (PMC9636507; doi:10.1002/cam4.4724)
Supplement: Supplementary file 2 — Appendix S2 [file CAM4-11-3969-s002.pdf]

DATE: November 5, 2020

TO: CTEP Protocol and Information Office

FROM: Kari Wisinski, MD

SUBJECT: Amendment in response to Dr. Ivy's request for rapid amendment (RRA) dated 11/3/2020.

SUMMARY OF CHANGES – Protocol

I. CTEP's Request for Rapid Amendment:

#	Section	Comments
1.	Header, Title Page	Updated protocol version date.
2.	7.1.1 CAEPR	As requested in the rapid amendment, the CAEPR was replaced with version 2.4, October 24, 2020. The following change was made:<br>• Deleted Risk:<br>○ Rare but Serious: Typhlitis

SUMMARY OF CHANGES – Consent Form

#	Section	Comments
1.	Header	Updated protocol version date.
2.	*What possible risks can I expect from taking part in this study?*	In response to Dr. Ivy's request for rapid amendment, the following change to the risks of talazoparib (BMN 673) was made:<br>• Deleted Risk:<br>• Rare: Swelling of the bowels which may require surgery

NCI Protocol #: 9782

Local Protocol #: NCI 9782

ClinicalTrials.gov Identifier: NCT02317874

TITLE: A Phase 1 Study of Talazoparib (BMN 673) in Combination with Carboplatin and Paclitaxel in Patients with Advanced Solid Tumors

Corresponding Organization: **LAO-MD017** / JHU Sidney Kimmel Comprehensive Cancer Center LAO

Coordinating Organization: University of Wisconsin Carbone Cancer Center AO

Study Chair:
Kari Wisinski, MD
University of Wisconsin Carbone Cancer Center
1111 Highland Avenue, WIMR 6033
Madison, WI 53705-2275
Phone: 608-263-6222
Fax: 608-237-2322
kbwisinski@medicine.wisc.edu

Co-Study Chair:
Ticiana Leal, MD
University of Wisconsin Carbone Cancer Center
1111 Highland Avenue, WIMR 3037
Madison, WI 53705-2275
Phone: 608-263-6222
Fax: 608-237-2322
tbleal@medicine.wisc.edu

Participating ETCTN Organizations:

LAO-11030 / University Health Network Princess Margaret Cancer Centre LAO
Participation limited to:
- **NJ066** / Rutgers Cancer Institute of New Jersey

LAO-MD017 / JHU Sidney Kimmel Comprehensive Cancer Center LAO
Participation limited to:
- **WI020** / University of Wisconsin Hospital and Clinics

LAO-OH007 / Ohio State University Comprehensive Cancer Center LAO
Participation limited to:
- **OH007** / Ohio State University Comprehensive Cancer Center

UM1 Co-Principal Investigators:
Glenn Liu, MD
University of Wisconsin Carbone Cancer Center
1111 Highland Avenue, WIMR 7057
Madison, WI 53705-2275
Phone: 608-263-8610
Fax: 608-237-2322
gxl@medicine.wisc.edu

Nancy Chan, MD
Rutgers Cancer Institute of New Jersey
195 Little Albany St.
New Brunswick, NJ 08901
Phone: 732-235-9692
Fax: 732-235-7493
Email: nancy.chan@cinj.rutgers.edu

Jyoti Malhotra, MD
Rutgers Cancer Institute of New Jersey
195 Little Albany St.
New Brunswick, NJ 08901
Phone: 732-235-7521
Fax: 732-235-8808
Email: Jyoti.malhotra@rutgers.edu

Statistician:
Jens Eickhoff, PhD
600 Highland Ave, K6/434 CSC Madison, WI 53792-4675
Phone: (608) 265-5376
(608) 265-5578 (Fax)
eickhoff@biostat.wisc.edu

UW Program Manager:
Jennifer Heideman, BSN, RN
Phone: (608) 263-6222
(608) 237-2322 (Fax)
jlv@medicine.wisc.edu

UW Program Administrator:
Kara Mattox, RN, BN
Phone: (608) 263-2944
(608) 237-2322 (Fax)
kmattox@wisc.edu

Johns Hopkins LAO-MD017 Contact:
Judy Murray, CCRC
201 N. Broadway Street, Viragh 9131
Baltimore, MD 21287
410-955-4044
jmurra33@jhmi.edu

UW Cancer Therapy Discovery and Development (CTD2) Research Nurses:
Kim Binger, BSN, RN (km1@medicine.wisc.edu) Heidi
Brandon, BSN, RN (hkb@medicine.wisc.edu)
Abbi Dunning, BSN, RN (adunning3@wisc.edu)
Chris Feierabend, BSN, RN (caf@medicine.wisc.edu)
Karen Schroeder, BSN, RN (kschroeder@medicine.wisc.edu)
Stephanie West, BSN, RN (swest2@wisc.edu)
Phone: (608) 263-6222
(608) 237-2322 (Fax)

UW Coordinators:
Amy Forsyth, BS (ajforsyth@medicine.wisc.edu)
Jess Gunderson, MSc (jjgunder@uwcarbone.wisc.edu)
Jeff Ockuly, BS (jcockuly@uwcarbone.wisc.edu)
Amy Ticknor, BS (aticknor@wisc.edu)
Amber Williquette, BS (awilliquette@wisc.edu)
Corinne Wilschke, BS (wilschke@wisc.edu)
Phone: (608) 263-6222
(608) 237-2322 (Fax)

NCI-Supplied Agent(s): Talazoparib (BMN 673) (NSC 771561)
IND #: 119558
IND Sponsor: DCTD, NCI
Commercial- Supplied Agent(s): Carboplatin (NSC 241240) and Paclitaxel (NSC 673089)

**Protocol Type / Version # / Version Date:**	Original/ Version # 1/ May 26, 2015<br>Amendment 1/ June 15, 2015<br>Amendment 2/ December 15, 2015<br>Amendment 3/ May 20, 2016<br>Amendment 4/ May 27, 2016<br>Amendment 5/ March 17, 2017<br>Amendment 6/ May 22, 2017<br>Amendment 7/ September 20, 2017<br>Amendment 8/ January 22, 2018<br>Amendment 9/ July 18, 2018<br>Amendment 10/ January 14, 2019<br>Amendment 11/ July 16, 2019<br>Amendment 16/ July 27, 2020<br>Amendment 17 / September 29, 2020 (disapproved)<br>Amendment 18 / October 14, 2020<br>Amendment 19 / November 5, 2020

SCHEMA

At each dose level, carboplatin will be administered intravenously on day 1 and paclitaxel intravenously on days 1, 8, 15 of a 21-day cycle. Talazoparib (BMN 673) will be dosed orally starting on day 1 of each cycle prior to carboplatin and paclitaxel infusion. The starting dose of Talazoparib (BMN 673) for schedule A will be at 25% of the single agent MTD (250 mcg once daily). Two MTDs will be determined from this phase I trial, one for each schedule.

In schedule A, Talazoparib (BMN 673) will be administered once daily for 7 days total. In schedule B, Talazoparib (BMN 673) will be administered once daily for 3 days total. Dose escalation for the two schedules will be conducted sequentially, starting with schedule A. Once the MTD for schedule A is determined, a six patient dose expansion at the MTD will occur. If no more than 4 of the evaluable 12 total patients treated at the MTD experienced a DLT, then the starting dose level for schedule B will be the MTD determined from Schedule A. If more than 4 patients experience a DLT with the dose expansion cohort at the MTD for schedule A, a conference call will be held with the sponsor to establish the starting dose for schedule B. If dose level -1 in Schedule A exceeds the MTD, then dose level -1 will be used as the starting dose for schedule B.

As of Amendment 7 (dated 9/20/17):

In Schedule A, 14 total patients have started treatment at dose level 2 (carboplatin AUC 6, paclitaxel 80mg/m2 and Talazoparib (BMN 673) 250mcg). Three were not evaluable in cycle 1 for DLT. Of the 11 evaluable patients, two experienced DLT (both with grade 3/4 neutropenia lasting > 7 days), 9 completed cycle 1 without DLT. The final patient has signed consent and is planned to start protocol therapy. Thus, no more than 3 patients will experience DLT at DL2 for schedule A. This meets the protocol criteria for MTD for schedule A being defined as dose level 2 which will be the starting dose level for schedule B.

After schedule B reaches the MTD, a dose expansion of 6 patients at the MTD will occur. The MTD for each schedule will be used as the recommended phase 2 dose. In the unlikely event that the MTD determined from schedule B is lower than the MTD determined from schedule A, then the MTD from schedule B will be used as the recommended phase 2 dose for schedule A.

At any time after 4-6 cycles of combination therapy, for patients with clinical benefit defined as response or stable disease, extension of therapy is allowed with a) carboplatin, paclitaxel and Talazoparib (BMN 673), b) carboplatin and Talazoparib (BMN 673) without paclitaxel, c) Talazoparib (BMN 673) daily continuous dosing or d) observation without therapy per discretion of the treating provider. If the patient is continued on a) or b), subsequent change to c) at later timepoints is also allowed.

Dose Escalation Schedule A			
**Dose Level**	**Dose**		
	**Carboplatin (AUC) IV Day 1**	**Paclitaxel (mg/m^2) IV Days 1, 8, 15**	**Talazoparib (BMN 673) (mcg) PO once daily Days 1-7****
Level -1	5	80	100
Level 1*	5	80	250
Level 2	6	80	250
Level 3	6	80	350
Level 4	6	80	500
Level 5	6	80	750
Level 6	6	80	1000
*Starting dose level for Schedule A<br>** For schedule B Talazoparib (BMN 673) will be administered days 1-3			

Dose Escalation Schedule B			
**Dose Level**	**Dose**		
	**Carboplatin (AUC) IV Day 1**	**Paclitaxel (mg/m^2) IV Days 1, 8, 15**	**Talazoparib (BMN 673) (mcg) PO once daily days 1-3**
Level -1	5	80	250
Level 1*	6	80	250
Level 2	6	80	350
Level 3	6	80	500
Level 4	6	80	750
Level 5	6	80	1000
*.Starting dose level			

TABLE OF CONTENTS

SCHEMA 4

1 OBJECTIVES 9
1.1 Primary Objectives 9
1.2 Secondary Objectives 9
1.3 Exploratory Objectives 9

2 BACKGROUND 10
2.1 Advanced Solid Tumor in Adults 10
2.2 Talazoparib (BMN 673) 10
2.3 Carboplatin and Paclitaxel Combination 16
2.4 Rationale for Combination 18
2.5 Correlative Studies Background 19

3 PATIENT SELECTION 21
3.1 Eligibility Criteria 21
3.2 Exclusion Criteria 22
3.3 Inclusion of Women and Minorities 24

4 REGISTRATION PROCEDURES 25
4.1 Investigator and Research Associate Registration with CTEP 25
4.2 Site Registration 26
4.3 Patient Registration 27
4.4 General Guidelines 28

5 TREATMENT PLAN 30
5.1 Agent Administration 30
5.2 Definition of Dose-Limiting Toxicity 37
5.3 General Concomitant Medication and Supportive Care Guidelines 40
5.4 Duration of Therapy 41
5.5 Duration of Follow Up 42
5.6 Criteria for Removal from Study 42

6 DOSING DELAYS/DOSE MODIFICATIONS 43
6.1 Dose Modifications for Hematological Toxicity 45
6.2 Dose modifications for non-hematological toxicity 48

7 ADVERSE EVENTS: LIST AND REPORTING REQUIREMENTS 57
7.1 Comprehensive Adverse Events and Potential Risks List(s) (CAEPRs) 57
7.2 Adverse Event Characteristics 59
7.3 Expedited Adverse Event Reporting 60

7.4 Routine Adverse Event Reporting 62
7.5 Secondary Malignancy 62
7.6 Second Malignancy 63

8 PHARMACEUTICAL INFORMATION 64
8.1 CTEP IND Agent: Talazoparib (BMN 673) (NSC # 771561) 64
8.2 Commercial Agent: Paclitaxel Availability 66
8.3 Commercial Agent: Carboplatin Availability 68

9 BIOMARKER, CORRELATIVE, AND SPECIAL STUDIES 70
9.1 Integral Laboratory or Imaging Studies: N/A 70
9.2 Investigational Device Information: N/A 70
9.3 Integrated Correlative Studies: N/A 70
9.4 Exploratory/Ancillary Correlative Studies (Portions dependent on securing adequate funding). 70
9.5 Special Studies: N/A 78

10 STUDY CALENDAR 79

11 MEASUREMENT OF EFFECT 83
11.1 Antitumor Effect – Solid Tumors 83
11.2 Antitumor Effect – Hematologic Tumors- N/A 89
11.3 Other Response Parameters-N/A 89

12 STUDY OVERSIGHT AND DATA REPORTING / REGULATORY REQUIREMENTS 90
12.1 Data Reporting 90
12.2 CTEP Multicenter Guidelines: N/A 92
12.3 Collaborative Agreements Language 92

13 STATISTICAL CONSIDERATIONS 95
13.1 Study Design/Endpoints 95
13.2 Sample Size/Accrual Rate 96
13.3 Stratification Factors-N/A 97
13.4 Analysis of Secondary Endpoints 97

14 STUDY STATUS UPDATES AND STUDY CLOSURE 99
14.1 Definitions of Study Status Changes 99
14.2 Responsibility for Filing Protocol Status Update Forms 100

REFERENCES 101

APPENDIX A PERFORMANCE STATUS CRITERIA 107

APPENDIX B Talazoparib (BMN 673) MEDICATION DIARY 108

APPENDIX C: LIST OF CONTRAINDICATED MEDICATIONS* 119

APPENDIX D: INFORMATION ON POSSIBLE DRUG INTERACTIONS 120

1. OBJECTIVES

1.1 Primary Objectives

1.1.1 To determine the maximum tolerated dose (MTD) and recommended phase 2 dose (RP2D) of Talazoparib (BMN 673) seven day schedule in combination with carboplatin and paclitaxel

1.1.2 To determine the maximum tolerated dose (MTD) and recommended phase 2 dose (RP2D) of Talazoparib (BMN 673) three day schedule in combination with carboplatin and paclitaxel

1.2 Secondary Objectives

1.2.1 To observe and record anti-tumor activity of Talazoparib (BMN 673) in combination with carboplatin and paclitaxel. Although the clinical benefit of these drugs in combination has not yet been established, the intent of offering this treatment is to provide a possible therapeutic benefit, and thus the patient will be carefully monitored for tumor response and symptom relief in addition to safety and tolerability.

1.2.2 To determine whether the pharmacokinetic parameters of Talazoparib (BMN 673) when given in combination with carboplatin and paclitaxel correlate with thrombocytopenia

1.2.3 To observe and record anti-tumor activity of Talazoparib (BMN 673) alone after the combination with carboplatin, paclitaxel and Talazoparib (BMN 673).

1.2.4 To observe the safety and tolerability of Talazoparib (BMN 673) in combination with paclitaxel and carboplatin and Talazoparib (BMN 673) alone after the combination therapy.

1.3 Exploratory Objectives

1.3.1 To serially evaluate pharmacokinetic and pharmacodynamics parameters and use indirect pharmacokinetic/pharmacodynamics models to correlate with tumor response and resistance to the combination Talazoparib (BMN 673), carboplatin, and paclitaxel therapy.

1.3.2 To explore mechanisms of resistance to the combination of Talazoparib (BMN 673) with carboplatin and paclitaxel.

2 BACKGROUND

2.1 Advanced Solid Tumor in Adults

Cancer is the second leading cause of death in the United States.[4] The prognosis associated with distant metastases from adult solid tumors is poor for most malignancies. The goal of treatment with systemic therapies, either chemotherapy and/or biologic agents, is primarily palliative for these patients. There is a need to develop more efficacious and less toxic therapies for the treatment of cancer.

2.2 Talazoparib (BMN 673)

Talazoparib (BMN 673) is an orally available inhibitor of poly (ADP-ribose) polymerase (PARP), with an approved generic name of talazoparib. Talazoparib (BMN 673) is a novel, high purity, single enantiomer, methylbenzene sulfonate compound. It is the most potent and specific inhibitor of PARP1/2 in clinical development (IC50<1nM) and designed to have an improved therapeutic index relative to existing PARP inhibitors in development.

Mechanism of Action

Poly ADP-ribose polymerase (PARP) represents a family of at least 17 enzymes, at least two of which (PARP1 and PARP2) play important roles in DNA. [5-7] PARP senses and signals the presence of DNA damage and facilitates DNA repair. The polymerase catalyzes the addition of ADP-ribose units to histones, and various other proteins. PARP binds to single stranded DNA breaks (SSBs), cleaves NAD+, and attaches multiple ADP-ribose units to the target protein. [6, 8, 9] This results in a highly negatively charged target protein, and affects its function by leading to repair of the damaged DNA through the base excision repair (BER) pathway. PARP-1 and PARP-2 are nuclear proteins and are the only members of the PARP family with zinc-finger DNA binding domains. These domains localize PARP-1 and PARP-2 to the site of DNA damage. PARP-1 is highly conserved and has three structural domains (N-terminal DNA-binding domain; automodification domain, and the NAD^+-binding domain). The catalytic domain is located at the C-terminus end of the protein. In knockout mouse models, deletion of PARP-1 is sufficient to impair DNA repair. [10-12] The residual PARP-dependent repair activity (~ 10%) is due to PARP-2. This suggests that only PARP-1 and PARP-2 need to be inhibited to impair DNA repair. [6, 8, 13]

Increased PARP activity is one of the mechanisms by which tumor cells avoid apoptosis caused by DNA damaging agents. PARP activity is essential for the repair of single-stranded DNA breaks through the base excision repair (BER) pathways. [6, 9, 14-16] Therefore, inhibition of PARP sensitizes tumor cells to cytotoxic agents (e.g. alkylators [temozolomide, cyclophosphamide, BCNU and carboplatin] and topoisomerase I inhibitors [irinotecan, camptothecin, topotecan]) which induce DNA damage that would normally be repaired through the BER system. A significant therapeutic window appears to exist between a PARP inhibitor's ability to potentiate therapeutic benefit versus potentiation of undesirable side effects. As expected, PARP inhibitors do not potentiate agents that do not cause DNA damage.

PARylation has been implicated in many cellular processes including replication, transcription,

differentiation, gene regulation, protein degradation, and spindle maintenance. Enhanced PARP-1 expression and/or activity in tumor cells, as compared to normal cells, has been demonstrated in malignant lymphomas[17], hepatocellular carcinoma[18], cervical carcinoma[19], colorectal carcinoma[20], and non- Hodgkin's lymphoma.[21]

Multiple proteins are involved in the repair of DNA double strand breaks, and BRCA1 and BRCA2 are key components of this sensing pathway.[22] Synthetic lethality induced by PARP inhibition was first shown in vitro in tumor cells bearing mutations in the genes encoding

BRCA1 and BRCA2.[20,21] When PARP-1 is inhibited, SSBs persist and result in stalled replication forks and DSBs. In BRCA1- and BRCA2-deficient cells the homologous recombination (HR) pathway is faulty, and treatment with a PARP inhibitor results in cell cycle arrest and apoptosis.[23-25] The effect of PARP inhibitors in such cells with defects in the HR pathway is known as synthetic lethality.[26]

Clinical studies showed selective responses to PARP inhibitor therapy in BRCA1 and BRCA 2 mutation carrier patients with ovarian and breast cancers.[27-30] These studies have established the clinical relevance of PARP inhibitor-induced synthetic lethality in cancer patients whose tumors have BRCA mutations. Mutations or epigenetic changes to other proteins involved in the DNA double strand break pathway or changes to other DNA repair pathways might also be hypothesized to confer sensitivity to a PARP inhibitor. In particular dysfunction of genes involved in HR (e.g., Rad 51, PTEN, Fanconi Anemia, MRE11, ATM), may confer a similar sensitivity to PARP inhibition. [31-36]

Through proteomics screening, Byers et al identified high levels of PARP1 expression in both small cell lung cancer (SCLC) cell lines and patient tumor biopsies. [37] SCLC cell line proliferation was inhibited by PARP inhibition at concentrations similar to BRCA mutant cancer cell lines.

A comprehensive review of Talazoparib (BMN 673) can be found in the Talazoparib (BMN 673) Investigator's Brochure (2014). [38]

Nonclinical Activity

Talazoparib (BMN 673) is a highly potent and specific inhibitor of poly(ADP-ribose) polymerase (PARP) 1 and 2 with activity in tumor cell lines bearing DNA repair deficiencies.[33] Talazoparib (BMN 673) inhibits PARP *in vitro* at a lower concentration (IC_{50}= 0.57 nM) than ABT 888 (IC_{50}=4.73 nM), AG14447 (IC_{50}=1.98 nM), or olaparib (IC_{50}=1.94 nM). Talazoparib (BMN 673) also exerted single-agent synthetic lethality of BRCA 1 and 2 and PTEN deficient cell lines. In BRCA2 negative Capan 1- cells, Talazoparib (BMN 673) was more potent as single agent than ABT-888 (10,000 times), AG14447 (609 times) and olaparib (259 times) in inhibiting PARP activity.

Assessment of Talazoparib (BMN 673) and the related compounds for their tumor cell cytotoxicity revealed selective and potent cytotoxicity in human cancer cell lines harboring mutations that compromise DNA repair pathways. Gene mutations that confer selective tumor

cell cytotoxicity included BRCA1 (MX-1 mammary tumor cells), BRCA2 (Capan-1 pancreatic tumor cells), PTEN (MDA- MB-468 mammary, LNCap and PC-3 prostate tumor cells), and MLH-1 mutations (HCT-116 colorectal tumor cells). The IC_{50} values of Talazoparib (BMN 673) in these tumor cell lines were in the single digit nanomolar or sub-nanomolar range. In contrast, the IC_{50} of Talazoparib (BMN 673) against normal human primary cell MRC-5 and several tumor cell lines that do not have reported DNA repair- related mutations are significantly greater (250 nM to >1000 nM).

In the BRCA1-deficient MX-1 xenograft tumor model, oral administration of 0.33 mg/kg Talazoparib (BMN 673) once daily for 28 days resulted in significant antitumor activity (tumor growth delay/tumor regression).Dose-related inhibition of tumor growth was observed at lower doses, while a higher dose (1.0 mg/kg/day) of Talazoparib (BMN 673) induced significant body weight loss with associated mortality. Consistent with the anti-tumor effect in this model, profound reduction in poly (ADP ribose) (PAR) levels (the product of PARP1/2 and therefore a measure of PARP activity), was observed in MX-1 xenografts when mice were treated with Talazoparib (BMN 673) orally. Oral administration of Talazoparib (BMN 673) (0.33 mg/kg/day for 28 days) also demonstrated anti-tumor activity in the PTEN- deficient LNCap, MDA-MB-468, and PC-3 mouse xenograft models. In addition, twice-a-day oral administration of Talazoparib (BMN 673) (0.33 mg/kg/day for 28 days) delayed the growth of the MLH- 1–deficient HCT-116 xenograft tumor in nude mice. No tumor growth delay of NCL-H69 human SCLC or HCI-H82 human lung xenografts was observed in animals given 0.1 mg/kg/day. During the conduct of these two non-GLP primary pharmacodynamic studies, mortalities in the mouse xenograft model occurred. The incidence of these mortalities and body weight loss was generally dose related and was observed across multiple mouse xenograft studies.

Assessment of various dosing schedules in the mouse xenograft models indicated that continuous daily administration of Talazoparib (BMN 673) resulted in greater antitumor activity than intermittent administration at the same or higher dose levels. Twice daily (BID) dosing resulted in extended tumor growth delay or extended tumor regression compared to single daily (QD) dosing at an identical total daily dose level for 28-days. These results suggest that continuous suppression of PARP activity is required for optimal anti-tumor activity. Optimal dosing schedules are expected to be species specific. Interspecies scaling of $t_{1/2}$ in rats and dogs predict a human $t_{1/2}$ that is sufficient to support a regimen of once daily administration.

To determine possible organ systems that may be impacted by inhibition of PARP activity, SD rats were administered Talazoparib (BMN 673) and various tissues were analyzed for PARP activity. Profound reductions in PAR levels were observed in various tissues, most notably in bone marrow, which has one of the highest baseline levels of PAR (weight-normalized) among all rat tissues analyzed. This is consistent with the toxicology studies, which demonstrated that the hematopoietic system is a target of Talazoparib (BMN 673) activity.

Six non-GLP primary PD studies in the mouse xenograft model were conducted. One study examined Talazoparib (BMN 673) monotherapy in Ewing’s Sarcoma and osteosarcoma cell lines (Report No. BMN673-12-016). Five studies were combination therapy studies examining the drug pharmacodynamic relationship of Talazoparib (BMN 673) with temozolomide in small cell lung cancer, melanoma, Ewing sarcoma, Wilms tumor, glioblastoma and rhabdomyosarcoma cell lines in the mouse xenoblastoma (Report Nos.BMN673-13-033,

BMN673-13-034, BMN673-13-054, BMN673-13-063 and BMN673-13-066). One of the five studies examining the drug pharmacodynamic interactions included an in vitro NCI cell line panel study phase assessing childhood solid and leukemia cell lines (BMN 673-13-063).

No mortalities were observed in the mouse xenograft study assessing Talazoparib (BMN 673) monotherapy in the mouse xenograft model of Ewing's sarcoma (TC106, CHP100S and CHLA9) and osteosarcoma (KHOS) (Report No. BMN673-12-016).

Talazoparib (BMN 673) and temozolomide (TMZ) PD interactions in the mouse xenograft model with human small cell lung cancer cell lines (NCI-H1048 and CNI-H841) were assessed in three studies looking at maximum tolerated dose, dose regimen and schedule over multiple phases (Report Nos.BMN673-13-033, BMN673-13-034, BMN673-13-054). 24 mortalities occurred during this assessment due to body weight loss that has been observed previously in mouse xenograft studies.

Twelve mortalities were observed in the assessment of melanoma cell line (M207, M32 and M249) after treatment with Talazoparib (BMN 673) and TMZ due to severe body weight loss (BMN673- 13-066. 115 mortalities were observed during multiple phases assessing PD interactions of Talazoparib (BMN 673) and TMZ in SCID, nude and NOD SCID mouse xenograft models assessing a panel of childhood solid/leukemia cell lines including Ewing's sarcoma, Wilms tumor, glioblastoma, rhabdomyosarcoma, neuroblastoma and osteosarcoma (Report No. BMN673-13-063). Mouse strain specific toxicity was observed at an increased incidence in the NOD-SCID mouse (50%) and the nude mouse model (37%).

Overall, these mortalities have a relationship to body weight loss and appear to be similar to those observed previously in xenograft studies. Mortalities also appeared to have a relationship to the different mouse strains utilized in these studies and dose of both Talazoparib (BMN 673) and TMZ. Because these mouse strains (NOD-SCID and nude mouse) were not utilized previously, this observation is new but has no impact on the safety profile.

Nonclinical Pharmacology and Toxicology

Talazoparib (BMN 673) was assessed for effects on poly (ADP-ribose) glycohydrolase (PARG), receptor/ion channel & enzyme activity, CYP450, hERG cell current and in AMES tests, in vitro. Safety pharmacology parameters of the respiratory, central nervous system (CNS) and cardiovascular systems were assessed in vivo. No significant undesired effects were observed in these studies. In *in vitro* studies, Talazoparib (BMN 673) did not inhibit any of the 5 major CYP450 isozymes (1A2, 2C19, 2D6, 2C9 and 3A4).

The oral bioavailability, calculated from the ratio of area under the concentration-time curve (AUC) following oral administration relative to the AUC following intravenous (IV) administration (AUCoral/AUCIV), was > 42.7% in rats and > 50.5% in dogs based on single dose comparisons. The compound was metabolically stable. The mean terminal half-life ($t_{1/2}$) of Talazoparib (BMN 673) at various doses in rats and dogs ranged from 20.5 to 51.5 hours and 60.9 to 91.2 hours, respectively, which allows for once daily dosing.

Pharmacokinetic studies have been performed in rats and dogs. Steady state concentrations were reached by Day 15 in rats and by Day 20 in dogs using once daily administration of Talazoparib

(BMN 673). Comparing Day 15 and 28 with Day 1 for all dose levels in dogs, AUC and Cmax increased from Day 1 to Day 15 to Day 28.

Five day repeat dose toxicity and toxicokinetic (TK) studies with 28-day recovery were conducted in rats and dogs. In dogs (the most sensitive species), Talazoparib (BMN 673) was administered at dose levels of 0.003, 0.01, 0.03, 0.1 mg/kg/day over 5 consecutive days. Severe pancytopenia was observed in dogs treated with the two highest dose groups (0.03 and 0.1 mg/kg/day). At these doses, the mean (or median) reticulocyte nadir occurred on day 6 and the platelet and WBC nadirs on day 11. These changes were reversed in the group treated at 0.03 mg/kg/day on days 17-18 (i.e., 12-13 days after the last dose of the drug). Mortalities occurred in animals given 0.1 mg/kg/day due to bacterial septicemia secondary to bone marrow hypocellularity and lymphoid organ depletion on day 12-13. Coagulation parameters were unaffected. After repeat-dose administration of daily oral Talazoparib (BMN 673) in dogs for 5-days, the highest non-severely toxic dose (HNSTD) was 0.03 mg/kg/day.

Twenty-eight day repeat dose toxicity and TK studies with 28-day recovery were also conducted in rats and dogs. In dogs (the most sensitive species), Talazoparib (BMN 673) was administered at dosage levels of 0.0005, 0.0015, 0.005, 0.01 mg/kg/day over 28 consecutive days. Talazoparib (BMN 673)-related signs included hematology findings in males and females given 0.005 or 0.01 mg/kg/day such as mildly lower red cell mass, mildly to moderately lower platelet and absolute reticulocyte counts, and minimally to mildly lower white blood cell counts with a generalized decrease in all leukocytes. All of these signs reversed or were reversing by the end of the recovery phase. After repeat-dose administration of daily oral Talazoparib (BMN 673) in dog for 28 days, the HNSTD was 0.01 mg/kg/day.

13-week repeat dose toxicity and TK studies with 28-day recovery were also conducted in rats and dogs. In dogs, Talazoparib (BMN 673) was administered at dosage levels 0.0015, 0.005 and 0.01 mg/kg/day. Talazoparib (BMN 673)-related signs included hematology findings in males and females given 0.01 mg/kg/day such as mildly to moderately lower red cell mass, platelet counts and absolute reticulocyte counts and minimally to mildly lower white blood cell counts. All of hematology findings related to bone marrow depletion were reversed by the end of the recovery phase. After repeat-dose administration of daily oral Talazoparib (BMN 673) in dog for 28 days, the HNSTD was 0.01 mg/kg/day.

In conclusion, the main nonclinical findings of early hematological changes, and subsequent bone marrow and lymphoid organ depletion as well as focal necrosis after repeat administration of Talazoparib (BMN 673) are in accordance with the mechanism of action and the exposure/distribution pattern. These findings were reversible and the decreased reticulocyte, platelet, red blood cell (RBC) and WBC counts were sensitive and early markers of target organ toxicity. Decreases in hematology parameters were used to clinically monitor safety. The HNSTD based on data from the 5-day and 28-day toxicity and TK studies in rat and dog are presented in Table 1. A starting clinical dose was estimated using the dog HNSTD as the dog was more sensitive to Talazoparib (BMN 673)- related primary toxicities than the rat. Based on a 6 times safety factor relative to the 28-day toxicity study in dog HNSTD, the estimated safe clinical starting dose is 1 mcg/kg/day.

Table 1.

Study Treatment Schedule	Species	HNSTD[a] (mg/kg)	HED[b] (mg/kg)	Estimated Safe Clinical Starting Dose[c] (mcg/kg)	Recalculated Estimated Safe Clinical Starting Dose[d] (mcg/kg)
5-day/28-day recovery	Rat	0.3	0.0484	5	2.5
	Dog	0.03	0.0167	3	1.5
28-day/28-day recovery	Rat	0.05	0.0081	1	0.5
	Dog	0.01	0.0056	1	0.5

[a] Highest non-severely toxic dose (HNSTD)
[b] Human equivalent dose based on body surface area (HED) for a 60kg human
[c] Clinical starting dose was based on the following conversion: Rodent 1/10th LD_{10};
Nonrodent: 1/6 [th] HNSTD
[d] Clinical starting dose was recalculated based on the 2-fold increase of relative bioavailability of the capsule presentation to be used in clinic

The difference in relative bioavailability after administration of the capsule and oral gavage suspension of Talazoparib (BMN 673) was approximately two-fold. Taking into account the relative change in exposure with capsule versus the oral gavage suspension, the estimated safe clinical starting dose was recalculated and is 0.5 mcg/kg/day Talazoparib (BMN 673). Therefore, the starting fixed clinical dose of 25 mcg Talazoparib (BMN 673) in a capsule form corresponds to 0.42 mcg/kg/day for a 60 kg adult.

Clinical Investigations

In a phase I study of Talazoparib (BMN 673)[39], the pharmacokinetics (PK), pharmacodynamics (PD), safety and anti-tumor activity of Talazoparib (BMN 673) were evaluated in a 2-stage dose-escalation study with 3-6 patients/dose level. In dose escalation (Stage 1) cycle 1 was 6 weeks, with drug taken on days 1 and 8-35, for PK and PD assays, followed by daily continuous dosing in 4-wk cycles. Stage 2 (expansion at MTD) recruits patients with tumors defective in DNA repair (Ewing sarcoma, small cell lung cancer or tumors associated with BRCA mutation). Thirty-nine patients (33F/6M) were enrolled in 9 cohorts from 25 to 1100 mcg/d that defined a MTD of 1000 mcg/d. Tumors (# with deleterious BRCA 1/2 mutation) included 23 ovarian/primary peritoneal (17); 8 breast (6); 3 pancreas; 2 colon; 1 prostate (1), and 1 mullerian carcinosarcoma. 17 and 8 pts had BRCA 1 and 2 mutations, respectively. Dose-limiting thrombocytopenia occurred in 1/6 and 2/5 pts at 900 and 1100 mcg/d, respectively. Potentially-related adverse events in >10% of pts (# grade 1 and 2/grade 3 and 4) included fatigue (10/0); nausea (10/0); flatulence (4/0); anemia (5/2); neutropenia (4/3); thrombocytopenia (1/3); and grade 1 alopecia (10). Inhibition of PARP activity in peripheral blood mononuclear cells (PBMCs) was observed at doses ≥ 100 mcg/d. Talazoparib (BMN 673) plasma concentrations peaked 1-2 hours post-dose; exposure increased dose proportionally. Steady state plasma concentrations were reached by the end of the 2nd week of daily dosing; mean Cmax: 0.30 - 25.4 ng/mL and AUC0-24: 3.96 - 203 ng-hr/mL across the 25 to 1100 mcg/d dose range after 28d of daily dosing. RECIST and/or CA-125 responses occurred at

doses ≥ 100 mcg/d in 11/17 BRCA carrier ovarian/peritoneal cancer patients. Objective responses occurred in 2/6 BRCA-carrier breast cancer patients. Talazoparib (BMN 673) was well tolerated with impressive anti-tumor activity in patients with BRCA mutation with a single agent recommended Phase II trial dose of 1000 mcg/d due to dose-limiting thrombocytopenia.

Additional early phase clinical studies of Talazoparib (BMN 673) are ongoing in different tumor types and schedules. There is a two-arm, open-label study to assess the safety, PK, PD, and preliminary efficacy of Talazoparib (BMN 673) in patients with advanced hematological malignancies: Arm 1 Acute myeloid leukemia (AML), Myelodysplastic Syndrome (MDS); Arm 2 Chronic Lymphocytic Leukemia (CLL), Mantle Cell Lymphoma (MCL). The initial cohort of patients for each arm was treated with 100 mcg Talazoparib (BMN 673) once daily. As of 30 November 2013, a total of 33 patients had been enrolled in the study (which has been closed for enrollment). Results are not yet reported.

Another ongoing study is an open-label, randomized, parallel, 2-arm study of Talazoparib (BMN 673) versus protocol-specific physician's choice therapy in subjects with germline BRCA mutation locally advanced and/or metastatic breast cancer. As of 30 November 2013, a total of 3 subjects had been enrolled in the study.

The effect of food on the oral bioavailability of Talazoparib (BMN 673) was evaluated in 18 healthy male subjects who received a single 500-mcg dose of Talazoparib (BMN 673) following an overnight fast and following a high-fat, high-calorie meal in a standard 2x2 crossover design. Food delayed the absorption Talazoparib (BMN 673), as indicated by a prolonged Tmax and reduced Cmax under fed compared to fasted conditions, but did not affect the overall extent of absorption, as indicated by similar AUC0-t and AUC0-∞ values under fed and fasted conditions. Single 500 mcg doses were safe and well tolerated in healthy male volunteers.

Cumulatively, there have been 111 SAEs reported in clinical studies with Talazoparib (BMN 673) as of 30 November 2013. The most common SOCs for SAEs have been infections and infestations (33 events), gastrointestinal disorders (16 events), blood and lymphatic disorders (13 events), and respiratory, thoracic, and mediastinal disorders (12 events). The most commonly reported SAEs by preferred term include febrile neutropenia (9 events), neutropenic sepsis (7 events), ascites (5 events), and bacteremia (5 events). All but 9 of these 111 SAEs were assessed by the investigators as not related to treatment with Talazoparib (BMN 673).

Adverse events occurring in at least 10% of patients in the pooled study population have included: fatigue, nausea, anemia, constipation, diarrhea, vomiting, thrombocytopenia, cough, pyrexia, headache, neutropenia, alopecia, decreased appetite, abdominal pain, back pain, dyspnea, pain in extremity, hypokalemia, anxiety, arthralgia, and dizziness. Adverse reactions have not worsened with continued therapy at the same or reduced dose.

2.3 Carboplatin and Paclitaxel Combination

Carboplatin is a commonly used platinum alkylating compound that acts by binding to DNA and interrupting cell division. It is approved by the FDA for the treatment of patients with ovarian cancer. It is also used for the treatment of non-small cell lung cancer, small cell lung cancer, head

and neck cancer, endometrial cancer, metastatic seminoma and more recently in triple negative breast cancer. Carboplatin is eliminated by renal excretion. The clearance is related to the glomerular filtration rate (GFR). Therefore it is dosed based on the GFR and the target area under the concentration versus time curve (AUC). The main side effect of carboplatin is myelosuppression. Other toxicities include nausea, vomiting, renal and neurotoxicity. Carboplatin does not inhibit, induce and is not a substrate for any CYP450 isozymes.

Paclitaxel is a novel antimicrobial agent that promotes the assembly of microtubule formation and stabilizes them by preventing depolymerization. It is derived from the needles and bark of the pacific yew tree. It is insoluble in water and is therefore formulated in Cremophor®. It is approved by the FDA for the treatment of ovarian cancer, breast cancer, non-small cell lung cancer and Kaposi's sarcoma. It is also in use for the treatment of several other solid malignancies. It is administered as an IV infusion. It can be used either on the 3-weekly schedule or on a weekly schedule. Main toxicities associated with the use of paclitaxel include myelosuppression, neuropathy and hypersensitivity reactions. Paclitaxel is classified as a CYP2C8 sensitive substrate and CYP3A4 sensitive substrate and moderate inducer.

The combination of paclitaxel and carboplatin is widely used for the treatment of patients with breast cancer, advanced NSCLC, ovarian cancer, bladder cancer and other solid tumors.

Administration of carboplatin in combination with paclitaxel results in less thrombocytopenia than is expected from the use of carboplatin alone. [40] In several recent studies, the combination of paclitaxel and carboplatin has shown similar efficacy compared to paclitaxel/cisplatin, vinorelbine/cisplatin, cisplatin/ gemcitabine and cisplatin/docetaxel but is associated with an overall reduction in toxicity for patients with advanced non-small cell lung cancer (NSCLC).[41 42] Belani and colleagues compared the efficacy of the carboplatin-paclitaxel combination with cisplatin-etoposide for patients with advanced NSCLC.[43] The two regimens had comparable efficacy in this randomized trial, although the quality of life was more favorable for the carboplatin-paclitaxel arm. The Eastern Cooperative Oncology Group (ECOG) 1594 trial compared three different regimens (cisplatin-docetaxel, cisplatin-gemcitabine, carboplatin-paclitaxel) against the control arm of cisplatin- paclitaxel for patients with advanced NSCLC.[41] Over 1100 patients with advanced NSCLC were accrued to this trial. While the toxicity profiles varied between the chemotherapy regimens, there were no significant differences in most of the efficacy endpoints analyzed, including overall survival, 1-year survival, response rates, and median survival. Kelly et al reported the results of the Southwest Oncology Group (SWOG) 9509 trial that compared the combination of carboplatin-paclitaxel with their prevailing standard regimen of cisplatin-vinorelbine. [44] Again there were no differences in the efficacy parameters between the two treatment arms. The experimental arm of carboplatin- paclitaxel was associated with a lower incidence of nausea and vomiting and a higher incidence of neurotoxicity. Fewer patients in the carboplatin-paclitaxel arm discontinued treatment due to toxicity.

In patients with advanced ovarian cancer, the combination of carboplatin and paclitaxel was compared with cisplatin-paclitaxel. The carboplatin-paclitaxel regimen was associated with less toxicity, easier administration and better tolerated. This study led to the adoption of paclitaxel-carboplatin as the preferred combination for the treatment of advanced ovarian carcinoma.[45] The current standard of care for patients with ovarian carcinoma is 6 cycles of paclitaxel dosed at 175 mg/m^2 plus carboplatin dosed to target an AUC of 5.0 to 7.5.[45]

The carboplatin-paclitaxel combination is also effective in the treatment of head and neck cancer[46], esophageal cancer[47], breast cancer[48] and bladder cancer.[49] The wide spectrum of anti-tumor activity noted with the combination of carboplatin and paclitaxel makes this an ideal platform for the evaluation with various novel agents.

The recommended dose of carboplatin for this combination is AUC 5-6 mg/ml.min and the dose for paclitaxel ranges from 175mg/m^2 to 225 mg/m^2 (administered every 3 weeks). However, recent studies of neoadjuvant paclitaxel and carboplatin in triple negative breast cancer[48,49] and Phase III studies in advanced ovarian and non-small cell lung cancer have been using weekly dosing of paclitaxel at 80mg/m^2.[50-53] This regimen has been better tolerated with comparable efficacy.

2.4 Rationale for Combination

Carboplatin is a platinum analogue chemotherapeutic agent that has been in use since the 1980s. Platinums function as alkylating agents by binding to cellular DNA to form cross links. The resulting DNA damage from carboplatin has been shown to be associated with activation of PARP.[54, 55] PARP inhibition potentiates the anti-tumor effect of these DNA damaging chemotherapies, including platinums. [55, 56] As a single agent, carboplatin is associated with bone marrow suppression, particularly thrombocytopenia. It can also cause fatigue, nausea, and renal and neurotoxicity. Carboplatin is currently used in the treatment of many solid tumors, including ovarian cancer, non-small cell lung cancer, germ cell tumors, bladder cancer, upper gastrointestinal malignancies, and breast cancer. For the majority of these tumor types it is combined with paclitaxel.

Paclitaxel is an antimitotic cytotoxic agent. Although synergy has not been demonstrated with paclitaxel and PARP inhibitors, the non-overlapping toxicities with carboplatin and Talazoparib (BMN 673) (low rates of severe thrombocytopenia with paclitaxel) make the paclitaxel and carboplatin combination appealing to study with Talazoparib (BMN 673). Notably, optimal schedules of paclitaxel and carboplatin vary by tumor type. Most commonly, carboplatin is dosed every 21 days at either AUC 5-6 and paclitaxel is either dosed every 21 days at 175-225 mg/m^2. However, recent studies of neoadjuvant paclitaxel and carboplatin in triple negative breast cancer[48,49] and Phase III studies in advanced ovarian and non-small cell lung cancer have been using weekly dosing of paclitaxel at 80mg/m^2.[50-53] Since the plan for development of this combination will include both breast and ovarian cancer, we plan to investigate the combination of Talazoparib (BMN 673) with carboplatin every 3 weeks and weekly paclitaxel in this phase I study.

Preclinical data support combinations of PARP inhibitors with platinum chemotherapies.[52,53] In the presence of chemotherapy, it is not clear if dose or duration of PARP inhibition is more important[1-3]. Additionally, in the phase I study Talazoparib (BMN 673) dosed continuously, myelosuppression was primary DLT, in particular grade 3 and 4 thrombocytopenia. This remains a significant concern when combining with chemotherapy. Thus, this study is evaluating the intermittent schedule of Talazoparib (BMN 673) in combination with carboplatin and weekly paclitaxel. At any time after 4-6 cycles of the combination therapy, for patients with clinical benefit defined as response or stable disease, extension of therapy is allowed with a)

carboplatin, paclitaxel and Talazoparib (BMN 673) b) carboplatin and Talazoparib (BMN 673) without paclitaxel c) Talazoparib (BMN 673) daily continuous dosing or d) observation without therapy per discretion of the treating provider. If the patient is continued on a) or b), subsequent change to c) at later timepoints is also allowed.

Rationale for starting dose for schedule B. In Schedule A, 14 total patients have started treatment at dose level 2 (carboplatin AUC 6, paclitaxel 80mg/m^2 and Talazoparib (BMN 673) 250mcg). Two were not evaluable in cycle 1 for DLT. Of the 12 evaluable patients, two experienced DLT (both with grade 3/4 neutropenia lasting > 7 days), 9 completed cycle 1 without DLT and 1 patient is currently in cycle 1. Thus, no more than 3 patients will experience DLT at DL2 for schedule A. This meets the protocol criteria for MTD for schedule A being defined as dose level 2 which will be the starting dose level for schedule B.

2.5 Correlative Studies Background

2.5.1 Pharmacokinetics

Based on the pharmacokinetic parameters of the 3 study drugs (carboplatin, paclitaxel and Talazoparib (BMN 673)), drug-drug interactions are not anticipated and therefore, limited pharmacokinetic analysis is proposed. The plasma pharmacokinetics (Cmax) of Talazoparib (BMN 673) will be evaluated as a potential marker of toxicity, specifically thrombocytopenia. If funding is secured, the study will also explore correlating PK with PD changes observed in PBMCs using an indirect PK/PD model.

2.5.2 Pharmacodynamic Studies and Mechanisms of Resistance-(Dependent on securing additional funding. See section 9.0 for details.)

Rationale for exploratory studies: Cells are continuously exposed to DNA damage and have multiple mechanisms for DNA repair. Repair of DNA damage restores genomic integrity and is important for cell survival. The PARP1 and 2 enzymes are involved in base excision repair of single-strand breaks. When PARP enzymes are inhibited, these single strand breaks are converted to double strand breaks.[50, 54] Unless repaired by the homologous recombination pathway, which includes BRCA 1 or 2, this DNA damage ultimately leads to cell death.[23] Notably, platinums bind to cellular DNA to form cross links which result in DNA strand breaks. The resulting DNA damage from carboplatin has been shown to be associated with activation of PARP.[54, 55] Preclinical data demonstrates that PARP inhibition potentiates the anti-tumor effect of platinums.[55, 57]

Measurements of DNA damage and PARP inhibition can be used to evaluate the effect of combination therapy with platinums and PARP inhibitors. Activation of DNA-damage signaling pathways occurs through phosphorylation of histone H2AX (γH2AX).[58] RAD51 is involved in homologous recombination repair. In response to DNA damage, RAD51 is relocalized within the cell nucleus to form distinct foci which can be visualized.[59, 60] The product of PARP 1 and 2 enzyme activity, PAR, has been utilized in previous studies as a measure of PARP activity.[61] These assays have been performed previously in PBMCs and circulating tumor cells (CTC)s.

Many questions about this combination remain. There is still limited data about the optimal duration or scheduling of PARP inhibitors with platinums as well as mechanisms of resistance to the platinum and PARP inhibitor combination. The exploratory correlative studies proposed will examine these questions.

Clinical trials with PARP inhibitors have investigated various schedules when combined with chemotherapy. These include continuous dosing, intermittent dosing with PARP inhibitors staring prior to the chemotherapy administration and intermittent dosing starting on the same day as chemotherapy. [62-64] None of these studies have defined the optimal schedule for these combinations. In this phase I trial, Talazoparib (BMN 673) is being dosed on either a 3 day or 7 day schedule starting with every 3 week carboplatin dose. Previous pharmacodynamics studies indicate that PARP activity was rapidly inhibited in PBMCs with Talazoparib (BMN 673).[65] This has also been demonstrated with other PARP inhibitors. [66] We will use validated assays examining induction of γH2AX and presence of RAD51 foci, and PAR inhibition both in PBMCs. These tests will assay for DNA damage after the 3 or 7 day course of Talazoparib (BMN 673).[58] All patients on study will have PBMCs collected at the serial time points described in section 9 of the protocol.

The mechanisms by which tumor cells develop resistance to PARP inhibitors are not well understood. In BRCA1 mutant cancers, resistance to PARP inhibitors can arise by loss of 53BP1, a key regulator of DNA repair choice. [67-70] Similarly, loss of some critical partners of 53BP1, such as PTIP and RIF1, can also lead to PARP inhibitor resistance in BRCA1 mutant cells.[71, 72] An alternative mechanism of resistance to PARP inhibition in both BRCA1 and BRCA2 mutant cancers are selection for reversion mutations that at least partially restore BRCA1 or BRCA2 function.[73, 74] The mechanisms underlying resistance in BRCA1/2 wild type tumors are even less well understood. To explore potential mechanisms of resistance we propose evaluation of tumor tissue and CTCs aimed at key components of the DNA repair pathways. All subjects will be asked to submit archived tumor tissue collected at baseline as described in section 9 of the protocol. An optional biopsy of tumor tissue will be obtained upon progression of disease in all patients with evidence of tumor response to the study combination therapy. For patients with measurable disease, RECIST 1.1 will be used to define response. For those with measurable disease, clinical response will be based on treating provider's interpretation using clinical data.

Baseline and progressive tumor samples will undergo genomic sequencing to compare for mechanisms of acquired resistance. Our hypothesis is that paired analysis of pharmacokinetic, pharmacodynamic, and genomic studies will reveal resistance mechanisms to this combination of Talazoparib (BMN 673) with carboplatin and paclitaxel. This data will be used to inform future studies of this combination.

3 PATIENT SELECTION

3.1 Eligibility Criteria

3.1.1 Patients must have histologically confirmed solid malignancy (excluding lymphoma) that is metastatic or unresectable and for which standard curative measures do not exist or are no longer effective, and for which:

a) there is reasonable expectation of response to the combination of carboplatin/paclitaxel OR

b) BRCA 1/2 germline mutation is present. Due to the longstanding acceptance of BRCA 1 and 2 mutation testing through Myriad, results from Myriad will be acceptable.
If testing for BRCA 1 and 2 germline mutations is done through another organization, a report from a genetics consult with a qualified medical professional confirming that the laboratory results show a recognized germline deleterious BRCA 1 or 2 mutation or rearrangement is required. If the latter cannot be obtained, PI or study chair review of the lab results and confirmation of BRCA mutation or rearrangement will be required OR

c) BRCA 1/2 somatic mutation previously identified using a CLIA certified assay.

3.1.2 Patients must have measurable or evaluable disease, as defined by RECIST 1.1.

3.1.3 Age ≥18 years. Because no dosing or adverse event data are currently available on the use of Talazoparib (BMN 673) in combination with carboplatin and paclitaxel in patients <18 years of age, children are excluded from this study, but will be eligible for future pediatric trials.

3.1.4 ECOG performance status ≤2 (Karnofsky ≥60%, see Appendix A).

3.1.5 Life expectancy of greater than 12 weeks.

3.1.6 Patients must have normal organ and marrow function as defined below:

– absolute neutrophil count	≥1,500/mcL
– hemoglobin	≥ 9 g/dL
– platelets	≥150,000/mcL
– total bilirubin	≤1.25 x institutional upper limit of normal (ULN), with the exception of <2.9 mg/dL for patients with Gilbert's disease
– AST(SGOT)/ALT(SGPT)	≤2.5x ULN); ≤ 5x ULN in setting of metastatic liver disease
– creatinine	≤1.5 x upper limit of normal OR
– creatinine clearance	≥50 mL/min

3.1.7 Ability to take oral medications

3.1.8 Patients with central nervous system (CNS) metastases must be stable after therapy for CNS metastases (such as surgery, radiotherapy or stereotactic radiosurgery) for at least 4 weeks and must be off steroid treatment for 2 weeks prior to study enrollment.

3.1.9 The effects of Talazoparib (BMN 673) on the developing human fetus are unknown. For this reason and because other therapeutic agents used in this trial are known to be teratogenic, women of child-bearing potential and men must agree to use adequate contraception (hormonal or barrier method of birth control; abstinence) prior to study entry and for the duration of study participation. Should a woman become pregnant or suspect she is pregnant while she or her partner is participating in this study, she should inform her treating physician immediately. Men treated or enrolled on this protocol must also agree to use adequate contraception prior to the study, for the duration of study participation, and 4 months after completion of Talazoparib (BMN 673) administration.

3.1.10 Ability to understand and the willingness to sign a written informed consent document.

3.2 Exclusion Criteria

3.2.1 Patients who have had chemotherapy or radiotherapy within 4 weeks (6 weeks for nitrosoureas or mitomycin C) or targeted therapies within 2 weeks prior to entering the study or those who have not recovered (≤ grade 1) from adverse events due to agents administered with the exception of any grade of alopecia.

3.2.2 No prior carboplatin unless given in neoadjuvant/adjuvant setting for curative intent and more than 6 months have elapsed since last carboplatin dose. In the case of relapsed ovarian cancer, patients are eligible if more than 6 months have elapsed since last carboplatin dose.

3.2.3 Patients who are receiving any other investigational agents.

3.2.4 History of allergic reactions attributed to compounds of similar chemical or biologic composition to Talazoparib (BMN 673) or other agents used in study.

3.2.5 Peripheral neuropathy of severity greater than grade 1.

3.2.6 The following medications are contraindicated (see list of medications in Appendix C) or must be used with caution. Because the lists of these agents are constantly changing, it is important to regularly consult a frequently-updated medical reference.

Contraindicated:

- CYP2C8 strong and moderate inhibitors

- CYP2C8 inducers
- CYP3A4 strong and moderate inhibitors
- CYP3A4 inducers
- CYP3A4 sensitive substrates

Exclusions: The following supportive care medications will be allowed as they are routinely administered with carboplatin and paclitaxel and have no potential interaction with Talazoparib (BMN 673): dexamethasone, aprepitant, fosaprepitant, and ondansetron). Oral pain medications such as hydrocodone, oxycodone taken on an as needed basis are also permitted.

Transdermal products designed for systemic delivery must be assessed for interaction potential. Topical products not designed to provide systemic delivery (including inhaled products, ophthalmologic products and transvaginal preparations) do not need to be considered since they do not have appreciable systemic absorption.

Other contraindicated medications (per above) are not allowed unless close monitoring with labs or drug levels or by symptoms with subsequent dose adjustments is feasible. Patients taking these concurrent medications are ineligible unless they can discontinue or switched to alternative medications prior to initiation of the study drug (at least 5 half-lives).

Use with Caution:

- CYP2C8 sensitive substrates
- CYP2C8 weak inhibitors
- CYP3A4 non-sensitive substrates
- CYP3A4 weak inhibitors

These agents may be permitted if discontinuation is not feasible and no acceptable alternatives are available as determined by the treating physician; however, caution should be used. Consider monitoring with labs or drug levels or by symptoms and consider dose adjustments of the medication.

3.2.7 Uncontrolled intercurrent illness including, but not limited to, ongoing or active infection, symptomatic congestive heart failure, unstable angina pectoris, cardiac arrhythmia, or psychiatric illness/social situations that would limit compliance with study requirements.

3.2.8 Pregnant women are excluded from this study because Talazoparib (BMN 673) *is* a PARP inhibitor with the potential for teratogenic or abortifacient effects. Because there is an unknown but potential risk for adverse events in nursing infants secondary to treatment of the mother with Talazoparib (BMN 673), breastfeeding should be discontinued if the mother is treated with Talazoparib (BMN 673). These potential risks may also apply to other agents used in this study.

3.2.9 HIV-positive patients on combination antiretroviral therapy are ineligible because of the potential for interactions with study treatment. In addition, these patients are at increased risk of lethal infections when treated with marrow-suppressive therapy. Appropriate studies will be undertaken in patients receiving combination antiretroviral therapy when indicated.

3.2.10 No clinically significant bleeding (i.e. GI bleed, intracranial bleeding) within 6 months or major surgery within 4 weeks. Minor surgeries (i.e. port placement, cataract surgery) are allowed within 2 weeks

3.2.11 Anticoagulation and anti-platelet therapies are not permitted (this includes Coumadin, low molecular weight heparins, factor Xa inhibitors, aspirin and NSAIDS or other medicines with similar effects).

3.3 Inclusion of Women and Minorities

Both men and women of all races and ethnic groups are eligible for this trial.

4 REGISTRATION PROCEDURES

4.1 Investigator and Research Associate Registration with CTEP

Food and Drug Administration (FDA) regulations and National Cancer Institute (NCI) policy require all individuals contributing to NCI-sponsored trials to register and to renew their registration annually. To register, all individuals must obtain a Cancer Therapy Evaluation Program (CTEP) Identity and Access Management (IAM) account (https://ctepcore.nci.nih.gov/iam). In addition, persons with a registration type of Investigator (IVR), Non-Physician Investigator (NPIVR), or Associate Plus (AP) (i.e., clinical site staff requiring write access to OPEN or RAVE or acting as a primary site contact) must complete their annual registration using CTEP's web-based Registration and Credential Repository (RCR) (https://ctepcore.nci.nih.gov/rcr). Documentation requirements per registration type are outlined in the table below.

Documentation Required	IVR	NPIVR	AP	A
FDA Form 1572	✔	✔		
Financial Disclosure Form	✔	✔	✔	
NCI Biosketch (education, training, employment, license, and certification)	✔	✔	✔	
HSP/GCP training	✔	✔	✔	
Agent Shipment Form (if applicable)	✔			
CV (optional)	✔	✔	✔	

An active CTEP-IAM user account and appropriate RCR registration is required to access all CTEP and CTSU (Cancer Trials Support Unit) websites and applications. In addition, IVRs and NPIVRs must list all clinical practice sites and IRBs covering their practice sites on the FDA Form 1572 in RCR to allow the following:

- Added to a site roster
- Assigned the treating, credit, consenting, or drug shipment (IVR only) tasks in OPEN
- Act as the site-protocol PI on the IRB approval
- Assigned the Clinical Investigator (CI) role on the Delegation of Tasks Log (DTL).

Additional information can be found on the CTEP website at <

https://ctep.cancer.gov/investigatorResources/default.htm >. For questions, please contact the RCR ***Help Desk*** by email at < RCRHelpDesk@nih.gov >.

4.2 Site Registration

This study is supported by the NCI Cancer Trials Support Unit (CTSU).

Each investigator or group of investigators at a clinical site must obtain IRB approval for this protocol and submit IRB approval and supporting documentation to the CTSU Regulatory Office before they can be approved to enroll patients. Assignment of site registration status in the CTSU Regulatory Support System (RSS) uses extensive data to make a determination of whether a site has fulfilled all regulatory criteria including but not limited to the following:

- An active Federal Wide Assurance (FWA) number
- An active roster affiliation with the Lead Network or a participating organization
- A valid IRB approval
- Compliance with all protocol specific requirements

In addition, the site-protocol Principal Investigator (PI) must meet the following criteria:

- Active registration status
- The IRB number of the site IRB of record listed on their Form FDA 1572

An active status on a participating roster at the registering site

Sites participating on the NCI CIRB initiative that are approved by the CIRB for this study are not required to submit IRB approval documentation to the CTSU Regulatory Office. For sites using the CIRB, IRB approval information is received from the CIRB and applied to the RSS in an automated process. Signatory Institutions must submit a Study Specific Worksheet for Local Context (SSW) to the CIRB via IRBManager to indicate their intent to open the study locally. The CIRB's approval of the SSW is then communicated to the CTSU Regulatory Office. In order for the SSW approval to be processed, the Signatory Institution must inform the CTSU which CIRB-approved institutions aligned with the Signatory Institution are participating in the study.

4.2.1 Downloading Regulatory Documents

(Note: For sites under the CIRB initiative, IRB data will automatically load to RSS.)

Site registration forms and the study Model Informed Consent may be downloaded from the 9782 protocol page located on the CTSU Web site. Permission to view and download this protocol is restricted and is based on person and site roster data housed in the CTSU RSS. To participate, Investigators and Associates must be associated with the Corresponding or Participating protocol organization in the RSS.

- Go to https://www.ctsu.org and log in using your CTEP IAM username and password
- Click on the Protocols tab in the upper left of your screen
- Either enter the protocol # in the search field at the top of the protocol tree, or
- Click on the By Lead Organization folder to expand, then LAO-NJ066, and

protocol #9782

- Click on LPO Documents tab, select the Site Registration documents link, and download and complete the forms provided. (Note: For sites under the CIRB initiative, IRB data will load to RSS as described above.)

Requirements For 9782 Site Registration:

- IRB approval (For sites not participating via the NCI CIRB; local IRB documentation, an IRB-signed CTSU IRB Certification Form, Protocol of Human Subjects Assurance Identification/IRB Certification/Declaration of Exemption Form, or combination is accepted)

4.2.2 Submitting Regulatory Documents

Submit required forms and documents to the CTSU Regulatory Office, where they will be entered and tracked in the CTSU RSS.

Regulatory Submission Portal: www.ctsu.org (members' area) → Regulatory Tab →Regulatory Submission
When applicable, original documents should be mailed to:
CTSU Regulatory Office
1818 Market Street, Suite 3000
Philadelphia, PA 19103

Institutions with patients waiting that are unable to use the Portal should alert the CTSU Regulatory Office immediately at 1-866-651-2878 in order to receive further instruction and support.

4.2.3 Checking **Site** Registration Status

You can verify your site registration status on the members' section of the CTSU website.

- Go to https://www.ctsu.org and log in to the members' area using your CTEP-IAM username and password
- Click on the Regulatory tab at the top of your screen
- Click on the Site Registration tab
- Enter your 5-character CTEP Institution Code and click on Go

Note: The status given only reflects compliance with IRB documentation and institutional compliance with protocol-specific requirements as outlined by the Lead Network. It does not reflect compliance with protocol requirements for individuals participating on the protocol or the enrolling investigator's status with the NCI or their affiliated networks.

4.3 Patient Registration

4.3.1 OPEN / IWRS

Patient enrollment will be facilitated using the Oncology Patient Enrollment Network (OPEN). OPEN is a web-based registration system available to users on a 24/7 basis. It is integrated with the CTSU Enterprise System for regulatory and roster data interchange and with the Theradex Interactive Web Response System (IWRS) for retrieval of patient registration/randomization assignment. Patient enrollment data entered by Registrars in OPEN / IWRS will automatically transfer to the NCI's clinical data management system, Medidata Rave.

For trials with slot reservation requirements, OPEN will connect to IWRS at enrollment initiation to check slot availability. Registration staff should ensure that a slot is available and secured for the patient before completing an enrollment.

The OPEN system will provide the site with a printable confirmation of registration and treatment information. Please print this confirmation for your records.

4.3.2 OPEN/IWRS User Requirements

OPEN/IWRS users must meet the following requirements:

- Have a valid CTEP-IAM account (*i.e.*, CTEP username and password).
- To enroll patients or request slot reservations: Be on an ETCTN Corresponding or Participating Organization roster with the role of Registrar. Registrars must hold a minimum of an AP registration type.
- To approve slot reservations or access cohort management: Be identified to Theradex as the "Client Admin" for the study.
- Have regulatory approval for the conduct of the study at their site.

Prior to accessing OPEN/IWRS, site staff should verify the following:

- All eligibility criteria have been met within the protocol stated timeframes.
- If applicable, all patients have signed an appropriate consent form and HIPAA authorization form.

4.3.3 OPEN/IWRS Questions?

Further instructional information on OPEN is provided on the OPEN tab of the CTSU website at https://www.ctsu.org or at https://open.ctsu.org. For any additional questions contact the CTSU Help Desk at 1-888-823-5923 or ctsucontact@westat.com.

Theradex has developed a Slot Reservations and Cohort Management User Guide, which is available on the Theradex website: http://www.theradex.com/clinicalTechnologies/?National-Cancer-Institute-NCI-11. This link to the Theradex website is also on the CTSU website OPEN tab. For questions about the use of IWRS for slot reservations, contact the Theradex Helpdesk: 609-619-7862 or Theradex main number 609-799-7580; CTMSSupport@theradex.com.

4.4 General Guidelines

Following registration, subjects should begin protocol treatment within 5 days. Issues that would cause treatment delays should be discussed with the Principal Investigators. If a subject does not receive protocol therapy following registration,

the subject's registration on the study may be canceled. The University of Wisconsin by the Cancer Therapy Discovery and Development (CTD2) research team should be notified of cancellations as soon as possible (608-263-6222).

5 TREATMENT PLAN

5.1 Agent Administration

Treatment will be administered on an outpatient basis. BSA should be recalculated if ≥ 10% weight loss. Reported adverse events and potential risks for Talazoparib (BMN 673), carboplatin and paclitaxel are described in Section 7. Appropriate dose modifications are described in Section 6. No investigational or commercial agents or therapies other than those described below may be administered with the intent to treat the patient's malignancy.

At each dose level, carboplatin will be administered intravenously on day 1 and paclitaxel intravenously on days 1, 8, 15 of a 21 day cycle. Carboplatin will be administered after the paclitaxel infusion is completed. Talazoparib (BMN 673) will be dosed orally starting on day 1 of each cycle prior to carboplatin and paclitaxel infusion. The starting dose of Talazoparib (BMN 673) for Schedule A will be at 25% of the single agent MTD (250 mcg once daily). Two MTDs will be determined from this phase I trial, one for each schedule.

In schedule A, Talazoparib (BMN 673) will be administered once daily for 7 days total. In schedule B, Talazoparib (BMN 673) will be administered once daily for 3 days total. Dose escalation for the two schedules will be conducted sequentially, starting with schedule A.

If no more than 4 of the evaluable 12 total patients treated at the MTD experienced a DLT, then the starting dose level for schedule B will be the MTD determined from Schedule A. If more than 4 patients experience a DLT with the dose expansion cohort at the MTD for schedule A, a conference call will be held with the sponsor to establish the starting dose for schedule B. If dose level -1 in Schedule A exceeds the MTD, then dose level -1 will be used as the starting dose for schedule B.

As of Amendment 7 (dated 9/20/17):

In Schedule A, 14 total patients have started treatment at dose level 2 (carboplatin AUC 6, paclitaxel 80mg/m2 and Talazoparib (BMN 673) 250mcg). Three were not evaluable in cycle 1 for DLT. Of the 11 evaluable patients, two experienced DLT (both with grade 3/4 neutropenia lasting > 7 days), 9 completed cycle 1 without DLT. The final patient has signed consent and is planned to start protocol therapy. Thus, no more than 3 patients will experience DLT at DL2 for schedule A. This meets the protocol criteria for MTD for schedule A being defined as dose level 2 which will be the starting dose level for schedule B.

Treatment will be continued until progressive disease, unacceptable toxicity or patient desire to discontinue study therapy. At any time after 4-6 cycles of combination therapy, the treating physician is given discretion whether to continue with carboplatin, paclitaxel and Talazoparib (BMN 673), continue carboplatin and Talazoparib (BMN 673) without paclitaxel, change to daily continuous dosing of Talazoparib (BMN 673) as a single agent, or observe the patient on study. All patients will be followed for the duration of study and until resolution or stabilization of any adverse effects related to study therapy. If the patient is continued on a) or b), subsequent change to c) at later timepoints is also allowed.

For patients being continued on single agent Talazoparib (BMN 673) who have completed 2 cycles of Talazoparib (BMN 673) alone, cycle duration is changed to 42 days.

Dose escalation will be conducted using a 3 + 3 design using the dose escalation scheme in the table below. Carboplatin dose will be escalated first given that a regimen with full dose carboplatin may be better accepted as a backbone for comparison in future studies as well as the biologic rationale that PARP inhibition and trapping potentiates the anti-tumor effect of these DNA damaging chemotherapies.

**Dose Escalation Schedule A**			
**Dose Level**	**Dose**		
	**Carboplatin (AUC) IV Day 1**	**Paclitaxel (mg/m^2) IV Days 1, 8, 15**	**Talazoparib (BMN 673) (mcg) PO once daily, Days 1-7[$#]**
Level -1	5	80	100
Level 1[*]	5	80	250
Level 2	6	80	250
Level 3	6	80	350
Level 4	6	80	500
Level 5	6	80	750
Level 6	6	80	1000

*Starting dose level for schedule A.
[$] For schedule B, Talazoparib (BMN 673) will be dosed days 1-3.
[#] At any time after 4-6 cycles of combination therapy, for patients with clinical response or stable disease, extension of therapy is allowed with a) carboplatin, paclitaxel and Talazoparib (BMN 673), b) carboplatin and Talazoparib (BMN 673) without paclitaxel, c) Talazoparib (BMN 673) once daily on days 1-21 (continuous dosing). If the patient is continued on a) or b), subsequent change to c) at later timepoints is also allowed. Patients with no prior dose modification of Talazoparib (BMN 673) should have the dose changed to 1000mcg per day (the single agent MTD). Patients who met criteria for a dose modification of Talazoparib (BMN 673) should be continued at the Talazoparib (BMN 673) dose required after the modification, or d) observation without therapy per discretion of the treating provider.

**Dose Escalation Schedule B**			
**Dose Level**	**Dose**		
	**Carboplatin (AUC) IV Day 1**	**Paclitaxel (mg/m^2) IV Days 1, 8, 15**	**Talazoparib (BMN 673) (mcg) PO once daily, Days 1-3**
Level -1	5	80	250
Level 1*	6	80	250
Level 2	6	80	350
Level 3	6	80	500
Level 4	6	80	750
Level 5	6	80	1000

* Starting dose level

**Regimen A Description**					
**Agent**	**Pre-medications; Precautions**	**Dose**	**Route**	**Schedule**	**Cycle Length**
Talazoparib (BMN 673)	May take with or without meal	**	PO in the A.M.	Days 1-7 prior to carboplatin and paclitaxel infusion[#]	21 days (3 weeks)
Carboplatin	Dexamethasone<br>5-HT3 antagonist	** in D5W or normal saline	IV	Day 1 after paclitaxel infusion is completed	
	Dexamethasone<br>5-HT3 antagonist<br>Diphenhydramine<br>Ranitidine	** in D5W or normal saline	IV	Days 1, 8, 15	

**Doses and volume of diluent as appropriate for assigned dose level and per institutional standard.
[#] At any time after 4-6 cycles of combination therapy, for patients with clinical response or stable disease, extension of therapy is allowed with a) carboplatin, paclitaxel and Talazoparib (BMN 673), b) carboplatin and Talazoparib (BMN 673) without paclitaxel, c) Talazoparib (BMN 673) once daily on days 1-21 (continuous dosing). If the patient is continued on a) or b), subsequent change to c) at later timepoints is also allowed. Patients with no prior dose modification of Talazoparib (BMN 673) should have the dose changed to 1000mcg per day (the single agent MTD). Patients who met criteria for a dose modification of Talazoparib (BMN 673) should be continued at the Talazoparib (BMN 673) dose required after the modification, or d) observation without therapy per discretion of the treating provider.

**Regimen B Description**					
**Agent**	**Pre-medications; Precautions**	**Dose**	**Route**	**Schedule**	**Cycle Length**
Talazoparib (BMN 673)	May take with or without meal	**	PO in the A.M.	Days 1-3 prior to carboplatin and paclitaxel infusion[#]	21 days (3 weeks)
Carboplatin	Dexamethasone<br>5-HT3 antagonist	** in D5W or normal saline	IV	Day 1 after paclitaxel infusion is completed	
Paclitaxel	Dexamethasone<br>5-HT3 antagonist<br>Diphenhydramine<br>Ranitidine	** in D5W or normal saline	IV	Days 1, 8, 15	

**Doses and volume of diluent as appropriate for assigned dose level and per institutional standard.
[#] At any time after 4-6 cycles of combination therapy, for patients with clinical response or stable disease, extension of therapy is allowed with a) carboplatin, paclitaxel and Talazoparib (BMN 673), b) carboplatin and Talazoparib (BMN 673) without paclitaxel, c) Talazoparib (BMN 673) once daily on days 1-21 (continuous dosing). If the patient is continued on a) or b), subsequent change to c) at later timepoints is also allowed. Patients with no prior dose modification of Talazoparib (BMN 673) should have the dose
changed to 1000mcg per day (the single agent MTD). Patients who met criteria for a dose modification of Talazoparib (BMN 673) should be continued at the Talazoparib (BMN 673) dose required after the modification, or d) observation without therapy per discretion of the treating provider.

NOTE: The patient will be requested to maintain a medication diary of each dose of medication. The medication diary will be returned to clinic staff at the end of each course. (Appendix B)

5.1.1 **Talazoparib (BMN 673)**

Talazoparib (BMN 673) will be administered orally without regard to meals. Talazoparib (BMN 673) will be given orally daily for the first 7 days in treatment cycles 1 and beyond in schedule A. Talazoparib (BMN 673) will be given orally daily for the first 3 days in treatment cycles 1 and beyond in schedule B. At any time after 4-6 cycles of the combination therapy, for patients with clinical benefit defined as response or stable disease, extension of therapy is allowed at the treating provider’s discretion with either:

a) carboplatin, paclitaxel and Talazoparib (BMN 673),
b) carboplatin and Talazoparib (BMN 673) without paclitaxel,
c) Talazoparib (BMN 673) daily continuous dosing, or
d) observation without therapy per discretion of the treating provider.

If the patient is continued on a) or b), subsequent change to c) at later timepoints is also allowed. For patients being continued on single agent Talazoparib (BMN 673) who have completed 2 cycles of Talazoparib (BMN 673) alone, cycle duration is changed to 42 days.

If a subject forgets to take a dose at the regularly scheduled dosing time, the dose may be taken up to 12 hours later. Doses not taken before the end of this 12 hour extension period will be considered missed. Missed doses should not be made up. Vomited doses should not be retaken. For missed, late, and vomited doses, the subject's regular dosing schedule should be resumed with the following dose.

On the days when the patient is scheduled to undergo sampling of blood for correlative studies, the dose of Talazoparib (BMN 673) will be administered under supervision (to record the time and coordinate collection of subsequent samples).

Because there is a potential for interaction of Talazoparib (BMN 673) with other concomitantly administered drugs, the case report form must capture the concurrent use of all other drugs, over-the-counter medications, or alternative therapies.

Talazoparib (BMN 673) is not known to be an inhibitor, inducer or substrate of the major human CYPs in vitro, indicating a low risk for drug-drug interactions at the proposed dosing.

5.1.2 **Paclitaxel (commercially obtained)**

Paclitaxel should be diluted in Dextrose 5% or Sodium Chloride 0.9% per institutional standard, and given by intravenous administration. The concentration of the final solution should be between 0.3 and 1.2 mg/mL. Prepare in non-PVC infusion container and administer IV over one hour via 0.22 micron in line filter and non-DEHP tubing. The calculated dose of paclitaxel should be administered via a free flowing intravenous line as a one hour infusion. Solution exhibiting excessive particulate formation should be discarded.

Concentrations of up to 1.2 mg/ml in 5% dextrose or normal saline solution have demonstrated chemical and physical stability for at least 27 hours at room temperature.

Due to known allergic reactions to paclitaxel and/or of the Cremophor® vehicle, the following precautions must be taken to minimize the chances of hypersensitivity reaction.

Suggested pre-medications for Paclitaxel

Agent	Dose	Route	Duration
Dexamethasone	* 20 mg	PO	12 and 6 hours prior to paclitaxel
5-HT3 Antagonist	**	PO	Per institutional guidelines, prior to infusion of paclitaxel
Diphenhydramine	50 mg	IV	30 minutes prior to paclitaxel
Ranitidine	50 mg	IV	30 minutes prior to paclitaxel

* 20 mg is the dose for cycle 1, day 1. Subsequently, may be decreased to 12mg on cycle 1 day 8 and 8mg on cycle 1 day 15 and all future doses of paclitaxel. Alternatively, a single intravenous dose of 20 mg, 30 minutes prior to Paclitaxel (Taxol) injection, only when in the investigator's opinion, patients may have been non-adherent with oral pre-medication.

** Dose to be based on 5-HT3 Antagonist used.

Epinephrine and diphenhydramine for injection should be readily available during the infusion for emergency treatment of hypersensitivity reactions.

Note: Pre-medications can be adjusted/altered to meet local institutional standards.

5.1.3 **Carboplatin (commercially obtained)**

Carboplatin will be administered after the paclitaxel infusion is completed. Carboplatin at the appropriate dose will be given intravenously as a 30- minute infusion in Dextrose 5% in Water or Sodium Chloride 0.9%, volume per institutional standard.

Carboplatin Dose Calculation and Administration

Carboplatin dose will be calculated using the Calvert formula:

Total Dose (mg) = target AUC* (GFR+25), GFR may be substituted by CrCL·

Note: Calculated total dose is in mg -not mg/m^2

The Creatinine Clearance [75] (replaces GFR in Calvert formula) will be calculated for each treatment course using the formula:

$$CrCl = \frac{(140 - age) * weight\ (kg)}{72 * serum\ creatinine} (* 0.85\ if\ female)$$

The minimum serum creatinine value used will be 0.7 mg/dL and a CrCl cap will be 125 mL/min.

Questions about this calculation should be directed to the principal investigator.

Note: Remember to re-calculate the dose for each treatment cycle. The actual body weight should be used for all calculations.

5.1.4. Supportive care measures

Growth factor: The use of prophylactic granulocyte colony stimulating factor is not allowed for the first cycle of therapy. The use of prophylactic growth factors should follow the ASCO guidelines, and could be administered starting with cycle 2. The use of growth factors for the treatment of anemia is allowed at the discretion of the treating physician. Patients may receive red blood cell and platelet transfusions as clinically indicated by the treating physician. Empiric platelet transfusions are recommended for platelet count ≤10,000/mcL.

Supportive care agents for bone metastases: Patients with known metastatic disease to the bones are allowed to take bisphosphonates or denosumab as directed by the treating physician.

<u>Post-treatment anti-emetic medications:</u>

The treating physician may prescribe dexamethasone 4 mg orally every 12 hours for six doses beginning in the evening of the day of chemotherapy infusion (or per institutional standard). It is recommended that patients be given take home prescriptions for an oral 5- HT3 antagonist (i.e. ondansetron, granisetron) to take as needed for nausea or emesis. Aprepitant or fosaprepitant are allowed on study per institutional standards.

<u>Other Modality(ies) or Procedure</u>s-N/A

5.2 Definition of Dose-Limiting Toxicity

The MTD will be defined as the highest safely tolerated dose where 0/6 or 1/6 (less than 33%) patients experience a DLT and two or more patients have experienced a DLT at the next higher dose level. The MTD will be used as the RP2D unless the investigators and the NCI determine a lower dose level is recommended after observations of post-cycle 1 and 2 adverse events in patients. A dose expansion cohort of 6 subjects at the MTD will also be used for further characterization of the safety and tolerability of this regimen to determine the RP2D.

Patients will be evaluated for DLT during the first cycle of protocol (i.e. From cycle 1, day 1 until cycle 2, day 1). A one cycle DLT period is felt to be adequate for this schedule combining intermittent Talazoparib (BMN 673) with these chemotherapy agents since myelosuppression and gastrointestinal toxicities would be anticipated to occur early.

Patients must complete at least day 1 through day 8 of planned cycle 1 treatment to be considered evaluable, unless a DLT occurs in this timeframe. Patients who do not complete cycle 1 for reasons other than safety (e.g., withdrawal of consent, non-compliance, disease progression) OR experiences a study treatment delay within Cycle 1 of more than 7 days for reasons other than safety, will be inevaluable and he or she will be replaced. Dose modifications for some toxicities

known to be due to paclitaxel or carboplatin are permitted in section 6.0 and may not meet criteria for DLT. Adverse events at least possibly attributable to the study drugs will be used to constitute DLT. CTCAE v4.0 will be used for toxicity grading until March 31, 2018. CTCAE version 5.0 will be utilized beginning April 1, 2018. Furthermore, all patients will be also monitored and evaluated for toxicities and adverse events occurring after the first cycle.

Participating sites will hold conference calls at least twice monthly to discuss trial status, ongoing adverse events, DLTs and dose escalation plans.

Occurrence of one or more of the following during the first cycle of treatment will constitute DLT:

Hematologic:

Absolute neutrophil count < 1000/mcL lasting longer than 7 days.

Grade 4 thrombocytopenia (platelet < 25,000/mcL) lasting longer than 7 days, or Grade 3 or 4 thrombocytopenia associated with clinically significant bleeding or Grade 4 thrombocytopenia requiring empiric platelet transfusion (platelet count < 10,000/mcl).

Grade 3 or 4 neutropenia associated with sepsis or fever ≥ 38°C lasting at least 1 hour or a single temperature higher than 38.3°C.

Delay starting cycle 2 by more than 2 weeks due to toxicity.

Non-hematologic:

Grade 3 or higher non-hematological toxicity except nausea, vomiting, diarrhea, electrolyte abnormalities or clinically insignificant laboratory changes (must be discussed with PI or study chair), and fatigue.

Grade 3 or higher nausea, vomiting, diarrhea or electrolyte abnormalities that last longer than 48 hours despite maximal medical therapy or fatigue lasting more than 7 days despite maximal medical therapy.

Delay starting cycle 2 by more than 2 weeks due to toxicity.

Management and dose modifications associated with the above adverse events are outlined in Section 6.

Dose escalation will proceed within each cohort (schedule A and schedule B) according to the following scheme. Dose-limiting toxicity (DLT) is defined above.

Number of Patients with DLT at a Given Dose Level	Escalation Decision Rule
0 out of 3	Enter 3 patients at the next dose level.
≥2	Dose escalation will be stopped. This dose level will be declared the maximally administered dose (highest dose administered). Three (3) additional patients will be entered at the next lowest dose level if only 3 patients were treated previously at that dose.
1 out of 3	Enter at least 3 more patients at this dose level.<br>• If 0 of these 3 patients experience DLT, proceed to the next dose level.<br>• If 1 or more of this group suffer DLT, then dose escalation is stopped, and this dose is declared the maximally administered dose. Three (3) additional patients will be entered at the next lowest dose level if only 3 patients were treated previously at that dose.
≤1 out of 6 at highest dose level below the maximally administered dose	This is the MTD. At least 6 patients must be entered at the MTD.

5.3 General Concomitant Medication and Supportive Care Guidelines

In case participants develop nausea/vomiting or myelosuppression, supportive medications will be prescribed as per section 5.1.4. Further management of these events as well as diarrhea are outlined in section 6.

For patient who develop an indication to start anticoagulation during study therapy (ie. a DVT or PE), anticoagulants including warfarin (with PT/INR monitoring), low molecular weight heparin, and factor Xa inhibitors are permitted and the patient may stay on trial as long as they personally have not developed grade 3 or worse thrombocytopenia or medically significant bleeding at the current dose level that they are receiving on trial. Caution with close monitoring of platelet counts and for any signs/symptoms of bleeding is recommended if the patient subsequently is changed to Talazoparib (BMN 673) alone at a higher dose (ie. weekly for first cycle).

Because there is a potential for interaction of paclitaxel with other concomitantly administered drugs through the cytochrome P450 system, the case report form must capture the concurrent use of all other drugs, over-the-counter medications, or alternative therapies. The Principal Investigator should be alerted if the patient is taking any agent known to affect or with the potential to affect selected CYP450 isoenzymes. Participants should be given the handout and wallet card provided in Appendix D.

While paclitaxel is being administered, the following medications are contraindicated or must be used with caution:

Contraindicated (see Appendix C for list):

- CYP2C8 strong and moderate inhibitors
- CYP2C8 inducers
- CYP3A4 strong and moderate inhibitors
- CYP3A4 inducers
- CYP3A4 sensitive substrates

Exclusions: The following supportive care medications will be allowed as they are routinely administered with carboplatin and paclitaxel and have no potential interaction with Talazoparib (BMN 673): dexamethasone, aprepitant, fosaprepitant and ondansetron.Other contraindicated medications (per above) are not allowed unless the in the judgement of the treating doctor, close monitoring with labs or drug levels or by symptoms with subsequent dose adjustments is feasible.

Patients taking contraindicated concurrent medications that do not meet these exclusion criteria are ineligible unless they can discontinue or switched to alternative medications prior to initiation of the study drug (at least 5 half-lives).

Use with Caution:

- CYP2C8 sensitive substrates
- CYP2C8 weak inhibitors
- CYP3A4 non-sensitive substrates
- CYP3A4 weak inhibitors

These agents may be permitted if discontinuation is not feasible and no acceptable alternatives are available as determined by the treating physician; however, caution should be used. Consider monitoring with labs or drug levels or based on symptomsand consider dose adjustments of the medication

5.4 Duration of Therapy

In the absence of treatment delays due to adverse event(s), treatment may continue for 4-6 cycles. After 4-6 cycles, treatment may be continued at the discretion of the treating physician. The physician may continue combination study therapy with a) Talazoparib (BMN 673), carboplatin and paclitaxel or b) Talazoparib (BMN 673) and carboplatin without paclitaxel or c) single agent Talazoparib (BMN 673) or d) the patient may enter an observation period with no treatment (neither study treatment nor an alternative standard of care). The decision to continue carboplatin and Talazoparib (BMN 673) without paclitaxel or Talazoparib (BMN 673) alone will not be considered a dose reduction. Patients will remain on study until one of the following criteria applies:

- Disease progression,
- Intercurrent illness that prevents further administration of treatment,
- Unacceptable adverse event(s),
- Patient decides to withdraw from the study,
- General or specific changes in the patient's condition render the patient unacceptable for further treatment in the judgment of the investigator,
- Patient becomes pregnant or begins breast-feeding,
- Patient required > 2 dose reductions of chemotherapy or dose reductions of Talazoparib (BMN 673) to a dose less than 100mcg (either alone or in combination with chemotherapy), or
- Patient experiences treatment related toxicities that lead to a greater than 2 week delay in initiation of the next cycle of therapy. For a specific isolated adverse event, a total of 2 week delay is allowed, but the patient may not be delayed for any longer than 3 weeks sequentially, due to all treatment related toxicities observed during this time period. If all treatment related toxicities do not recover within 3 weeks to the point where patients meet re-treatment criteria in section 6.0, then patients should be removed from therapy on trial. However, if

the patient is clinically benefiting if the benefits are felt to outweigh the risks after discussion between the treating doctor and study chair, the patients may resume study therapy with dose reduction.

5.5 Duration of Follow Up

Patients will be followed for 4 weeks after removal from study or until death, whichever occurs first. Patients removed from study for unacceptable adverse event(s) will be followed until resolution or stabilization of the adverse event.

5.6 Criteria for Removal from Study

Patients will be removed from study when any of the criteria listed in Section 5.4 applies. The reason for study removal and the date the patient was removed must be documented in the Case Report Form.

6 DOSING DELAYS/DOSE MODIFICATIONS

Patients must meet the following treatment parameters:

Parameter	Day 1	Days 8, 15
Absolute Neutrophil Count (ANC)	≥1,000/mcL	≥1,000/mcL
Platelet Count<br><br>Total Bilirubin	≥100,000/mcL**<br><br>≤1.25x ULN, with the exception of <2.9 mg/dL for patients with Gilbert's disease	≥75,000/mcL<br><br>≤1.25x ULN*, with the exception of <2.9 mg/dL for patients with Gilbert's disease
AST (SGOT)/ALT (SGPT)	≤2.5x ULN; ≤ 5x ULN in setting of metastatic liver disease	≤2.5x ULN; ≤ 5x ULN in setting of metastatic liver disease*
Creatinine	≤1.5 x ULN<br>≥50 mL/min	N/A

* only applicable if on study calendar (Section 10.0)
**For patients on BMN 673 (talazoparib) alone who have completed at least one cycle of talazoparib alone, subsequent day 1 platelet count treatment parameter will be ≥75,000/mcL

All other toxicities (except alopecia, lymphopenia, hyperglycemia, hypoalbuminemia, fatigue, elevated serum alkaline phosphatase, neuropathy [see section 6.2.1] and hemoglobin [see section 6.1.3]) at least possibly related to study treatment should have resolved to grade 1 or lesser severity or pre-study baseline before initiation of the next cycle of therapy. Furthermore, dose modifications for WBC or other components of the differential (such as lymphocytes, monocytes) are not planned and are therefore, not included in section 6.1.

Qualifying laboratory tests can be obtained 24-72 hours before planned initiation of therapy as per study calendar (Section 10).

Dose holds and modifications are to be made according to the organ system showing the greatest degree of toxicity. Toxicity will be graded using the National Cancer Institute Common Terminology Criteria for Adverse Events (CTCAE) (version 4.0 until March 31, 2018, then version 5.0 will be utilized beginning April 1, 2018).

Tables below are general guidance for adverse events. Treating physicians may use discretion to hold or reduce dose for these events with the approval of the Principal Investigator or Study Chair.

If a toxicity leads to hold on day 1 of a cycle, the whole cycle is to be delayed until the toxicity meets treatment criteria. If a toxicity leads to a hold of paclitaxel during the cycle (i.e. day 8 or 15), the dose will be skipped and the dose modification will be with the next scheduled dose.

Initiation of the next cycle of therapy may be delayed no more than two weeks to allow recovery from toxicity. Treatment delay of > 2 weeks due to a specific toxicity to all protocol therapy at least possibly related to study drugs will lead to removal of the patient from the study. For a specific isolated adverse event, a total of 2 week delay is allowed, but the patient may not be delayed for any longer than 3 weeks sequentially, due to all treatment related toxicities observed during this

time period. If all treatment related toxicities do not recover, within 3 weeks to the point where patients meet re-treatment criteria in section 6.0 above, then patients should be removed from therapy on trial. However, if the patient is clinically benefiting if the benefits are felt to outweigh the risks after discussion between the treating doctor and study chair, the patients may resume study therapy with dose reduction.

All dose reductions are permanent. Dose reductions are permitted until Talazoparib (BMN 673) dose is 100mcg alone or in combination with chemotherapy.

Since fatigue is often multifactorial and can be a symptom of cancer progression, dose reduction for fatigue will only be done if the fatigue is deemed to be drug-related in the opinion of the investigator.

**Dose Modification Levels**	
**Agent**	**Dose**
**Carboplatin**	**AUC 6**<br>**AUC 5**<br>**AUC 4**<br>**AUC 3**
**Paclitaxel**	**80 mg/m^2**<br>**60 mg/m^2**<br>**45 mg/m^2**
**Talazoparib (BMN 673)**	**1000 mcg**<br>**750 mcg**<br>**500 mcg**<br>**350 mcg**<br>**250 mcg**<br>**100 mcg**

6.1 Dose Modifications for Hematological Toxicity

6.1.1 Neutrophils

The following dose adjustments are based on the neutrophil nadir of the preceding treatment course.

Neutropenia**	Management/Next Dose for Talazoparib (BMN 673)#	Management/Next Dose for Paclitaxel	Management/Next Dose for Carboplatin
**≤ Grade 1/2**	No change in dose	No change in dose	No change in dose
**Grade 3/4 (≤7 days and without fever)**	Hold* until ≤ Grade 2. Resume at same dose level.	Hold* until ≤ Grade 2. Resume at same dose level.	Hold* until ≤ Grade 2. Resume at same dose level.
**Grade 3/4 (>7 days or any duration with fever) -- 1st episode**	Hold* until ≤ Grade 2. Resume at same dose level.	Hold* until ≤ Grade 2. Reduce by 1 dose level.	Hold* until ≤ Grade 2. Reduce by 1 dose level.
**Grade 3/4 (>7 days or any duration with fever) -- 2nd episode**	Hold* until ≤ Grade 2. Reduce by 1 dose level.*	Hold* until ≤ Grade 2. Reduce by 1 dose level.	Hold* until ≤ Grade 2. Reduce by 1 dose level.

*Patients requiring a delay of all protocol therapy >2 weeks due to toxicity should go off protocol therapy.
**Patients requiring > two dose reductions of chemotherapy should go off protocol therapy.
Dose reductions are permitted for Talazoparib (BMN 673) until dose is 100mcg alone or in combination with chemotherapy.
If a toxicity leads to hold on day 1 of a cycle, the whole cycle is to be delayed until the toxicity meets treatment criteria. If a toxicity leads to a hold of paclitaxel during the cycle (i.e. day 8 or 15), the dose will be skipped and the dose modification will be with the next scheduled dose.
For patients being treated with single agent daily (days 1-21) Talazoparib (BMN 673), dose should be held for any grade 3-4 neutropenia with or without fevers. Dose should be held until ≤ Grade 2 and then resumed at 1 lower dose level.

NOTE: Use of prophylactic granulocyte colony stimulating factor in cycle 2 or beyond is permitted per investigator discretion as long as chemotherapy is also being administered with Talazoparib (BMN 673). Use of ASCO guidelines is recommended.

6.1.2 **Platelets**

**Thrombocytopenia****	**Management/Next Dose for Talazoparib (BMN 673)**[#]	**Management/Next Dose for Paclitaxel**	**Management/Next Dose for Carboplatin**
**≤ Grade 1**	No change in dose	No change in dose	No change in dose
**Grade 2**	Hold until ≥ 100,000. Resume at same dose level.	Hold dose until ≤ Grade 1. Resume at same dose level.	Hold until ≥ 100,000. Resume at same dose level.
**Grade 3/4 lasting ≤ 7 days and no clinically significant bleeding and values > 10,000 mcL**	Hold* until ≥ 100,000. Resume at same dose level.	Hold* dose until ≥ 100,000. Resume at same dose level.	Hold* until ≥ 100,000. Resume at same dose level.
**Grade 4 lasting > 7 days OR requiring empiric platelet transfusion (≤10,000 mcL) -- 1st episode**	Hold* until ≥ 100,000. Reduce by 1 dose level.	Hold* until ≥ 100,000. Resume at same dose level.	Hold* until ≥ 100,000. Reduce by 1 dose level.
**Grade 4 lasting > 7 days OR requiring empiric platelet transfusion (≤10,000 mcL) -- 2nd episode**	Hold* until ≥ 100,000. Reduce by 1 dose level.	Hold* until ≥ 100,000. Reduce by 1 dose level.	Hold* until ≥ 100,000. Reduce by 1 dose level.
**Grade 3/4 of any duration with clinically significant bleeding*****	Off protocol	Off protocol	Off protocol

*Patients requiring a delay of all protocol therapy >2 weeks due to toxicity should go off protocol therapy.<br>**Patients requiring > two dose reductions of chemotherapy should go off protocol therapy. Dose reductions are permitted for Talazoparib (BMN 673) until dose is 100mcg alone or in combination with chemotherapy.<br><br>If a toxicity leads to hold on day 1 of a cycle, the whole cycle is to be delayed until the toxicity meets treatment criteria. If a toxicity leads to a hold of paclitaxel during the cycle (i.e. day 8 or 15), the dose will be skipped and the dose modification will be with the next scheduled dose.<br><br>*** Clinically significant bleeding for this protocol is defined as potentially life-threatening (i.e. GI bleed, intracranial hemorrhage). Patients with more minor bleeding may be continued on study.<br><br># For patients being treated with single agent daily (days 1-21) Talazoparib (BMN 673), dose should be held for any grade 2 or higher thrombocytopenia with or without bleeding. Dose should be held until < Grade 2 and then resumed at 1 lower dose level unless the event was Grade 3/4 of any duration with clinically significant bleeding. Then the patient should be removed from the protocol therapy.

6.1.2 **Hemoglobin**

The hemoglobin parameters must meet the criteria specified in section 3.1.6 of the protocol on day 1 of each treatment cycle. Treatment should be delayed until recovery of the counts to the specified eligibility levels. Transfusion is permitted after Cycle 1, Day 1 and future cycles for subjects who do not meet the hemoglobin parameters (section 3.1.6) for retreatment.

6.2 Dose modifications for non-hematological toxicity

6.2.1 Peripheral Sensory Neuropathy

The following dose adjustments are based on the worst grade experience of sensory neuropathy of any preceding treatment course.

**Sensory neuropathy** **	**Management/Next Dose for Talazoparib (BMN 673)#**	**Management/Next Dose for Paclitaxel****	**Management/Next Dose Carboplatin**
**≤ Grade 1**	No change in dose	No change in dose	No change in dose
**Grade 2, tolerable and <7 consecutive days**	No change in dose	No change in dose	No change in dose
**Grade 2, intolerable OR >7 consecutive days**	No change in dose	Hold*dose until < Grade 2. Reduce by 1 dose level.	No change in dose unless no longer receiving paclitaxel, then hold until < Grade 2. Reduce by one dose level.
**Grade 3**	No change in dose	Discontinue paclitaxel	Hold* until < Grade 2. Reduce by one dose level.
**Grade 4**	Off study	Off study	Off study

* Patients requiring a delay of all protocol therapy >2 weeks due to toxicity should go off protocol therapy.<br>**Patients requiring > two dose reductions of chemotherapy should go off protocol therapy.<br><br>If a toxicity leads to hold on day 1 of a cycle, the whole cycle is to be delayed until the toxicity meets treatment criteria. If a toxicity leads to a hold of paclitaxel during the cycle (i.e. day 8 or 15), the dose will be skipped and the dose modification will be with the next scheduled dose.<br><br>After 4-6 cycles, paclitaxel may be discontinued per investigator discretion (including for neurotoxicity) and patient may continue on study.<br><br># For patients being treated with single agent daily (days 1-21) Talazoparib (BMN 673), dose should be held for any grade 3 or higher sensory neuropathy at least possibly related to Talazoparib (BMN 673). Dose should be held until < Grade 2 and then resumed at 1 lower dose level.

6.2.2 Arthralgia/Myalgia

Use CTCAE bone pain criteria for grading of arthralgias.

Arthralgia/Myalgia**	Management/Next Dose for Talazoparib (BMN 673)[#]	Management/Next Dose for Paclitaxel	Management/Next Dose Carboplatin
**≤ Grade 1**	No change in dose	No change in dose	No change in dose
**Grade 2[a]**	No change in dose	Hold* until < Grade 2. Reduce by 1 dose level.	No change in dose
**Grade 3**	No change in dose	Hold* until < Grade 2. Reduce by 1 dose level.	No change in dose

*Patients requiring a delay of all protocol therapy >2 weeks due to toxicity should go off protocol therapy.
**Patients requiring > two dose reductions of chemotherapy should go off protocol therapy.

a) For first occurrence of grade 2 myalgia/arthralgia, dexamethasone can be administered for 3- 4 days (approximately 4 mg BID) after chemotherapy. If symptoms recur despite this, the next dose of paclitaxel will be reduced by 1 dose level.

If a toxicity leads to hold on day 1 of a cycle, the whole cycle is to be delayed until the toxicity meets treatment criteria. If a toxicity leads to a hold of paclitaxel during the cycle (i.e. day 8 or 15), the dose will be skipped and the dose modification will be with the next scheduled dose.

[#] For patients being treated with single agent daily (days 1-21) Talazoparib (BMN 673), dose should be held for any grade 3 or higher arthralgia/myalgia at least possibly related to Talazoparib (BMN 673). Dose should be held until < Grade 2 and then resumed at 1 lower dose level.

6.2.3 Gastrointestinal toxicity

Nausea and/or vomiting should be controlled with adequate antiemetic therapy. Prophylactic anti-emetic therapy can be used at the discretion of the treating physician. Diarrhea should be managed with adequate anti-diarrheal medications. Patients are encouraged to take plenty of oral fluids. Dose holds and modifications are for symptoms at least possible related to the study therapy and occurring despite maximal medical management.

<u>**Nausea**</u>**	**Management/Next Dose for Talazoparib (BMN 673)**[#]	**Management/Next Dose for Paclitaxel**	**Management/Next Dose for Carboplatin**
**≤ Grade 1**	No change in dose	No change in dose	No change in dose
**Grade 2**	Hold* until ≤ Grade 1. Resume at same dose level.	Hold* until ≤ Grade 1. Resume at same dose level.	Hold* until ≤ Grade 1. Resume at same dose level.
**Grade 3**	Hold* until < Grade 2. Resume at same dose level.	Hold* until < Grade 2. Reduce by 1 dose level.	Hold* until < Grade 2. Reduce by 1 dose level.

After optimal anti-emetic therapy. Use of prophylactic or scheduled antiemetics are recommended in future cycles for patients experiencing grade 2 or higher nausea.
* Patients requiring a delay of all protocol therapy >2 weeks due to toxicity should go off protocol therapy.
** Patients requiring > two dose reductions of chemotherapy should go off protocol therapy. Dose reductions are permitted for Talazoparib (BMN 673) until dose is 100mcg alone or in combination with chemotherapy.
If a toxicity leads to hold on day 1 of a cycle, the whole cycle is to be delayed until the toxicity meets treatment criteria. If a toxicity leads to a hold of paclitaxel during the cycle (i.e. day 8 or 15), the dose will be skipped and the dose modification will be with the next scheduled dose.

For patients being treated with single agent daily (days 1-21) Talazoparib (BMN 673), dose should be held for any grade 2 or higher nausea at least possibly related to Talazoparib (BMN 673) and occurring despite maximal medical management. Dose should be held until < Grade 2 and then resumed at 1 lower dose level.

<u>**Vomiting**</u>**	**Management/Next Dose for Talazoparib (BMN 673)#**	**Management/Next Dose for Paclitaxel**	**Management/Next Dose for Carboplatin**
**≤ Grade 1**	No change in dose	No change in dose	No change in dose
**Grade 2**	Hold***** until ≤ Grade 1. Resume at same dose level.	Hold***** until ≤ Grade 1. Resume at same dose level.	Hold***** until ≤ Grade 1. Resume at same dose level.
**Grade 3**	Hold***** until < Grade 2. Resume at same dose level.	Hold***** until < Grade 2. Reduce by 1 dose level.	Hold***** until < Grade 2. Reduce by 1 dose level.
**Grade 4**	Hold*****until < Grade 2. Reduce by 1 dose level.	Hold***** until < Grade 2. Reduce by 1 dose level.	Hold***** until < Grade 2. Reduce by 1 dose level.

After optimal anti-emetic therapy. Use of prophylactic or scheduled antiemetics are recommended in future cycles for patients experiencing grade 2 or higher emesis.

*Patients requiring a delay of all protocol therapy >2 weeks due to toxicity should go off protocol therapy.
**Patients requiring > two dose reductions of chemotherapy should go off protocol therapy. Dose reductions are permitted for Talazoparib (BMN 673) until dose is 100mcg alone or in combination with chemotherapy.

If a toxicity leads to hold on day 1 of a cycle, the whole cycle is to be delayed until the toxicity meets treatment criteria. If a toxicity leads to a hold of paclitaxel during the cycle (i.e. day 8 or 15), the dose will be skipped and the dose modification will be with the next scheduled dose.

For patients being treated with single agent daily (days 1-21) Talazoparib (BMN 673), dose should be held for any grade 2 or higher vomiting at least possibly related to Talazoparib (BMN 673) and occurring despite maximal medical management. Dose should be held until < Grade 2 and then resumed at 1 lower dose level

<u>**Diarrhea****</u>	**Management/Next Dose for Talazoparib (BMN 673)[#]**	**Management/Next Dose for Paclitaxel**	**Management/Next Dose for Carboplatin**
**≤ Grade 1**	No change in dose	No change in dose	No change in dose
**Grade 2**	Hold***** until ≤ Grade 1. Resume at same dose level.	Hold***** until ≤ Grade 1. Resume at same dose level.	Hold***** until ≤ Grade 1. Resume at same dose level.
**Grade 3**	Hold***** until < Grade 2. Resume at same dose level.	Hold***** until < Grade 2. Reduce by 1 dose level.	Hold***** until < Grade 2. Reduce by 1 dose level.
**Grade 4**	Off study	Off study	Off study

*****Patients requiring a delay of all protocol therapy >2 weeks due to toxicity should go off protocol therapy.
******Patients requiring > two dose reductions of chemotherapy should go off protocol therapy. Dose reductions are permitted for Talazoparib (BMN 673) until dose is 100mcg alone or in combination with chemotherapy.

Note: After optimal anti-diarrheal therapy

If a toxicity leads to hold on day 1 of a cycle, the whole cycle is to be delayed until the toxicity meets treatment criteria. If a toxicity leads to a hold of paclitaxel during the cycle (i.e. day 8 or 15), the dose will be skipped and the dose modification will be with the next scheduled dose.

[#] For patients being treated with single agent daily (days 1-21) Talazoparib (BMN 673), dose should be held for any grade 2 or higher diarrhea at least possibly related to Talazoparib (BMN 673) and occurring despite maximal medical management. Dose should be held until < Grade 2 and then resumed at 1 lower dose level unless the event was Grade 4. Then the patient should be removed from the protocol therapy.

Recommended management: Loperamide antidiarrheal therapy
Dosage schedule: 4 mg at first onset, followed by 2 mg with each loose motion until diarrhea-free for 12 hours (maximum dosage: 16 mg/24 hours)
Adjunct anti-diarrheal therapy is permitted and should be recorded when used.

6.2.4 Hepatic toxicity

There are no dosage adjustments for carboplatin based on hepatic toxicity. Dose adjustments are for hepatotoxicity at least possibly related to paclitaxel or Talazoparib (BMN 673).

**AST and/or ALT****	**Talazoparib (BMN 673)**	**Paclitaxel**
Grade 1-2	No change	No change
Grade 3<br><br>Baseline 2.5-5x ULN and result <10x ULN<br><br>Baseline <2.5x ULN or result ≥ 10x ULN.	 <br>No change<br><br>Hold* until meeting treatment criteria and then reduce by 1 dose level.	 <br>No change<br><br>Hold* until meeting treatment criteria and then reduce by 1 dose level.
Grade 4	Off study	Off study
**Bilirubin****	**Talazoparib (BMN 673)**	**Paclitaxel**
Grade 1-2	No change	No change
Grade 3	Hold* until < Grade 2 and then reduce by 1 dose level.	Hold* until meeting treatment criteria and then reduce by 1 dose level.
Grade 4	Off study	Off study

Treatment criteria for paclitaxel includes: Bilirubin ≤1.25x ULN, with the exception of <2.9 mg/dL for patients with Gilbert's disease and AST/ALT ≤2.5x ULN (≤ 5x ULN in setting of metastatic liver disease)
* Patients requiring a delay of all protocol therapy >2 weeks due to toxicity should go off protocol therapy.
**Patients requiring > two dose reductions of chemotherapy should go off protocol therapy. Dose reductions are permitted for Talazoparib (BMN 673) until dose is 100mcg alone or in combination with chemotherapy.

If a toxicity leads to hold on day 1 of a cycle, the whole cycle is to be delayed until the toxicity meets treatment criteria. If a toxicity leads to a hold of paclitaxel during the cycle (i.e. day 8 or 15), the dose will be skipped and the dose modification will be with the next scheduled dose.

6.2.5 Hypersensitivity reaction

Caution: Patients who had a mild to moderate hypersensitivity reaction to paclitaxel and carboplatin have been successfully re-challenged, but careful attention to prophylaxis and bedside monitoring of vital signs is recommended.

Hypersensitivity reactions to paclitaxel and/or carboplatin will be managed as follows:
Mild symptoms (e.g., mild flushing, rash, pruritus) -Complete infusion. Supervise at bedside. No treatment required.

Moderate symptoms (e.g., moderate rash, flushing, mild dyspnea, chest discomfort) -Stop infusion. Give intravenous diphenhydramine 25 mg and intravenous dexamethasone 10 mg. Resume infusion after recovery of symptoms at a low rate, 20 mg/hr. For 15 minutes, then, if no further symptoms, at full dose rate until infusion is complete. If symptoms recur, stop infusion. The patient should receive no additional paclitaxel and/or carboplatin for that cycle, but may be retreated after discussion with the principal investigator. Record toxicity on flow sheets.

Severe life threatening symptoms (e.g., hypotension requiring pressor therapy, angioedema, respiratory distress requiring bronchodilation therapy, generalized urticaria)-stop infusion. Give intravenous diphenhydramine and dexamethasone as above. Add epinephrine or bronchodilators if indicated. If wheezing is present, that is not responsive to bronchodilators, epinephrine is recommended. Patient should be removed from further protocol therapy. Report as adverse event.

6.2.6 Other Toxicities

Grade 3 fatigue should be medically managed. If lasting more than 7 days, then all study treatment should be held until the fatigue recovers until grade 1 or less. The treatment may then be resumed at one dose level reduction of Talazoparib (BMN 673) and/or paclitaxel and carboplatin. If attribution for fatigue is at least possibly related to Talazoparib (BMN 673), this agent alone should be reduced by one dose level. If attribution for fatigue is unlikely or unrelated to Talazoparib (BMN 673), then the chemotherapy agent(s) with attribution of at least possible related should be reduced by one dose level.

Grade 3 or 4 depletion of electrolytes (e.g. K, Mg, Phos) should be optimally medically managed. If these persist for >48 hours despite attempts at repletion, then all study treatment should be held until the electrolytes return to grade 1 or less. The treatment may then be resumed at one dose level reduction of Talazoparib (BMN 673), paclitaxel and/or carboplatin, if attribution at least possibly related to drug.

For any grade 3 or 4 toxicity, not mentioned above, the treatment with the likely inciting agent should be withheld until the patient recovers to grade 1 or less toxicity. The treatment may then be resumed at one dose level reduction. For intolerable grade 2 toxicities, withhold treatment until the patient recovers, then resume treatment at a one dose level reduction.. Dose reduction will be done for the drug that is most likely to have caused the toxicity. For grade 1 or tolerable

grade 2 toxicities or clinically insignificant laboratory changes, no dose reduction should be made.

7 ADVERSE EVENTS: LIST AND REPORTING REQUIREMENTS

Adverse event (AE) monitoring and reporting is a routine part of every clinical trial. The following list of AEs (Section 7.1) and the characteristics of an observed AE (Section 7.2) will determine whether the event requires expedited reporting via the CTEP Adverse Event Reporting System (CTEP-AERS) **in addition** to routine reporting.

7.1 Comprehensive Adverse Events and Potential Risks List(s) (CAEPRs)

7.1.1 CAEPR for Talazoparib (MDV3800, BMN 673, NSC 771561)

The Comprehensive Adverse Events and Potential Risks list (CAEPR) provides a single list of reported and/or potential adverse events (AE) associated with an agent using a uniform presentation of events by body system. In addition to the comprehensive list, a subset, the Specific Protocol Exceptions to Expedited Reporting (SPEER), appears in a separate column and is identified with bold and italicized text. This subset of AEs (SPEER) is a list of events that are protocol specific exceptions to expedited reporting to NCI (except as noted below). Refer to the 'CTEP, NCI Guidelines: Adverse Event Reporting Requirements' http://ctep.cancer.gov/protocolDevelopment/electronic_applications/docs/aeguidelines.pdf for further clarification. *Frequency is provided based on 553 patients.* Below is the CAEPR for Talazoparib (MDV3800, BMN 673).

NOTE: Report AEs on the SPEER **ONLY IF** they exceed the grade noted in parentheses next to the AE in the SPEER. If this CAEPR is part of a combination protocol using multiple investigational agents and has an AE listed on different SPEERs, use the lower of the grades to determine if expedited reporting is required.

Version 2.4, October 24, 2020[1]

**Adverse Events with Possible Relationship to Talazoparib (MDV3800, BMN 673) (CTCAE 5.0 Term) [n= 553]**			**Specific Protocol Exceptions to Expedited Reporting (SPEER)**
**Likely (>20%)**	**Less Likely (<=20%)**	**Rare but Serious (<3%)**	
BLOOD AND LYMPHATIC SYSTEM DISORDERS			
Anemia			***Anemia (Gr 2)***
		Febrile neutropenia	
GASTROINTESTINAL DISORDERS			
	Abdominal pain		***Abdominal pain (Gr 2)***
	Constipation		***Constipation (Gr 2)***
Diarrhea			***Diarrhea (Gr 2)***
	Dyspepsia		
	Mucositis oral		
Nausea			***Nausea (Gr 2)***
Vomiting			***Vomiting (Gr 2)***
GENERAL DISORDERS AND ADMINISTRATION SITE CONDITIONS			
Fatigue			***Fatigue (Gr 2)***
	Fever		***Fever (Gr 2)***
	Pain		***Pain (Gr 2)***
INFECTIONS AND INFESTATIONS			
	Infection[2]		***Infection[2] (Gr 2)***
INVESTIGATIONS			

Adverse Events with Possible Relationship to Talazoparib (MDV3800, BMN 673) (CTCAE 5.0 Term) [n= 553]			Specific Protocol Exceptions to Expedited Reporting (SPEER)
**Likely (>20%)**	**Less Likely (<=20%)**	**Rare but Serious (<3%)**	
	Lymphocyte count decreased		
Neutrophil count decreased			***Neutrophil count decreased (Gr 4)***
Platelet count decreased			***Platelet count decreased (Gr 4)***
	White blood cell decreased		***White blood cell decreased (Gr 3)***
METABOLISM AND NUTRITION DISORDERS			
	Anorexia		***Anorexia (Gr 2)***
NEOPLASMS BENIGN, MALIGNANT AND UNSPECIFIED (INCL CYSTS AND POLYPS)			
		Leukemia secondary to oncology chemotherapy	
		Myelodysplastic syndrome	
		Treatment related secondary malignancy	
NERVOUS SYSTEM DISORDERS			
	Dizziness		***Dizziness (Gr 2)***
Headache			***Headache (Gr 2)***
SKIN AND SUBCUTANEOUS TISSUE DISORDERS			
Alopecia			***Alopecia (Gr 2)***

[1]This table will be updated as the toxicity profile of the agent is revised. Updates will be distributed to all Principal Investigators at the time of revision. The current version can be obtained by contacting PIO@CTEP.NCI.NIH.GOV. Your name, the name of the investigator, the protocol and the agent should be included in the e-mail.

[2]Infection may include any of the 75 infection sites under the INFECTIONS AND INFESTATIONS SOC.

[3]Neuropathy peripheral may include both Peripheral sensory neuropathy and Peripheral motor neuropathy under the NERVOUS SYSTEM DISORDERS SOC.

Adverse events reported on talazoparib (MDV3800, BMN 673) trials, but for which there is insufficient evidence to suggest that there was a reasonable possibility that talazoparib (MDV3800, BMN 673) caused the adverse event:

BLOOD AND LYMPHATIC SYSTEM DISORDERS - Blood and lymphatic system disorders - Other (pancytopenia)
CARDIAC DISORDERS - Atrial flutter; Sinus bradycardia
GASTROINTESTINAL DISORDERS - Abdominal distension; Flatulence; Intra-abdominal hemorrhage; Small intestinal obstruction; Toothache
GENERAL DISORDERS AND ADMINISTRATION SITE CONDITIONS - Edema limbs; General disorders and administration site conditions - Other (accidental overdose); Non-cardiac chest pain
HEPATOBILIARY DISORDERS - Hepatic failure; Sinusoidal obstruction syndrome
INVESTIGATIONS - Alanine aminotransferase increased; Aspartate aminotransferase increased; Weight loss
METABOLISM AND NUTRITION DISORDERS - Hyperkalemia; Hypokalemia
MUSCULOSKELETAL AND CONNECTIVE TISSUE DISORDERS - Arthralgia; Back pain; Generalized muscle weakness; Muscle cramp; Myalgia; Neck pain; Pain in extremity
NEOPLASMS BENIGN, MALIGNANT AND UNSPECIFIED (INCL CYSTS AND POLYPS) - Neoplasms

benign, malignant and unspecified (incl cysts and polyps) - Other (glioblastoma multiforme); Neoplasms benign, malignant and unspecified (incl cysts and polyps) - Other (metastases to meninges); Neoplasms benign, malignant and unspecified (incl cysts and polyps) - Other (metastatic breast cancer)
NERVOUS SYSTEM DISORDERS - Dysgeusia; Intracranial hemorrhage; Nervous system disorders - Other (neuropathy peripheral)[3]; Nervous system disorders - Other (nonserious axonal sensorimotor polyneuropathy); Syncope; Transient ischemic attacks
PSYCHIATRIC DISORDERS - Anxiety; Insomnia; Psychiatric disorders - Other (mental status changes)
RESPIRATORY, THORACIC AND MEDIASTINAL DISORDERS - Cough; Dyspnea; Epistaxis; Oropharyngeal pain; Pleural effusion; Respiratory, thoracic and mediastinal disorders - Other (obstructive airways disorder)
SKIN AND SUBCUTANEOUS TISSUE DISORDERS - Dry skin; Rash maculo-papular
VASCULAR DISORDERS - Thromboembolic event

Note: Talazoparib (MDV3800, BMN 673) in combination with other agents could cause an exacerbation of any adverse event currently known to be caused by the other agent, or the combination may result in events never previously associated with either agent.

7.1.2 Adverse Event List(s) for Commercial Agent(s)

Please refer to sections 8.2 and 8.3 for adverse events of carboplatin and paclitaxel.

7.2 Adverse Event Characteristics

- **CTCAE term (AE description) and grade:** The descriptions and grading scales found in the revised NCI Common Terminology Criteria for Adverse Events (CTCAE) version 4.0 will be utilized until March 31, 2018 for AE reporting. CTCAE version 5.0 will be utilized for AE reporting beginning April 1, 2018. All appropriate treatment areas should have access to a copy of the CTCAE version 5.0. A copy of the CTCAE version 5.0 can be downloaded from the CTEP web site http://ctep.cancer.gov/protocolDevelopment/electronic_applications/ctc.htm.

- **For expedited reporting purposes only:**
  - AEs for the agent that are ***bold and italicized*** in the CAEPR (*i.e.*, those listed in the SPEER column, Section 7.1.1) should be reported through CTEP-AERS only if the grade is above the grade provided in the SPEER.
  - Other AEs for the protocol that do not require expedited reporting are outlined in section 7.3.4.

- **Attribution** of the AE:
  - Definite – The AE *is clearly related* to the study treatment.
  - Probable – The AE *is likely related* to the study treatment.
  - Possible – The AE *may be related* to the study treatment.
  - Unlikely – The AE *is doubtfully related* to the study treatment.
  - Unrelated – The AE *is clearly NOT related* to the study treatment.

7.3 Expedited Adverse Event Reporting

7.3.1 Expedited AE reporting for this study must use CTEP-AERS (CTEP Adverse Event Reporting System), accessed via the CTEP Web site (https://eapps-ctep.nci.nih.gov/ctepaers). The reporting procedures to be followed are presented in the "NCI Guidelines for Investigators: Adverse Event Reporting Requirements for DCTD (CTEP and CIP) and DCP INDs and IDEs" which can be downloaded from the CTEP Web site: http://ctep.cancer.gov/protocolDevelopment/electronic_applications/adverse_events.htm. These requirements are briefly outlined in the tables below (Section 7.3.3).

In the rare occurrence when Internet connectivity is lost, a 24-hour notification is to be made to CTEP by telephone at 301-897-7497. Once Internet connectivity is restored, the 24-hour notification phoned in must be entered electronically into CTEP-AERS by the original submitter at the site.

7.3.2 CTEP-AERS is programmed for automatic electronic distribution of reports to the following individuals: Principal Investigator and Adverse Event Coordinator(s) (if applicable) of the Corresponding Organization, the local treating physician, and the Reporter and Submitter. CTEP-AERS provides a copy feature for other e-mail recipients.

The Coordinating Center of the Corresponding Organization is responsible for submitting to the CTSU documentation of AEs that they deem reportable for posting on the CTSU protocol web page and inclusion on the CTSU bi-monthly broadcast.

7.3.3 <u>Expedited Reporting Guidelines</u>

Use the NCI protocol number and the protocol-specific patient ID assigned during trial registration on all reports.

Note: A death on study requires <u>both</u> routine and expedited reporting regardless of causality. Attribution to treatment or other cause must be provided.

Death due to progressive disease should be reported as **Grade 5 "Disease progression"** in the system organ class (SOC) "General disorders and administration site conditions". Evidence that the death was a manifestation of underlying disease (*e.g.*, radiological changes suggesting tumor growth or progression: clinical deterioration associated with a disease process) should be submitted.

7.3.4 Additional Protocol-Specific Expedited Adverse Event Reporting Exclusions

For this protocol only, the AEs/grades listed below do not require expedited reporting via CTEP-AERS. However, they still must be reported through the routine reporting mechanism (Section 7.4):

CTCAE SOC	Adverse Event	Grade	Hospitalization/ Prolongation of Hospitalization	Attribution
Nervous system	Peripheral motor or sensory neuropathy	2	<24 hours	Possibly or greater
General disorders	Fatigue	2	<24 hours	Possibly or greater
Gastrointestinal disorders	Nausea	3	<24 hours	Possibly or greater
Gastrointestinal disorders	Vomiting	3	<24 hours	Possibly or greater

Phase 1 and Early Phase 2 Studies: Expedited Reporting Requirements for Adverse Events that Occur on Studies under an IND/IDE within 30 Days of the Last Administration of the Investigational Agent/Intervention [1, 2]

FDA REPORTING REQUIREMENTS FOR SERIOUS ADVERSE EVENTS (21 CFR Part 312)

NOTE: Investigators **MUST** immediately report to the sponsor (NCI) **ANY** Serious Adverse Events, whether or not they are considered related to the investigational agent(s)/intervention (21 CFR 312.64)

An adverse event is considered serious if it results in **ANY** of the following outcomes:

1) Death
2) A life-threatening adverse event
3) An adverse event that results in inpatient hospitalization or prolongation of existing hospitalization for ≥ 24 hours
4) A persistent or significant incapacity or substantial disruption of the ability to conduct normal life functions
5) A congenital anomaly/birth defect.
6) Important Medical Events (IME) that may not result in death, be life threatening, or require hospitalization may be considered serious when, based upon medical judgment, they may jeopardize the patient or subject and may require medical or surgical intervention to prevent one of the outcomes listed in this definition. (FDA, 21 CFR 312.32; ICH E2A and ICH E6).

ALL SERIOUS adverse events that meet the above criteria MUST be immediately reported to the NCI via electronic submission within the timeframes detailed in the table below.

Hospitalization	Grade 1 and Grade 2 Timeframes	Grade 3-5 Timeframes

<table>
<tr><td>Resulting in Hospitalization ≥ 24 hrs.</td><td>10 Calendar Days</td><td rowspan="2">24-Hour 5 Calendar Days</td></tr>
<tr><td>Not resulting in Hospitalization ≥ 24 hrs.</td><td>Not required</td></tr>
<tr><td colspan="3">NOTE: Protocol specific exceptions to expedited reporting of serious adverse events are found in the Specific Protocol Exceptions to Expedited Reporting (SPEER) portion of the CAEPR.

<u>Expedited AE reporting timelines are defined as:</u>
o “24-Hour; 5 Calendar Days” - The AE must initially be submitted electronically within 24 hours of learning of the AE, followed by a complete expedited report within 5 calendar days of the initial 24-hour report.
o “10 Calendar Days” - A complete expedited report on the AE must be submitted electronically within 10 calendar days of learning of the AE.</td></tr>
<tr><td colspan="3">[1]Serious adverse events that occur more than 30 days after the last administration of investigational agent/intervention and have an attribution of possible, probable, or definite require reporting as follows:
Expedited 24-hour notification followed by complete report within 5 calendar days for:
• All Grade 3, 4, and Grade 5 AEs
Expedited 10 calendar day reports for:
• Grade 2 AEs resulting in hospitalization or prolongation of hospitalization

[2]For studies using PET or SPECT IND agents, the AE reporting period is limited to 10 radioactive half-lives, rounded UP to the nearest whole day, after the agent/intervention was last administered. Footnote “1” above applies after this reporting period.

Effective Date: May 5, 2011</td></tr>
</table>

7.4 Routine Adverse Event Reporting

All Adverse Events **must** be reported in routine study data submissions. **AEs reported expeditiously through CTEP-AERS must <u>also</u> be reported in routine study data submissions.**

7.5 Secondary Malignancy

A *secondary malignancy* is a cancer caused by treatment for a previous malignancy (*e.g.*, treatment with investigational agent/intervention, radiation or chemotherapy). A secondary malignancy is not considered a metastasis of the initial neoplasm.

CTEP requires all secondary malignancies that occur following treatment with an agent under an NCI IND/IDE be reported expeditiously via CTEP-AERS. Three options are available to describe the event:

- Leukemia secondary to oncology chemotherapy (*e.g.*, acute myelocytic leukemia [AML])
- Myelodysplastic syndrome (MDS)
- Treatment-related secondary malignancy

Any malignancy possibly related to cancer treatment (including AML/MDS) should also be reported via the routine reporting mechanisms outlined in each protocol.

7.6 Second Malignancy

A second malignancy is one unrelated to the treatment of a prior malignancy (and is **NOT** a metastasis from the initial malignancy). Second malignancies require **ONLY** routine AE reporting unless otherwise specified.

8 PHARMACEUTICAL INFORMATION

A list of the adverse events and potential risks associated with the investigational or commercial agents administered in this study can be found in Section 7.1.

8.1 CTEP IND Agent: Talazoparib (BMN 673) (NSC # 771561)

Chemical Name: 3*H*-Pyrido [4,3,2-de]phthalazin-3-one, 5-fluoro-8-(4-fluorophenyl)-2,7,8,9-tetrahydro-9-(1-methyl-1*H*-1,2,4-triazol-5-yl)-, (8***S***,9***R***)-, 4-methylbenzenesulfonate (1:1)

Other Names: Talazoparib (BMN 673), BMN 673ts, talazoparib

Classification*:* poly (ADP-ribose) polymerase (PARP) inhibitor

CAS: 1207456-01-6

Molecular Formula: $C_{26}H_{22}F_2N_6O_4S$ (BMN 673ts) **M.W.:** 552.5624 (BMN 673ts)

Mode of Action: Talazoparib (BMN 673) is a potent and specific inhibitor of PARP1 and PARP2, preventing PARP-mediated DNA repair of single strand DNA breaks via the base-excision repair pathway. It has demonstrated synthetic lethality in tumors with defects in DNA repair pathways, such as BRCA mutations and PTEN dysfunction.

Description: Talazoparib (BMN 673) free base is the active moiety of the Talazoparib (BMN 673)ts (tosylate salt) formulation.

How Supplied: Talazoparib (BMN 673) capsules are supplied by Pfizer, Inc., and distributed by the Pharmaceutical Management Branch, CTEP/DCTD/NCI. Talazoparib (BMN 673) is supplied as 100 mcg (opaque ivory, size 4) and 250 mcg capsules (opaque white, size 4) packaged in 30-count HDPE bottles with an induction seal and child-resistant cap. The hypromellose capsules contain a blend of Talazoparib (BMN 673) drug substance, silicified microcrystalline cellulose, titanium dioxide, red iron oxide, and yellow iron oxide.

Talzoparib (BMN 673) capsules may be repackaged from the manufacturer-supplied HDPE bottle into a pharmacy-supplied HDPE bottle for dispensing purposes.

Storage: Store Talazoparib (BMN 673) 100 mcg capsules at room temperature (15-25°C/59-77°F) and protected from light. Store Talazoparib (BMN 673) 250 mcg capsules at room temperature (15-30°C/59-86°F) and protected from light.

If a storage temperature excursion is identified, promptly return Talazoparib (BMN 673) to between 15-25°C (100 mcg) or 15-30°C (250 mcg) and quarantine the supplies.. Provide a detailed report of the excursion (including documentation of temperature monitoring and duration of the excursion) to PMBAfterHours@mail.nih.gov for determination of suitability.

Stability: Shelf-life stability studies of Talazoparib (BMN 673) capsules are ongoing.

Route of Administration: Oral administration. Take Talazoparib (BMN 673) with or without food.

Potential Drug Interactions: Based on in vitro data, Talazoparib (BMN 673) is not likely to demonstrate clinically significant CYP450 inhibition- or induction-based drug-drug interactions. Talazoparib (BMN 673) is a substrate for P-glycoprotein (P-gp) and breast cancer resistance protein (BCRP) drug transporters. The PK parameters of Talazoparib (BMN 673) could be altered if Talazoparib (BMN 673) is coadministered with P-gp and BCRP inhibitors/inducers. Studies have shown that Talazoparib (BMN 673) is not a substrate or an inhibitor of OATP1B1, OATP1B3, OCT1, OCT2, OAT1, OAT3, MATE1, or BSEP transporters.

Availability: Talazoparib (BMN 673) is an investigational agent supplied to investigators by the Division of Cancer Treatment and Diagnosis (DCTD), NCI.

Talazoparib (BMN 673) is provided to the NCI under a Collaborative Agreement between the Pharmaceutical Collaborator and the DCTD, NCI (see Section 12.3).

8.1.1 **Agent Ordering, Accountability, and Investigator Brochure Availability**

8.1.1.1 NCI-supplied agents may be requested by eligible participating Investigators (or their authorized designee) at each participating institution. The CTEP-assigned protocol number must be used for ordering all CTEP-supplied investigational agents. The eligible participating investigators at each participating institution must be registered with CTEP, DCTD through an annual submission of FDA Form 1572 (Statement of Investigator), NCI Biosketch, Agent Shipment Form, and Financial Disclosure Form (FDF). If there are several participating investigators at one institution, CTEP-supplied investigational agents for the study should be ordered under the name of one lead participating investigator at that institution.

In general, sites may order initial agent supplies when a subject is being screened for enrollment onto the study.

Active CTEP-registered investigators and investigator-designated shipping designees and ordering designees can submit agent requests through the PMB Online Agent Order Processing (OAOP) application. Access to OAOP requires the -establishment of a CTEP Identity and Access Management (IAM) account and the maintenance of an “active” account status and a “current” password. For questions about drug orders, transfers, returns, or accountability, call or email PMB any time. Refer to the PMB’s website for specific policies and guidelines related to agent management.

8.1.1.2 Agent Inventory Records – The investigator, or a responsible party designated by the investigator, must maintain a careful record of the receipt, dispensing and final disposition of all agents received from the PMB using the appropriate NCI Investigational Agent (Drug) Accountability Record (DARF) available on the CTEP forms page. Store and maintain separate NCI Investigational Agent Accountability Records for each agent, strength, formulation and ordering investigator on this protocol.

8.1.1.3 Investigator Brochure Availability

The current version of the Investigator Brochure (IB) will be accessible to site investigators and research staff through the PMB Online Agent Order Processing (OAOP) application. Access to OAOP requires the establishment of a CTEP Identity and Access Management (IAM) account and the maintenance of an "active" account status and a "current" password. Questions about IB access may be directed to the PMB IB coordinator via email.

8.1.1.4 Useful Links and Contacts:

CTEP Forms, Templates, Documents: http://ctep.cancer.gov/forms/
NCI CTEP Investigator Registration: RCRHelpDesk@nih.gov
PMB policies and guidelines: http://ctep.cancer.gov/branches/pmb/agent_management.htm
PMB Online Agent Order Processing (OAOP) application: https://ctepcore.nci.nih.gov/OAOP
CTEP Identity and Access Management (IAM) account: https://
ctepcore.nci.nih.gov/iam/index.jsp
CTEP Associate Registration and IAM account help: ctepreghelp@ctep.nci.nih.gov
PMB email: PMBAfterHours@mail.nih.gov
IB Coordinator: IBCoordinator@mail.nih.gov
PMB phone and hours of service: (240) 276-6575 Monday through Friday between 8:30 am and 4:30 pm (ET)

8.2 Commercial Agent: Paclitaxel

Availability

Paclitaxel is commercially available. Paclitaxel is a natural product obtained via a semi- synthetic process from Taxus baccata. Improved solubility requires a mixed solvent system with further dilutions of either 0.9% sodium chloride (NS) or 5% dextrose in water. Paclitaxel is supplied in a sterile concentrated solution, 6 mg/mL, and is available in 5 mL (30 mg) and 16.7 mL (100 mg) multidose vials. Each milliliter contains 6 mg Taxol, 527 mg of "Cremophor" EL (polyoxyethylated caster oil) and 50% dehydrated alcohol, USP, 50%. The contents of the vial must be diluted just prior to clinical use. Commercial supplies of Taxol will be used for this study.

Solution Preparation

Paclitaxel must be diluted prior to infusion. Paclitaxel should be diluted in 0.9% sodium chloride or 5% dextrose injection to a final concentration of 0.3 to 1.2 mg/mL. Dilution and infusion volumes may be modified per local institutional standards. Infusions should be mixed as closely as possible to the start of each infusion since the stability of diluted Paclitaxel after 27 hours at room temperature is unknown. Paclitaxel must be prepared in glass, polypropylene, or polyolefin container due to leaching of di-(2-ethylhexyl)- phthalate (DEHP) plasticizer from polyvinyl chloride (PVC) bags. Paclitaxel will be administered using non-PVC tubing and connectors such as the I.V. administration sets which are polyethylene lined. In-line filtration must be accomplished by incorporating a hydrophilic, microporous filter of pore size not greater than

0.22 microns (e.g., IVEX- HP and IVEX-II, Abbott Laboratories). Nothing else is to be infused through the lines where Paclitaxel is being administered. Solutions exhibiting excessive particulate formation should be discarded.

Storage and Stability

Intact vials should be stored at room temperature (between 2-25°C or 36-77°F). Shelf- life of the vials stored under appropriate conditions corresponds to the manufacturer's expiration date on each vial. All solutions of Paclitaxel exhibit a slight haziness directly proportional to the concentration of drug and time elapsed since preparation. When prepared as described above, solutions of Paclitaxel (0.3 – 1.2 mg/ml) are stable for 27 hours at room temperature.

Calculating Dosage of Paclitaxel

The dosage will be calculated each cycle based on the patient's surface area using the patient's actual weight at the time. The dosage will be rounded per institutional guidelines. In calculating surface areas, actual heights and weights should be used, i.e., there will be no adjustment to "ideal" weight.

Administration of Paclitaxel

Paclitaxel, at the appropriate dose and dilution, will be given as a one hour IV infusion. Paclitaxel will be administered using non-PVC bag, tubing and connectors. It must be filtered using a 0.22 micron in line non-DEHP placed on the distal end of the infusion line. Nothing else is to be infused through the line where Paclitaxel is being administered.

Adverse Effects

The following adverse events are expected with the administration of Paclitaxel. For complete information, see Package Insert.

Myelosuppression, nausea and vomiting, diarrhea, stomatitis, mucositis

Arrhythmia, heart block, ventricular tachycardia, hypotension, myocardial infarction (MI)

Peripheral neuropathy, seizures

Anaphylactoid and urticarial reactions (acute), flushing, rash, pruritus

Alopecia, malaise, arthralgia, myalgia, elevated AST, alkaline phosphatase and bilirubin.

Note: Cardiac toxicities are rare and continuous cardiac monitoring is not required except for patients with serious conduction abnormalities or other underlying, serious cardiac risk factors

<u>Recommended Management of Hypersensitivity Reactions</u>

Mild Symptoms: (i.e., mild flushing pruritus) Complete Paclitaxel infusion. No treatment required.

Moderate symptoms: (i.e., moderate rash, flushing, mild dyspnea, chest discomfort)

Stop Paclitaxel administration. Give intravenous dexamethasone 10 mg and diphenhydramine HCl 25 mg. After recovery from symptoms, resume Paclitaxel infusion at 20 mL/hr for 15 minutes, then 50 mL/hr for 15 minutes, then if no further symptoms, at full dose rate until infusion is complete. If symptoms recur, stop Paclitaxel infusion. The patient should not receive additional Paclitaxel for that week but may be retreated after discussion with the Principal Investigator.

Severe symptoms: (i.e., hypotension requiring pressor therapy, IV fluids, angioedema, respiratory distress requiring bronchodilation therapy, generalized urticaria).

Stop Paclitaxel administration. Give intravenous dexamethasone and diphenhydramine HCl 25 mg as above. Add adrenaline (1:1000) or bronchodilators as indicated. Report the event as a serious adverse event (SAE) (see Section 7.2). If severe symptoms occur, the patient should not receive additional Paclitaxel and should be taken off of the study.

8.3 Commercial Agent: Carboplatin Availability

Carboplatin is commercially available in a variety of vial sizes.

Preparation

Dilution and infusion volumes may be modified per local institutional standards. Consult the package insert for specific instructions.

Storage and Stability

When prepared as directed, the resultant carboplatin solutions, when protected from light, are stable for 8 hours at room temperature. No antibacterial preservative is contained in the formulation, and therefore, it is recommended that carboplatin solutions be discarded 8 hours after dilution.

NOTE: Aluminum reacts with carboplatin, causing precipitate formation and loss of potency. Therefore, needles or intravenous sets containing aluminum parts that may come in contact with the drug must not be used for the preparation or administration of carboplatin.

Unopened vials of carboplatin are stable for the life indicated on the package when stored at controlled room temperature (59 - 86˚F) and protected from light.

Dosing and Administration

Carboplatin will be administered after Paclitaxel as an IV infusion over 30 minutes in Sodium Chloride 0.9% or Dextrose 5% in water per institutional standard. The dose will be calculated based on the patient’s actual body weight at each treatment visit and the AUC (area under curve) dosing according to the formula provided in the treatment plan above.

Toxicities-Some of the expected adverse events from carboplatin are listed below. For further description of adverse events see Package Insert.

Myelosuppression, nausea, vomiting, diarrhea, weight loss, constipation, gastrointestinal pain

Electrolyte imbalances, hypomagnesemia, hypocalcemia, hyponatremia, hyperuremia Elevated

alkaline phosphatase, AST, and total bilirubin

Peripheral neuropathies (mild paresthesias, clinical ototoxicity and other sensory abnormalities are rare)

Renal tubular damage, renal insufficiency, impotence, sterility, amenorrhea, gynecomastia Anaphylactoid and urticarial reactions (acute), flushing, rash, pruritis and rarely hypotension or bronchospasm

Alopecia, pain, asthenia and mucosal side effects, decreased serum electrolytes values (sodium, magnesium, calcium and potassium)

9 BIOMARKER, CORRELATIVE, AND SPECIAL STUDIES

9.1 Integral Laboratory or Imaging Studies: N/A

9.2 Investigational Device Information: N/A

9.3 Integrated Correlative Studies: N/A

9.4 Exploratory/Ancillary Correlative Studies (Portions dependent on securing adequate funding).

All samples collected for research will be coded without any patient identifiers. All samples will be destroyed and the completion of this study and its analysis. A laboratory manual will be provided in conjunction with this protocol with further details regarding specimen handling and processing.

9.4.1 **Pharmacokinetics:** (no additional funding needed) The plasma pharmacokinetics of Talazoparib (BMN 673) will be evaluated as a potential marker of toxicity, specifically thrombocytopenia as well as correlated with PD changes observed in PBMCs by an indirect PK/PD model. On each day with PK or PD collection time points, Talazoparib (BMN 673) will be dosed in clinic (prior to paclitaxel and carboplatin administration, if applicable). Plasma samples for Talazoparib (BMN 673) pharmacokinetics will be collected prior to Talazoparib (BMN 673) dosing and approximately 4 hours post Talazoparib (BMN 673) dose to assess Cmax on Day 1 of Cycle 1. Samples will again be collected 4 hours post Talazoparib (BMN 673) dose on Cycle 1 Day 3 or 7 (the last day of Talazoparib (BMN 673) depending on assigned schedule). On Cycle 2, Day 1 samples will be collected at time 0 and 4 hours post Talazoparib (BMN 673) dose For cycle 3 and beyond, Talazoparib (BMN 673) PK will be drawn at time 0 and 4 hours post dose on day 1 only on each cycle coinciding with a radiographic assessment through the 5th cycle.

Cmax and Day 3 (or 7) concentrations will be correlated with thrombocytopenia and pharmacodynamic changes in PBMCs (described in section 9.4.2) and may be useful in subsequent studies to achieve levels required to maintain PARP inhibition while minimizing toxicity. Plasma concentrations of Talazoparib (BMN 673) will be evaluated by a validated LCMSMS assay and done in the 3P laboratory at the UWCCC. Data obtained with this sparse sampling will be combined with PK data from other phase I studies of the agent, if available, to develop a population model of Talazoparib (BMN 673) pharmacokinetics as previously described.[76]

9.4.1.1 Collection of Specimen(s) In both schedules, samples for Talazoparib (BMN 673) pharmacokinetics will be collected using the following schedule:

Dose Escalation Cohorts A and B: PK Collection Timetable

Day/Time (hr)	Talazoparib (BMN 673)
**C1D1**	
0 (prior to first Talazoparib (BMN 673) dose)	X
4 hr. post	X
**C1D3 or C1D7**[a]	
4 hr. post	X
**C2D1**	
0 (prior to first Talazoparib (BMN 673) dose)	X
4 hr. post	X
**CND1**[b]	
0 (prior to first Talazoparib (BMN 673) dose)	X
4 hr. post	X

a. On last day of Talazoparib (BMN 673) in cycle 1 per assigned schedule
b. To be collected on cycles when radiologic assessment for disease evaluation is being performed through the 5th cycle. No longer collected after Cycle 5.

9.4.1.2 Handling of Specimens(s) A 6mL sample of blood will be collected at the time points described in study calendar. The sample will be processed and plasma stored at -70C until analysis. Details of the sample collection, processing, storage and analysis will be described in the laboratory manual.

9.4.1.3 Shipping of Specimen(s)

PK specimens to be shipped to the UWCCC 3P lab at the following address:

3P Laboratory
University of Wisconsin-Carbone Cancer Center 600
Highland Avenue K6/570
Madison, WI 53792.

Samples may be batch shipped quarterly.

Call the 3P laboratory at 608-263-5369 or email 3plab@lists.medicine.wisc.edu to set-up a shipment time. Samples should be shipped to arrive M-F and not on a holiday.

Analysis will be conducted in the 3P analytical laboratory. The 3P lab is a core lab of the UWCCC and has extensive experience in the development of analytical methods

and sample analysis.

9.4.1.4 Site(s) Performing Correlative Study: All sites

9.4.2 **Pharmacodynamic Evaluation** (pending adequate funding-samples will be collected, but not analyzed until confirmation of funding)

PBMCs will be evaluated for evidence of DNA damage from baseline to the completion of Talazoparib (BMN 673) during cycle 1 of therapy. Planned assays include the previously validated tests for induction of γH2AX and presence of RAD51 foci, and PAR inhibition.[61, 66, 77, 78] Samples will be obtained prior to dosing on day 1 of cycle 1 and again on the last day of Talazoparib (BMN 673) therapy in cycle 1 (day 7 for schedule A, day 3 for schedule B). They will also be collected on day 1 of cycle 2. PBMC analysis for induction of γH2AX, presence of RAD51 foci, and PAR inhibition will be performed in the 3P laboratory at the UWCCC.

9.4.2.1 Collection of Specimen(s) In both cohorts, samples for pharmacodynamics will be collected using the following schedule:

PBMC Collection Timetable

Day/Time (hr)	PBMC
C1D1/time 0 (prior to first Talazoparib (BMN 673) dose)<br>C1D 3 or 7[a]/time 4 hrs. after Talazoparib (BMN 673) dose)	X<br>X
C2D1/ time 4 hrs. after Talazoparib (BMN 673) dose	X

a. On last day of Talazoparib (BMN 673) in cycle 1 per assigned schedule

9.4.2.2 Handling of Specimens(s)

PBMCs: Two 8mL (16mL total) blood samples will be drawn into a CPT tube at the above time points. After collection, store the tube upright at room temperature. Centrifuge at 1500 x g for 15 minutes at room temperature to obtain PBMCs. Aspirate approximately half of the plasma without disturbing the cell layer. Collect cell layer with a Pasteur pipette and transfer to a 15 mL size conical centrifuge tube with cap.

9.4.2.3 Shipping of Specimen(s)

PBMC specimens to be shipped to the UWCCC 3P lab at the following address:

3P Laboratory
University of Wisconsin-Carbone Cancer Center 600

Highland Avenue K6/570
Madison, WI 53792.

Samples may be batch shipped quarterly.

Call the 3P laboratory at 608-263-5369 or email 3plab@lists.medicine.wisc.edu to set-up a shipment time. Samples should be shipped to arrive M-F and not on a holiday.

PBMC analysis will be conducted in the 3P analytical laboratory. The 3P lab is a core lab of the UWCCC and has extensive experience in the development of analytical methods and sample analysis.

9.4.2.4 Site(s) Performing Correlative Study: All sites

9.4.3 Mechanisms of Resistance

Tumor tissue will be evaluated for potential mechanisms of resistance to therapy. Patients will be asked to consent for submission of baseline archived tumor tissue. A tumor block will be preferred over unstained slides and tissue from the most recent metastatic site biopsy is preferred over primary tumor. Non-bone sites are preferred to bone tissue samples.

In addition, patients on study who meet all criteria below will be asked to undergo an optional standard of care tumor biopsy at the time of disease progression on study:

- Signed optional consent for obtaining tumor tissue for genomic analysis
- Archived tumor tissue available
- Responding to study treatment: This will include patients with measurable disease at baseline with documented PR or CR as defined in section 11 as well as those patient with non-measurable, but evaluable, disease at baseline who the treating physician reports having a clinical benefit (i.e. decline in tumor marker or decrease of non-measurable disease burden).
- In addition, patients who meet criteria above must also either a) remain on study treatment at time of disease progression OR b) have progressive disease within 3 months of stopping study treatment and without interval initiation of a different anti-cancer therapy.

It is estimated that 20 patients will meet these criteria.

The archived baseline tumor tissue will be a research sample for all patients. The tumor biopsy after progression is a standard of care procedure. For patients with a biopsy upon progression and a baseline archived tumor sample, both samples will undergo genomic sequencing using a next generation sequencing (such as Foundation One® Panel through Foundation Medicine). The biopsy upon progression genomic sequencing will be coordinated from the participant's site using a research agreement between that site and a vendor or commercial testing with costs covered by the UM1.

Current research agreements between Foundation Medicine and the UWCCC will be used to

cover the costs of this CLIA certified test at the UWCCC if external funding is not secured. This research agreement between UWCCC and Foundation Medicine will also be used for all archived tumor samples from all sites. Since the results from the genomic sequencing from the biopsy upon progression could have clinical treatment implications, this specimen will be sent out immediately after the biopsy and results (from this sample only, not the archived tissue) will be provided to the treating physician. For those participants who had previous genomic sequencing on a tumor specimen (primary or metastastic) on a tumor sample obtained prior to study enrollment, the data from the results of these genomic studies may be collected if the baseline archived tumor tissue is not able to be collected or assay failure occurs.

If funding is obtained, further analysis including mRNA expression by RT-PCR, RNA Seq and protein expression by immunohistochemistry (IHC) specifically of BRCA1, BRCA2, 53BP1, RIF1 and PTIP will also be performed on both biopsy samples. The aim will be to evaluate the baseline status of these proteins and whether changes are associated with either primary resistance or the development of acquired resistance. As a complementary unbiased approach, if adequate tissue and funding is available, full exome sequencing and transcriptome analysis will be done on paired samples to look for potential mechanisms of resistance.

If additional funding is secured, samples from tumors that show progression after response will also be used to generate primary patient-derived xenografts (PDX) models. The PDX models will be characterized both genomically and functionally to determine the role of any novel genomic alterations in contributing to resistance to Talazoparib (BMN 673), platinum and/or taxanes. If PDX models are pursued, the protocol will be amended to include further details.

9.4.3.1 Collection of specimens

All subjects will have archived tumor tissue confirmed to be available at time of study enrollment. Subsequently, a tumor block (preferred) or at least 15 unstained slides will be obtained. Metastatic tumor tissue is preferred over primary tumor samples. Non-bone sites are preferred over bone tumor samples.

A repeat tumor biopsy will be performed at the time of disease progression in those patients meeting criteria described in section 9.4.3 as long as they are deemed low risk for complications by the treating provider. Schema for tumor tissue acquisition:

Pt with advanced solid tumor eligible for study

↓

Archived tumor tissue available
Signed consent for tumor tissue for genomic analysis

↓

Start treatment (using dose escalation table)

↓	↓	↓
Complete or partial response or clinical benefit	Progressive disease	Stable disease
↓	↓	↓
Biopsy at the time of progression if pt continues on therapy or progressive disease within 3 months of stopping study treatment without interval initiation of a different therapy	No biopsy at the time of progression	No biopsy at the time of progression

*Patients who are not evaluable or those stopping study before a disease evaluation for toxicity or MD discretion will not be eligible for biopsy.

9.4.3.2 Handling of Specimens-(Details regarding preparation of tumor samples and shipping information will be included in the laboratory manual).

Baseline archived tumor blocks or unstained slides (fresh tumor biopsies are allowed per discretion of the treating physician) will be prepared by a pathologist at the treating institution. Upon progression, the fresh tumor biopsy will be considered a standard of care procedure as this has become the approach for the majority of solid tumor malignancies.

Four core biopsies are recommended, two for standard of care and two for research purposes. These can be flash frozen or formalin fixed paraffin embedded (FFPE). The research samples should be held until confirmation is obtained from pathology that there is adequate tumor tissue for standard of care testing. Holding of specimens can be done by an approved BioBank. If at that time, the research specimens are deemed to have inadequate tumor tissue, an archived tumor block or 15 unstained slides should be requested from the standard of care clinical specimen as long as this can be accomplished without exhausting the this tumor block.

The tumor samples (samples from biopsy at time of progression) will then be prepared for shipment. After tumor samples are prepared at each study site, they will be sent to Foundation Medicine for Foundation One® analysis or to another genomic sequencing company depending on the site's research agreement. (contact the lead site for further instruction). The archived baseline tumor tissue samples will be used for research purposes only and will be batched to prepare for shipment to Foundation Medicine for Foundation One® analysis.

Any additional tumor tissue remaining will be stored for future studies (described in section 9.4.3 above).

9.4.3.3 Shipping of Specimens

For the subset of subjects undergoing a biopsy upon disease progression, the research tumor samples (samples from biopsy at time of progression) will be prepared for shipment to Foundation Medicine for Foundation One® analysis. Biopsies from time of tumor progression should ideally be sent to Foundation Medicine within 2 weeks from biopsy. The results from Foundation One® analysis will be linked to the patient and provided to the treating physician as well to be used to make clinical treatment decisions per standard institutional approach. If concern for a hereditary cancer syndrome is identified, as clinically indicated, genetic counseling may be advised.

The research only tumor samples (archived baseline tumor samples) will be coded with a study ID number and batch shipped to Foundation Medicine for Foundation One® analysis throughout the course of the study.

Any additional tumor tissue remaining will be stored for future studies (described in section 9.4.3 above). These may be batch shipped quarterly. At the completion of analysis, all tumor blocks will be returned if requested by the originating institution and any remaining tumor samples destroyed.

All subjects enrolled at UWCCC will have tumor tissue specimens shipped to and stored at the UWCCC TRIP-(Translational Research Initiatives in Pathology) lab at the following address:

UWCCC-TRIP lab
c/o Sally Ann Drew, BS, MT
600 Highland Avenue
L5/181
Madison, WI 53792
trip@pathology.wisc.edu

All subjects enrolled at OSU will have tumor tissue specimens shipped to and stored at the address listed below.

OSU Wexner Medical Center
Pathology/Tissue Archive Service
410 West 10th Ave.
E419 Doan
Columbus, OH 43210

Attn: Cheryl Reeder (tel: 614-293-7355; email: Cheryl.reeder@osumc.edu) AND/OR
Robert Wesolowski, MD (tel: 614-366-8541; email: Robert.Wesolowski@osumc.edu)

All subjects enrolled at CINJ will have tumor tissue specimens shipped to and stored at the address listed below.

Also, extra tumor blocks/slides (after initial sequencing) may be shipped from the UWCCC or OSU to CINJ for further analyses as described above. The laboratory address is:

Rutgers Cancer Institute of New Jersey-Biospecimen Repository Service (BRS)
Attention: Novlette Simmons
195 Little Albany St., Room 2031
New Brunswick, NJ 08901
Email: simmonno@cinj.rutgers.edu

The protocol will be amended if patient derived xenografts from biopsies upon progression are funded.

9.4.3.4 Sites participating in correlative study: All sites

All analysis will be done on de-identified tumor tissue. Each sample will be given a code prior to analysis. The Ganesan laboratory (R-CINJ) has experience in analyzing components of the DNA repair pathway, and in clinical specimens.

9.5 Special Studies: N/A

10 STUDY CALENDAR

A. Study Calendar for carboplatin, paclitaxel and Talazoparib (BMN 673) combination therapy Baseline evaluations are to be conducted within 1 week (7 days) prior to start of protocol
therapy. Scans and x-rays must be done <u><</u>4 weeks (28 days) prior to the start of therapy. In the event that the patient's condition is deteriorating, laboratory evaluations should be repeated within 48 hours prior to initiation of the next cycle of therapy.

	Pre-Study[c]	C1D1[d]	C1D8 +/- 1d	C1D15 +/- 1d	CND1 +/- 3d	CND8 +/- 1d	CND15 +/- 1d	Disease Progression[e] + 3 weeks	Off Study[f]
Talazoparib (BMN 673)		A			A				
Carboplatin		B			B				
Paclitaxel		C	C	C	C	C	C		
Informed consent	X								
Demographics	X								
Medical history	X								
Concurrent meds	X	X -- X							
Physical exam	X	X[l]			X				X
Vital signs	X	X			X				X
Height	X								
Weight	X				X				X
Performance status	X	X			X				X
CBC w/diff, plts[j, k]	X	X	X	X	X	X	X		X
Serum chemistry[a,j,k]	X	X	X	X	X				X
Adverse event evaluation	X	X -- X							X
B-HCG	X[b]								
Tumor Measurements	X	Tumor measurements are repeated every 6 weeks (+/- 1 week). Documentation (radiologic) must be provided for patients removed from study for progressive disease. [g]							X
Radiologic evaluation	X	Radiologic measurements should be performed every 6 weeks (+/- 1 week).[g]							X
Tumor Tissue (Archived and upon progression)[i]	X								
Other correlative studies (see section 9 for detail about PK and PBMC collection time points)		X[h]							
Tumor Biopsy[i]								X	

	A: Talazoparib (*BMN 673): Dose as assigned; administration schedule oral once daily for either 3 or 7 days based on assigned cohort*
	B: *Carboplatin:* Dose as assigned*; administration schedule once iv on day 1 of each cycle*
	C: *Paclitaxel:* Dose as assigned*; administration schedule once iv on days 1, 8, 15 of each cycle.*
	a: Albumin, alkaline phosphatase, total bilirubin, bicarbonate, BUN, calcium, chloride, creatinine, glucose, LDH, phosphorus, potassium, total protein, SGOT [AST], SGPT [ALT], sodium.
	b: Serum pregnancy test (women of childbearing potential).
	c: Pre-study evaluations must be done within 7 days prior to C1D1 with the exception of Radiologic evaluations which should be performed within 28 days prior to C1D1.
	d: C1D1 labs need to meet treatment parameters described in section 6.0 and will be used to dose study therapy. If there is a significant change from screening laboratory results, a discussion with PI or study chair is encouraged before starting study drugs. Labs done for screening may be used for C1D1 if done within +/- 72 hours.
	e: At time of disease progression, repeat tumor biopsy for all patients with RECIST defined clinical response or those with evaluable disease with clinician defined response (see section 9.0)
	f. Off-study evaluations
	g. Preferred radiologic measurements include CXR, CT chest, CT or MRI abdomen, CT or MRI pelvis, bone scan. PET/CT may be used in appropriate cases with PI approval. After 4 cycles of study therapy, tumor and radiologic measurements can be spaced to every 6-12 weeks. For patients on study therapy for over 18 months, radiologic tumor measurements can be repeated less frequently, per MD discretion, but no longer than every 6 months.
	h. Pharmacokinetic samples will be collected C1D1, C1D3 or C1D7 (depending on schedule), C2D1 and for cycle 3 and beyond only on day 1 of cycles coinciding with a radiographic assessment through the 5th cycle. PMBCs will be collected C1D1, C1D3 or C1D7 (depending on schedule) and C2D1. (see section 9 of protocol)
	i. Optional
	j. After 4-6 cycles of the combination therapy, those patients who transition to carboplatin and Talazoparib (BMN 673) combination (without paclitaxel) only need CBC/diff and serum chemistries on D1 of each cycle. Follow Study Calendar B..
	k. If labs are drawn within the time window allotted, but do not meet criteria for treatment, they can be repeated and can be used for treatment decisions.
	l. If screening physical exam done within 7 days of cycle 1 day 1, this does not need to be repeated

B. Study calendar for patients being continued on carboplatin and Talazoparib (BMN 673) without paclitaxel or cycle 1 and 2 for single agent Talazoparib (BMN 673) (after 4-6 cycles of the combination therapy as described in Section 5.1)

	CND1 +/- 3d	CND8 +/- 1d	CND15 +/- 1d	Disease Progression[d] + 3 weeks	Off Study[f]
Talazoparib(BMN 673)	A				
Carboplatin	B				
Concurrent meds	X --X				
Physical exam	X				
Vital signs	X				
Weight	X				
Performance status	X				
CBC w/diff, plts[g, b]	X	X	X		
Serum chemistry[a, b, g]	X	X	X		
Adverse event evaluation	X --X				
Tumor Measurements	Tumor measurements are repeated every 6 weeks (+/- 1 week). Documentation (radiologic) must be provided for patients removed from study for progressive disease[c]				
Radiologic evaluation	Radiologic measurements should be performed every 6 weeks (+/- 1 week).[c]				
Other correlative studies[e] (see section 9 for detail about PK and PBMC collection time points)					
Tumor Biopsy[d]				X	

A: *Talazoparib BMN 673: administration schedule continued oral once daily for either 3 or 7 days based on assigned cohort if carboplatin being co- administered. If changed to single agent Talazoparib (BMN 673) this should be dosed once daily on days 1-21 (continuous dosing). Patients with no prior dose modification of Talazoparib (BMN 673) should have the dose changed to 1000mcg per day (the single agent MTD). Patients who met criteria for a dose modification of Talazoparib (BMN 673) should be continued at the Talazoparib (BMN 673) dose required after the modification.*

B: *Carboplatin: ; administration schedule once iv on day 1 of each cycle or omitted if changing to single agent Talazoparib (BMN 673).*

a: Albumin, alkaline phosphatase, total bilirubin, bicarbonate, BUN, calcium, chloride, creatinine, glucose, LDH, phosphorus, potassium, total protein, SGOT [AST], SGPT [ALT], sodium.

b: After 4-6 cycles of the combination therapy, those patients who transition to carboplatin and Talazoparib (BMN 673) combination (without paclitaxel) only need CBC/diff and serum chemistries on D1 of each cycle. For those who transition to Talazoparib (BMN 673) daily alone, during the first cycle with this new schedule, CBC/diff and serum chemistries are required on D1, D8 and D15. For second cycle--, a CBC/diff and serum chemistries are only required on day 1 of cycle. For patients who transition to observation, physical exam, vitals, CBC/diff and CMP follow up will be per discretion of treating physician, but no less frequent than every 2 months.

c: Preferred radiologic measurements include CXR, CT chest, CT or MRI abdomen, CT or MRI pelvis, bone scan. PET/CT may be used in appropriate cases with PI approval. After 4 cycles of study therapy,tumor and radiologic measurements can be spaced to every 6-12 weeks. For patients on study therapy for over 18 months, radiologic tumor measurements can be repeated less frequently, per MD discretion, but no longer than every 6 months.

d: At time of disease progression, repeat tumor biopsy for all patients with RECIST defined clinical response or those with evaluable disease with clinician defined response (see section 9.0)

e: .Pharmacokinetic samples will be collected C1D1, C1D3 or C1D7 (depending on schedule), C2D1 and for cycle 3 and beyond only on day 1 of cycles coinciding with a radiographic assessment through the 5th cycle. PMBCs will be collected C1D1, C1D3 or C1D7 (depending on schedule) and C2D1. (see section 9 of protocol)

f. Off-study evaluations

g. If labs are drawn within the time window allotted, but do not meet criteria for treatment, they can be repeated and can be used for treatment decisions.

C. Study calendar for patients being continued on single agent Talazoparib (BMN 673) and have completed 2 cycles of Talazoparib (BMN 673) alone. (Note, 1 cycle now changes to 42 days)

	CND1 +/- 3d	CND21 +/- 3d		Disease Progression[d] + 3 weeks	Off Study[f]
Talazoparib (BMN 673)	A				
Concurrent meds	X--X				
Physical exam	X				
Vital signs	X				
Weight	X				
Performance status	X				
CBC w/diff, plts[g]	X	X			
Serum chemistry[a, g]	X	X			
Adverse event evaluation	X--X				
Tumor Measurements	Tumor measurements are repeated every 6- 12 weeks (+/- 1 week). Documentation (radiologic) must be provided for patients removed from study for progressive disease [c]				
Radiologic evaluation	Radiologic measurements should be performed every _6 -12 weeks (+/- 1 week).[c]				
Other correlative studies[e] (see section 9 for detail about PK and PBMC collection time points)					
Tumor Biopsy[d]				X	

A: *Talazoparib (BMN 673): After change to single agent Talazoparib (BMN 673) this should be dosed once daily on days 1-42 (continuous dosing).*

a: Albumin, alkaline phosphatase, total bilirubin, bicarbonate, BUN, calcium, chloride, creatinine, glucose, LDH, phosphorus, potassium, total protein, SGOT [AST], SGPT [ALT], sodium.

c: Preferred radiologic measurements include CXR, CT chest, CT or MRI abdomen, CT or MRI pelvis, bone scan. PET/CT may be used in appropriate cases with PI approval. After 4 cycles of study therapy,tumor and radiologic measurements can be spaced to every 6-12 weeks.
For patients on study therapy for over 18 months, radiologic tumor measurements can be repeated less frequently, per MD discretion, but no longer than every 6 months.

d: At time of disease progression, repeat tumor biopsy for all patients with RECIST defined clinical response or those with evaluable disease with clinician defined response (see section 9.0)

e: Pharmacokinetic samples will be collected C1D1, C1D3 or C1D7 (depending on schedule), C2D1 and for cycle 3 and beyond only on day 1 of cycles coinciding with a radiographic assessment through the 5th cycle. PMBCs will be collected C1D1, C1D3 or C1D7 (depending on schedule) and C2D1. (see section 9 of protocol)

f. Off-study evaluations

g. If labs are drawn within the time window allotted, but do not meet criteria for treatment, they can be repeated and can be used for treatment decisions.

11 MEASUREMENT OF EFFECT

Although the clinical benefit of [this/these] drug(s) has not yet been established, the intent of offering this treatment is to provide a possible therapeutic benefit, and thus the patient will be carefully monitored for tumor response and symptom relief in addition to safety and tolerability. Patients with measurable disease will be assessed by standard criteria. For the purposes of this study, patients should be re-evaluated every 6 weeks for first 4 cycles, then may be extended to 9-12 weeks. In addition to a baseline scan, confirmatory scans will also be obtained 4-8 weeks following initial documentation of an objective response.

11.1 Antitumor Effect – Solid Tumors

For the purposes of this study, patients should be re-evaluated for response every 6 weeks for first 4 cycles, then may be extended to 9-12 weeks. In addition to a baseline scan, confirmatory scans should also be obtained 4-8 (not less than 4) weeks following initial documentation of objective response.

Response and progression will be evaluated in this study using the new international criteria proposed by the revised Response Evaluation Criteria in Solid Tumors (RECIST) guideline (version 1.1).[79] Changes in the largest diameter (unidimensional measurement) of the tumor lesions and the shortest diameter in the case of malignant lymph nodes are used in the RECIST criteria.

11.1.1 Definitions

Evaluable for DLT: The DLT period includes cycle 1 in this trial. If an enrolled subject is discontinued from study treatment for reasons other than safety (e.g., withdrawal of consent, non-compliance, disease progression) prior to completing less than 1 complete cycles OR experiences a study treatment delay within Cycle 1 of more than 7 days for reasons other than safety, he or she will be replaced (i.e., an additional subject will be added to the cohort). Subjects who are replaced will not be considered in making dose- escalation decisions, but if possible, will be followed for safety and other assessments per the protocol.

Evaluable for toxicity. All patients will be evaluable for toxicity from the time of their first treatment with Talazoparib (BMN 673).

Evaluable for objective response. Only those patients who have measurable disease present at baseline, have received at least one cycle of therapy, and have had their disease re-evaluated will be considered evaluable for response. These patients will have their response classified according to the definitions stated below. (Note: Patients who exhibit objective disease progression prior to the end of cycle 1 will also be considered evaluable.)

Evaluable Non-Target Disease Response. Patients who have lesions present at baseline that are evaluable but do not meet the definitions of measurable disease, have received at least one cycle of therapy, and have had their disease re-evaluated will be considered

evaluable for non-target disease. The response assessment is based on the presence, absence, or unequivocal progression of the lesions.

11.1.2 Disease Parameters

Measurable disease. Measurable lesions are defined as those that can be accurately measured in at least one dimension (longest diameter to be recorded) as ≥20 mm (≥2 cm) by chest x-ray or as ≥10 mm (≥1 cm) with CT scan, MRI, or calipers by clinical exam. All tumor measurements must be recorded in millimeters (or decimal fractions of centimeters).

Note: Tumor lesions that are situated in a previously irradiated area might or might not be considered measurable. *If the investigator thinks it appropriate to include them, the conditions under which such lesions should be considered must be defined in the protocol.*

Malignant lymph nodes. To be considered pathologically enlarged and measurable, a lymph node must be ≥15 mm (≥1.5 cm) in short axis when assessed by CT scan (CT scan slice thickness recommended to be no greater than 5 mm [0.5 cm]). At baseline and in follow-up, only the short axis will be measured and followed.

Non-measurable disease. All other lesions (or sites of disease), including small lesions (longest diameter <10 mm [<1 cm] or pathological lymph nodes with ≥10 to <15 mm [≥1 to <1.5 cm] short axis), are considered non-measurable disease. Bone lesions, leptomeningeal disease, ascites, pleural/pericardial effusions, lymphangitis cutis/pulmonitis, inflammatory breast disease, and abdominal masses (not followed by CT or MRI), are considered as non-measurable.

Note: Cystic lesions that meet the criteria for radiographically defined simple cysts should not be considered as malignant lesions (neither measurable nor non-measurable) since they are, by definition, simple cysts.

'Cystic lesions' thought to represent cystic metastases can be considered as measurable lesions, if they meet the definition of measurability described above. However, if non-cystic lesions are present in the same patient, these are preferred for selection as target lesions.

Target lesions. All measurable lesions up to a maximum of 2 lesions per organ and 5 lesions in total, representative of all involved organs, should be identified as **target lesions** and recorded and measured at baseline. Target lesions should be selected on the basis of their size (lesions with the longest diameter), be representative of all involved organs, but in addition should be those that lend themselves to reproducible repeated measurements. It may be the case that, on occasion, the largest lesion does not lend itself to reproducible measurement in which circumstance the next largest lesion which can be measured reproducibly should be selected. A sum of the diameters (longest for non-nodal lesions, short axis for nodal lesions) for all target lesions will be calculated and reported as the baseline sum diameters. If lymph nodes are to be included in the sum,

then only the short axis is added into the sum. The baseline sum diameters will be used as reference to further characterize any objective tumor regression in the measurable dimension of the disease.

Non-target lesions. All other lesions (or sites of disease) including any measurable lesions over and above the 5 target lesions should be identified as **non-target lesions** and should also be recorded at baseline. Measurements of these lesions are not required, but the presence, absence, or in rare cases unequivocal progression of each should be noted throughout follow-up.

11.1.3 Methods for Evaluation of Measurable Disease

All measurements should be taken and recorded in metric notation using a ruler or calipers. All baseline evaluations should be performed as closely as possible to the beginning of treatment and never more than 4 weeks before the beginning of the treatment.

The same method of assessment and the same technique should be used to characterize each identified and reported lesion at baseline and during follow-up. Imaging-based evaluation is preferred to evaluation by clinical examination unless the lesion(s) being followed cannot be imaged but are assessable by clinical exam.

Clinical lesions Clinical lesions will only be considered measurable when they are superficial (*e.g.,* skin nodules and palpable lymph nodes) and ≥10 mm (≥1 cm) diameter as assessed using calipers (*e.g.*, skin nodules). In the case of skin lesions, documentation by color photography, including a ruler to estimate the size of the lesion, is recommended.

Chest x-ray Lesions on chest x-ray are acceptable as measurable lesions when they are clearly defined and surrounded by aerated lung. However, CT is preferable.

Conventional CT and MRI This guideline has defined measurability of lesions on CT scan based on the assumption that CT slice thickness is 5 mm (0.5 cm) or less. If CT scans have slice thickness greater than 5 mm (0.5 cm), the minimum size for a measurable lesion should be twice the slice thickness. MRI is also acceptable in certain situations (*e.g.* for body scans).

Use of MRI remains a complex issue. MRI has excellent contrast, spatial, and temporal resolution; however, there are many image acquisition variables involved in MRI, which greatly impact image quality, lesion conspicuity, and measurement. Furthermore, the availability of MRI is variable globally. As with CT, if an MRI is performed, the technical specifications of the scanning sequences used should be optimized for the evaluation of the type and site of disease. Furthermore, as with CT, the modality used at follow-up should be the same as was used at baseline and the lesions should be measured/assessed on the same pulse sequence. It is beyond the scope of the RECIST guidelines to prescribe specific MRI pulse sequence parameters for all scanners, body

parts, and diseases. Ideally, the same type of scanner should be used and the image acquisition protocol should be followed as closely as possible to prior scans. Body scans should be performed with breath-hold scanning techniques, if possible.

PET-CT At present, the low dose or attenuation correction CT portion of a combined PET-CT is not always of optimal diagnostic CT quality for use with RECIST measurements. However, if the site can document that the CT performed as part of a PET-CT is of identical diagnostic quality to a diagnostic CT (with IV and oral contrast), then the CT portion of the PET-CT can be used for RECIST measurements and can be used interchangeably with conventional CT in accurately measuring cancer lesions over time. Note, however, that the PET portion of the CT introduces additional data which may bias an investigator if it is not routinely or serially performed.

Ultrasound Ultrasound is not useful in assessment of lesion size and should not be used as a method of measurement. Ultrasound examinations cannot be reproduced in their entirety for independent review at a later date and, because they are operator dependent, it cannot be guaranteed that the same technique and measurements will be taken from one assessment to the next. If new lesions are identified by ultrasound in the course of the study, confirmation by CT or MRI is advised. If there is concern about radiation exposure at CT, MRI may be used instead of CT in selected instances.

Endoscopy, Laparoscopy The utilization of these techniques for objective tumor evaluation is not advised. However, such techniques may be useful to confirm complete pathological response when biopsies are obtained or to determine relapse in trials where recurrence following complete response (CR) or surgical resection is an endpoint.

Tumor markers Tumor markers alone cannot be used to assess response. If markers are initially above the upper normal limit, they must normalize for a patient to be considered in complete clinical response. Specific guidelines for both CA-125 response (in recurrent ovarian cancer) and PSA response (in recurrent prostate cancer) have been published.[80-82] In addition, the Gynecologic Cancer Intergroup has developed CA-125 progression criteria which are to be integrated with objective tumor assessment for use in first-line trials in ovarian cancer.[83]

Cytology, Histology These techniques can be used to differentiate between partial responses (PR) and complete responses (CR) in rare cases (*e.g.*, residual lesions in tumor types, such as germ cell tumors, where known residual benign tumors can remain).

The cytological confirmation of the neoplastic origin of any effusion that appears or worsens during treatment when the measurable tumor has met criteria for response or stable disease is mandatory to differentiate between response or stable disease (an effusion may be a side effect of the treatment) and progressive disease.

FDG-PET While FDG-PET response assessments need additional study, it is sometimes reasonable to incorporate the use of FDG-PET scanning to complement CT scanning in

assessment of progression (particularly possible 'new' disease). New lesions on the basis of FDG-PET imaging can be identified according to the following algorithm:

a. Negative FDG-PET at baseline, with a positive FDG-PET at follow-up is a sign of PD based on a new lesion.
b. No FDG-PET at baseline and a positive FDG-PET at follow-up: If the positive FDG- PET at follow-up corresponds to a new site of disease confirmed by CT, this is PD. If the positive FDG-PET at follow-up is not confirmed as a new site of disease on CT, additional follow-up CT scans are needed to determine if there is truly progression occurring at that site (if so, the date of PD will be the date of the initial abnormal FDG-PET scan). If the positive FDG-PET at follow-up corresponds to a pre-existing site of disease on CT that is not progressing on the basis of the anatomic images, this is not PD.
c. FDG-PET may be used to upgrade a response to a CR in a manner similar to a biopsy in cases where a residual radiographic abnormality is thought to represent fibrosis or scarring. The use of FDG-PET in this circumstance should be prospectively described in the protocol and supported by disease-specific medical literature for the indication. However, it must be acknowledged that both approaches may lead to false positive CR due to limitations of FDG-PET and biopsy resolution/sensitivity.

Note: A ‘positive’ FDG-PET scan lesion means one which is FDG avid with an uptake greater than twice that of the surrounding tissue on the attenuation corrected image.

11.1.4 Response Criteria

11.1.4.1 Evaluation of Target Lesions

Complete Response (CR): Disappearance of all target lesions. Any pathological lymph nodes (whether target or non-target) must have reduction in short axis to <10 mm (<1 cm).

Partial Response (PR): At least a 30% decrease in the sum of the diameters of target lesions, taking as reference the baseline sum diameters.

Progressive Disease (PD): At least a 20% increase in the sum of the diameters of target lesions, taking as reference the smallest sum on study (this includes the baseline sum if that is the smallest on study). In addition to the relative increase of 20%, the sum must also demonstrate an absolute increase of at least 5 mm (0.5 cm). (Note: the appearance of one or more new lesions is also considered progressions).

Stable Disease (SD): Neither sufficient shrinkage to qualify for PR nor sufficient increase to qualify for PD, taking as reference the smallest sum diameters while on study.

11.1.4.2 Evaluation of Non-Target Lesions

Complete Response (CR): Disappearance of all non-target lesions and normalization of tumor

marker level. All lymph nodes must be non-pathological in size (<10 mm [<1 cm] short axis).

Note: If tumor markers are initially above the upper normal limit, they must normalize for a patient to be considered in complete clinical response.

Non-CR/Non-PD: Persistence of one or more non-target lesion(s) and/or maintenance of tumor marker level above the normal limits.

Progressive Disease (PD): Appearance of one or more new lesions and/or *unequivocal progression* of existing non-target lesions. *Unequivocal progression* should not normally trump target lesion status. It must be representative of overall disease status change, not a single lesion increase.

Although a clear progression of "non-target" lesions only is exceptional, the opinion of the treating physician should prevail in such circumstances, and the progression status should be confirmed at a later time by the review panel (or Principal Investigator).

11.1.4.3 Evaluation of Best Overall Response

The best overall response is the best response recorded from the start of the treatment until disease progression/recurrence (taking as reference for progressive disease the smallest measurements recorded since the treatment started). The patient's best response assignment will depend on the achievement of both measurement and confirmation criteria.

For Patients with Measurable Disease (*i.e.*, Target Disease)

Target Lesions	Non-Target Lesions	New Lesions	Overall Response	Best Overall Response when Confirmation is Required*
CR	CR	No	CR	≥4 wks. Confirmation**
CR	Non-CR/Non-PD	No	PR	≥4 wks. Confirmation**
CR	Not evaluated	No	PR	
PR	Non-CR/Non-PD/not evaluated	No	PR	
SD	Non-CR/Non-PD/not evaluated	No	SD	Documented at least once ≥4 wks. from baseline**
PD	Any	Yes or No	PD	No prior SD, PR or CR
Any	PD***	Yes or No	PD	
Any	Any	Yes	PD	

*	See RECIST 1.1 manuscript for further details on what is evidence of a new lesion.
**	Only for non-randomized trials with response as primary endpoint.
***	In exceptional circumstances, unequivocal progression in non-target lesions may be accepted as disease progression.
Note:	Patients with a global deterioration of health status requiring discontinuation of treatment without objective evidence of disease progression at that time should be reported as "*symptomatic deterioration.*" Every effort should be made to document the objective progression even after discontinuation of treatment.

For Patients with Non-Measurable Disease (*i.e.*, Non-Target Disease)

Non-Target Lesions	New Lesions	Overall Response
CR	No	CR
Non-CR/non-PD	No	Non-CR/non-PD*
Not all evaluated	No	not evaluated
Unequivocal PD	Yes or No	PD
Any	Yes	PD
* 'Non-CR/non-PD' is preferred over 'stable disease' for non-target disease since SD is increasingly used as an endpoint for assessment of efficacy in some trials so to assign this category when no lesions can be measured is not advised		

11.1.5 Duration of Response

Duration of overall response: The duration of overall response is measured from the time measurement criteria are met for CR or PR (whichever is first recorded) until the first date that recurrent or progressive disease is objectively documented (taking as reference for progressive disease the smallest measurements recorded since the treatment started).

The duration of overall CR is measured from the time measurement criteria are first met for CR until the first date that progressive disease is objectively documented.

Duration of stable disease: Stable disease is measured from the start of the treatment until the criteria for progression are met, taking as reference the smallest measurements recorded since the treatment started, including the baseline measurements.

11.1.6 Progression-Free Survival-N/A

11.1.7 Response Review-N/A

11.2 Antitumor Effect – Hematologic Tumors- N/A

11.3 Other Response Parameters-N/A

12 STUDY OVERSIGHT AND DATA REPORTING / REGULATORY REQUIREMENTS

Study Oversight

This protocol is monitored at several levels, as described in this section. The Protocol Principal Investigator is responsible for monitoring the conduct and progress of the clinical trial, including the ongoing review of accrual, patient-specific clinical and laboratory data, and routine and serious adverse events; reporting of expedited adverse events; and accumulation of reported adverse events from other trials testing the same drug(s). The Protocol Principal Investigator and statistician have access to the data at all times through the CTMS web-based reporting portal.

For the Phase 1 portion of this study, all decisions regarding dose escalation/expansion/de-escalation require sign-off by the Protocol Principal Investigator through the CTMS/IWRS. In addition, for the Phase 1 portion, the Protocol Principal Investigator will have at least monthly, or more frequently, conference calls with the Study Investigators and the CTEP Medical Officer(s) to review accrual, progress, and adverse events and unanticipated problems.

All Study Investigators at participating sites who register/enroll patients on a given protocol are responsible for timely submission of data via Medidata Rave and timely reporting of adverse events for that particular study. This includes timely review of data collected on the electronic CRFs submitted via Medidata Rave.

All studies are also reviewed in accordance with the enrolling institution's data safety monitoring plan.

Adverse event lists, guidelines, and instructions for AE reporting can be found in Section 7.0 (Adverse Events: List and Reporting Requirements).

12.1 Data Reporting

Data collection for this study will be done exclusively through Medidata Rave. Access to the trial in Rave is granted through the iMedidata application to all persons with the appropriate roles assigned in the Regulatory Support System (RSS). To access Rave via iMedidata, the site user must have an active CTEP IAM account (check at < https://ctepcore.nci.nih.gov/iam >) and the appropriate Rave role (Rave CRA, Read-Only, CRA, Lab Admin, SLA, or Site Investigator) on either the LPO or participating organization roster at the enrolling site. To the hold Rave CRA role or CRA Lab Admin role, the user must hold a minimum of an AP registration type. To hold the Rave Site Investigator role, the individual must be registered as an NPIVR or IVR. Associates can hold read-only roles in Rave.

Upon initial site registration approval for the study in RSS, all persons with Rave roles assigned on the appropriate roster will be sent a study invitation e-mail from iMedidata. To accept the invitation, site users must log into the Select Login (https://login.imedidata.com/selectlogin)

using their CTEP-IAM user name and password, and click on the "accept" link in the upper right-corner of the iMedidata page. Please note, site users will not be able to access the study in Rave until all required Medidata and study specific trainings are completed. Trainings will be in the form of electronic learnings (eLearnings), and can be accessed by clicking on the link in the upper right pane of the iMedidata screen.

Users that have not previously activated their iMedidata/Rave account at the time of initial site registration approval for the study in RSS will also receive a separate invitation from iMedidata to activate their account. Account activation instructions are located on the CTSU website, Rave tab under the Rave resource materials (Medidata Account Activation and Study Invitation Acceptance). Additional information on iMedidata/Rave is available on the CTSU members' website under the Rave tab or by contacting the CTSU Help Desk at 1-888-823-5923 or by e-mail at ctsucontact@westat.com.

12.1.1 Method

This study will be monitored by the Clinical Trials Monitoring Service (CTMS). Data will be submitted to CTMS at least once every two weeks via Medidata Rave (or other modality if approved by CTEP). Information on CTMS reporting is available at http://www.theradex.com/clinicalTechnologies/?National-Cancer-Institute-NCI-11. Data will be submitted to CTMS at least once every two weeks on the NCI/DCTD case report form or the electronic case report form (ACES). On-site audits will be conducted three times annually (one annual site visit and two data audits). For CTMS monitored studies, after users have activated their accounts, please contact the Theradex Help Desk at (609) 799-7580 or by email at CTMSSupport@theradex.com for additional support with Rave and completion of CRFs.

12.1.2 Responsibility for Data Submission

For ETCTN trials, it is the responsibility of the PI(s) at the site to ensure that all investigators at the ETCTN Sites understand the procedures for data submission for each ETCTN protocol and that protocol specified data are submitted accurately and in a timely manner to the CTMS via the electronic data capture system, Medidata Rave.

Data are to be submitted via Medidata Rave to CTMS on a real-time basis, but no less than once every 2 weeks. The timeliness of data submissions and timeliness in resolving data queries will be tracked by CTMS. Metrics for timeliness will be followed and assessed on a quarterly basis. For the purpose of Institutional Performance Monitoring, data will be considered delinquent if it is greater than 4 weeks past due.

Data from Medidata Rave and CTEP-AERS is reviewed by the CTMS on an ongoing basis as data is received. Queries will be issued by CTMS directly within Rave. The queries will appear on the Task Summary Tab within Rave for the CRA at the ETCTN to resolve. Monthly web- based reports are posted for review by the Drug Monitors in the IDB, CTEP. Onsite audits will be conducted by the CTMS to ensure compliance with regulatory requirements, GCP, and NCI policies and procedures with the overarching goal of ensuring the integrity of data generated from NCI-sponsored clinical trials, as described in the ETCTN Program Guidelines, which may

be found on the CTEP (http://ctep.cancer.gov/protocolDevelopment/electronic_applications/adverse_events.htm) and CTSU websites.

An End of Study CRF is to be completed by the PI, and is to include a summary of study endpoints not otherwise captured in the database, such as (for phase 1 trials) the recommended phase 2 dose (RP2D), and a description of any dose-limiting toxicities (DLTs). CTMS will utilize a core set of eCRFs that are Cancer Data Standards Registry and Repository (caDSR) compliant (http://cbiit.nci.nih.gov/ncip/biomedical-informatics-resources/interoperability-and-semantics/metadata-and-models). Customized eCRFs will be included when appropriate to meet unique study requirements. The PI is encouraged to review the eCRFs, working closely with CTMS to ensure prospectively that all required items are appropriately captured in the eCRFs prior to study activation. CTMS will prepare the eCRFs with built-in edit checks to the extent possible to promote data integrity.

CDUS data submissions for ETCTN trials activated after March 1, 2014, will be carried out by the CTMS contractor, Theradex. CDUS submissions are performed by Theradex on a monthly basis. The trial's lead institution is responsible for timely submission to CTMS via Rave, as above.

Further information on data submission procedures can be found in the ETCTN Program Guidelines (http://ctep.cancer.gov/protocolDevelopment/electronic_applications/adverse_events.htm).

See Section 12.1.1 for details on CDUS reporting. As the data management center for this trial, Theradex is responsible for compiling and submitting CDUS data to CTEP for all participants and for providing the data to the Principal Investigator for review.

12.2 CTEP Multicenter Guidelines: N/A

12.3 Collaborative Agreements Language

The agent(s) supplied by CTEP, DCTD, NCI used in this protocol is/are provided to the NCI under a Collaborative Agreement (CRADA, CTA, CSA) between the Pharmaceutical Company(ies) (hereinafter referred to as "Collaborator(s)") and the NCI Division of Cancer Treatment and Diagnosis. Therefore, the following obligations/guidelines, in addition to the provisions in the "Intellectual Property Option to Collaborator"

(http://ctep.cancer.gov/industryCollaborations2/intellectual_property.htm) contained within the terms of award, apply to the use of the Agent(s) in this study:

1. Agent(s) may not be used for any purpose outside the scope of this protocol, nor can Agent(s) be transferred or licensed to any party not participating in the clinical study. Collaborator(s) data for Agent(s) are confidential and proprietary to Collaborator(s) and shall be maintained as such by the investigators. The protocol documents for studies utilizing Agents contain confidential information and should not be shared or distributed without the permission of the NCI. If a copy of this protocol is requested by a patient or patient's family member participating on the study, the individual should sign a confidentiality agreement. A suitable model agreement can be downloaded from: http://ctep.cancer.gov.

2. For a clinical protocol where there is an investigational Agent used in combination with (an)other Agent(s), each the subject of different Collaborative Agreements, the access to and use of data by each Collaborator shall be as follows (data pertaining to such combination use shall hereinafter be referred to as "Multi-Party Data"):

    a. NCI will provide all Collaborators with prior written notice regarding the existence and nature of any agreements governing their collaboration with NCI, the design of the proposed combination protocol, and the existence of any obligations that would tend to restrict NCI's participation in the proposed combination protocol.

    b. Each Collaborator shall agree to permit use of the Multi-Party Data from the clinical trial by any other Collaborator solely to the extent necessary to allow said other Collaborator to develop, obtain regulatory approval or commercialize its own Agent.

    c. Any Collaborator having the right to use the Multi-Party Data from these trials must agree in writing prior to the commencement of the trials that it will use the Multi-Party Data solely for development, regulatory approval, and commercialization of its own Agent.

3. Clinical Trial Data and Results and Raw Data developed under a Collaborative Agreement will be made available to Collaborator(s), the NCI, and the FDA, as appropriate and unless additional disclosure is required by law or court order as described in the IP Option to Collaborator (http://ctep.cancer.gov/industryCollaborations2/intellectual_property.htm). Additionally, all Clinical Data and Results and Raw Data will be collected, used and disclosed consistent with all applicable federal statutes and regulations for the protection of human subjects, including, if applicable, the *Standards for Privacy of Individually Identifiable Health Information* set forth in 45 C.F.R. Part 164.

4. When a Collaborator wishes to initiate a data request, the request should first be sent to the NCI, who will then notify the appropriate investigators (Group Chair for Cooperative Group studies, or PI for other studies) of Collaborator's wish to contact them.

5. Any data provided to Collaborator(s) for Phase 3 studies must be in accordance with the guidelines and policies of the responsible Data Monitoring Committee (DMC), if there is a

DMC for this clinical trial.

6. Any manuscripts reporting the results of this clinical trial must be provided to CTEP by the Group office for Cooperative Group studies or by the principal investigator for non-Cooperative Group studies for immediate delivery to Collaborator(s) for advisory review and comment prior to submission for publication. Collaborator(s) will have 30 days from the date of receipt for review. Collaborator shall have the right to request that publication be delayed for up to an additional 30 days in order to ensure that Collaborator's confidential and proprietary data, in addition to Collaborator(s)'s intellectual property rights, are protected. Copies of abstracts must be provided to CTEP for forwarding to Collaborator(s) for courtesy review as soon as possible and preferably at least three (3) days prior to submission, but in any case, prior to presentation at the meeting or publication in the proceedings. Press releases and other media presentations must also be forwarded to CTEP prior to release. Copies of any manuscript, abstract and/or press release/ media presentation should be sent to:

Email: ncicteppubs@mail.nih.gov

The Regulatory Affairs Branch will then distribute them to Collaborator(s). No publication, manuscript or other form of public disclosure shall contain any of Collaborator's confidential/ proprietary information.

13 STATISTICAL CONSIDERATIONS

13.1 Study Design/Endpoints

Endpoints: The primary endpoints are safety and tolerability of Talazoparib (BMN 673) seven day and three day schedule in combination with carboplatin and paclitaxel. Adverse events will be evaluated by type and severity using the NCI CTCAE version 4.0 until March 31, 2018. CTCAE version 5.0 will be utilized beginning April 1, 2018. Adverse events data collection include adverse event diagnosis, date of onset and resolution, whether the event is ongoing, CTCAE grade, whether the event is serious, frequency, and outcome status, and action taken. The secondary endpoints are anti-tumor response (as determined by RECIST criteria) and pharmacokinetic parameters.

Study Design: The primary objectives of this phase I study are: (1) To determine the MTD and RP2D of Talazoparib (BMN 673) seven day schedule in combination with carboplatin and paclitaxel, and (2) to determine the MTD and RP2D of Talazoparib (BMN 673) three day schedule in combination with carboplatin and paclitaxel.

Hence, two MTDs and RP2Ds (for each schedule) will be determined from this phase I trial. In schedule A, Talazoparib (BMN 673) will be administered once daily for 7 days total. In schedule B, Talazoparib (BMN 673) will be administered once daily for 3 days total. Dose escalation for the two schedules will be conducted sequentially, starting with schedule A. Once the MTD of schedule A has been determined, the MTD dose cohort for schedule A will be expanded to a total of 12 evaluable patients. If no more than 4 of the evaluable 12 total patients treated at the MTD experience a DLT, then the starting dose level for schedule B will be the MTD from Schedule A. If more than 4 patients experience a DLT with the dose expansion cohort at the MTD for schedule A, a conference call will be held with the sponsor to establish the starting dose for schedule B. If dose level -1 in Schedule A exceeds the MTD, then dose level -1 will be used as the starting dose for schedule B. After schedule B reaches the MTD, a dose expansion of 6 patients at the MTD will occur for a total of 12 evaluable patients. Accrual to the dose expansion cohort for schedule B will be terminated early if there is indication an excessive toxicity rate. The toxicity rate will be considered as excessive if during any time of the dose expansion period the lower limit of the one-sided 90% confidence interval for the DLT rate exceeds 20%. Operationally, this will occur if 4 out of 9 patients or 5 out of 12 (or less) patients in the schedule B dose cohort experience DLTs. If no more than 4 of the evaluable 12 total patients treated at the MTD in the expanded dose cohort experience a DLT, then this will be the RP2D for schedule B. If more than 4 patients experience a DLT with the dose expansion cohort at the MTD for schedule B, a conference call will be held with the sponsor to establish the RP2D. The MTD for each schedule will be used as the RP2D. In the unlikely event that the MTD determined from schedule B is lower than the MTD determined from schedule A, then the MTD from schedule B will be used as the RP2D for schedule A.

Dose escalation within each schedule will be conducted using a traditional 3+3 phase I design. Specifically, patients will be accrued in cohorts of three. If no DLT is observed in these 3 patients, a new cohort of 3 patients will be treated at the next higher dose level. If one of the initial 3 patients develops a DLT, then 3 additional patients (total of 6) will be added to that

same dose level. If DLT is observed in only one of 6 patients at a given dose level, the next cohort of patients will be allowed to start at the next higher dose level. If two or more patients experience DLT at a particular dose level, then the dose escalation scheme will cease and any subsequent patients will be treated at the previous (lower) dose level. The MTD within each schedule will be defined as the highest safely tolerated dose where 0/6 or 1/6 (less than 33%) patients experience a DLT and two or more patients have experienced a DLT at the next higher dose level. If, within a schedule, the highest dose level (dose level 6) is reached without observing DLTs in two or more patients, then dose level will be defined as the MTD. The MTD within each schedule will be used as the RP2D.

13.2 Sample Size/Accrual Rate

Sample Size Justification: As this is a Phase I dose escalation study designed to determine the MTD and RP2D of Talazoparib (BMN 673) with seven and three day schedule in combination with carboplatin and paclitaxel, no formal power calculations were performed. Rather, the sample size chosen was based on typical dose escalation designs (with 3-6 subjects per dose level). The total number of subjects treated in the study will depend on the number of dose levels tested and the number of subjects treated in each cohort before the MTD has been determined. Within each of the two treatment schedules (seven day and three day schedule), between 18 to 36 patients are required to complete 6 dose levels. The probabilities of escalating to the next dose level, based on the true DLT rate at the current dose, are given in the following table:

Table 13.2.1 Probabilities of dose escalation

	True Toxicity Rate at a Given Dose						
	10%	20%	30%	40%	50%	60%	70%
Probability of Escalation	0.91	0.71	0.49	0.31	0.17	0.08	0.03

Thus, if the true DLT rate is 30% at the current dose, there is a 49% chance of escalating to the next dose.

While the sample size per group is too small to allow a meaningful direct comparison of toxicity rates between dose levels, comparisons of pharmacokinetic parameters and correlative measures (evaluating mechanism of resistance) suggest whether differences are likely. With 6 patients per dose level, the power to detect average differences of two standard deviation units in pharmacokinetic parameters is 88% at the (two-sided) 5% level of significance. Differences of 1.5 standard deviation units would be detected with 64% power. Smaller differences are unlikely to be detected as statistically significant. These calculations are based on a two-sample t-test under the assumptions that the data are normally distributed and that the between-patient variability is the same across dose levels.

Once the MTD has been reached within a schedule, the dose cohort will be expanded to 6 patients to a total of 12 patients to (1) further characterize the safety and tolerability of the combination, and (2) to provide estimates for preliminary anti-tumor activity with adequate precision levels. The proposed sample size of 12 patients treated at the MTD is sufficient to detect a DLT in at least one patient with a high probability. The following table shows the probabilities for observing a DLT in at least one of the 12 patients treated at the MTD.

Table 13.2.2: Probabilities of observing a DLT in at least one of 12 patients treated at the MTD

	10%	15%	20%	30%	35%	40%
Probability of detecting at DLT in at least one patient (out of 12)	0.72	0.86	0.93	0.97	0.99	>0.99

That is, if the DLT rate is between 10-35%, then the probability that a DLT will be detected in at least one patient ranges from 72-99%.

Furthermore, a sample size of 12 patients is sufficient to provide accuracy in estimating preliminary anti-tumor activity levels at the RP2D. This will provide important information for designing a subsequent phase II study. Specifically, with a sample size of 12 patients, the overall response rate at the MTD of the treatment combination will be estimated with a standard error of 15% and the length of the two-sided 95% confidence interval will be no wider than 50%.

Accrual: The maximum sample size to complete this study is 66 (60+6 to account for inevaluable patients). It is anticipated that 2-3 patients will be accrued per month so that accrual will be completed within 28 months.

13.3 Stratification Factors-N/A

13.4 Analysis of Secondary Endpoints

Toxicities observed will be summarized in terms of types and severities by the most recent version of the NCI Common Toxicity Criteria for each schedule and dose level separately. The number and severity of toxicity incidents will be analyzed descriptively in tabular format. Comparisons between dose level arms will be performed using Fisher's exact test. Ninety percent confidence intervals for DLT rates will be constructed for dose levels with 6 or more patients. Disease responses will be validated by the RECIST criteria. All patients with measurable disease will be classified as having either: Progressive Disease (PD), Stable Disease (SD), a Partial Response (PR) or a Complete Response (CR). Responses will be summarized by simple descriptive summary statistics delineating complete and partial responses as well as stable and progressive disease, stratified by treatment schedule. Ninety percent confidence intervals for the proportions of subjects with a confirmed anti-tumor response will be computed for dose levels with 6 or more patients. Exact logistic regression analysis will be performed to evaluate the dose-response relationship. Chi-square or Fisher's exact test will be used to compare responses between treatment schedules.

The number of responses to Talazoparib (BMN 673) alone after the combination with carboplatin, paclitaxel and Talazoparib (BMN 673) will be summarized in tabular format. Complete and partial response rates will be determined and reported along with the corresponding 90% confidence intervals. Analogously, the number and frequency of toxicities of Talazoparib (BMN 673) alone after the combination with carboplatin, paclitaxel and Talazoparib (BMN 673) will be summarized in tabular format, stratified by type and severity.

The PK parameters listed above will be calculated via non-compartmental analysis. All PK parameters will be summarized by treatment schedule dose level using standard descriptive statistics: means, medians, ranges, and standard deviations (if numbers and distribution permit). The Jonckheere-Terpstra trend test will be performed to determine the significance of the association between increasing dose level and each of the pharmacokinetic parameters within each treatment schedule. A Spearman rank correlation analysis will be performed to determine the relationship between actual dose administered and the pharmacokinetic parameters.

Additionally, logistic regression analyses will be performed to correlate PK parameters with toxicity and response. AUC and C will be tested for dose-proportionality within each treatment schedule using the power model. Analysis of covariance (where dose will be included as covariate) will be used to compare PK parameters between treatment schedules.

Analysis of Correlative Outcomes: Cmax levels form plasma samples collected prior to dosing and after carboplatin and paclitaxel administration on day 1 of cycle 1 and 2 and 4 hours post Talazoparib (BMN 673) on cycle 1 day 3 and 7 will be summarized in terms of means, standard deviations and ranges. Linear mixed effects modeling with subject specific random effects will be performed to evaluate Cmax and plasma concentration collected on day 1 and day 3 predict changes in PRMCs. Furthermore, the empirical Bayesian approach will be utilize to incorporate PK data from other phase I studies of the agent to construct a PK population model. [76]

Changes PBMC levels from the cycle 1, day 1 (prior to first Talazoparib (BMN 673) dose) assessment to the cycle 1, day 3 or 7 and cycle 2, day 1 assessments will be evaluated using a two-sample t-test.

The number and frequency of mutation status changes from the baseline biopsy assessment to the biopsy obtained at the time of progression will be summarized in tabular format. The frequency of mutation status changes will be analyzed using a paired t-test or nonparametric Wilcoxon Signed Rank test. A negative binomial regression model will be utilized to account for the dose effect. The presence of individual mutations (from the Foundation One® Panel) will be compared between the baseline and time of progression assessment using a paired McNemar's test. The Benjamini-Hochberg method will be utilized will be utilized to control the false discovery rate. These analyses will be considered as exploratory.

A statistical analysis plan will be developed prior to database lock and will include detailed descriptions of summaries and mock-ups of tables, listings, and figures to be included in the clinical study report.

14 STUDY STATUS UPDATES AND STUDY CLOSURE

14.1 Definitions of Study Status Changes

14.1.1 Temporarily Closed to Accrual

The study status is Temporarily Closed to Accrual when no patient slots are currently available, but there is the possibility that the trial will re-open for accrual (patient slots become available). Sites are not permitted to accrue additional patients until CTEP is notified of Re-Activation.

Study status will need to be changed to Temporarily Closed to Accrual when any of the following criteria are met:

- Sites are notified by CTEP (via Request for Rapid Amendment [RRA]) of changes in the risk/benefit ratio that necessitate changes to the patient Informed Consent document. Requested changes will be specified in the RRA and must be reviewed by the study's IRB.
- CTEP and the lead investigator agree that unacceptable toxicities necessitate a discussion to change the dosing/regimen.
- A protocol-defined benchmark has been achieved (such as an interim analysis before proceeding to the next stage).
- Investigators encounter any of the stopping criteria described in Section 5.4.

14.1.2 Closed to Accrual

The study status is (permanently) Closed to Accrual when no more patient enrollment slots are available, and at least one patient is still actively receiving the study treatment. Sites are no longer permitted to enroll additional patients.

Patient slots are no longer available when the following criteria are met:

- The pre-specified number of evaluable patients has been successfully enrolled, treated, and evaluated.
- The study treatment has failed to meet the pre-specified efficacy goal at the stage 1 interim analysis.
- CTEP and the investigators agree that unacceptable toxicities preclude further enrollment.
- Investigators encounter any of the stopping criteria described in Section 5.4.

14.1.3 Closed to Accrual and Treatment

The study status is Closed to Accrual and Treatment when no more patient enrollment slots are available and no patients are currently receiving the study treatment. Patients may still be enrolled on the protocol only for the purposes of follow-up.

Patient accrual and treatment will be permanently halted when any of the following criteria are met:

- Enrollment was previously closed (study status of "Closed to Accrual"), and no patients are receiving the study treatment.
- CTEP and the investigators agree that unacceptable toxicities preclude further enrollment. In this case, CTEP and the investigators must collaborate to alter the regimen or to halt the study treatment altogether as soon as it can be safely done for patients currently receiving treatment.

CTEP and Theradex **must be notified** when patients are no longer receiving treatment [*i.e.*, when the last patient(s) to be receiving treatment is/are no longer receiving the study regimen for any reason].

14.1.4 Closed to Follow-Up

The study is considered Closed to Follow-Up when all protocol-defined follow-up procedures have been completed for all patients who have not been removed from the study for other reasons. That is, there are no outstanding follow-up procedures to be performed as mandated by the protocol.

CTEP does **not** need to be notified of a status change to "Closed to Follow Up."

14.1.5 Complete

Study is considered Complete if it has been at least thirty (30) days since the last patient follow-up evaluation.

A citation to a final study report (manuscript, meeting abstract, etc.) is required with the submission of the Protocol Status Update Form to CTEP PIO.

14.2 Responsibility for Filing Protocol Status Update Forms

CTEP must be notified of all study status changes in Section 14.1 (except for Closed to Follow-Up) by the Corresponding Organization via Protocol Status Update Form, available from the CTEP website at http://ctep.cancer.gov/protocolDevelopment/default.htm#amendments.

Theradex must be notified as soon as all patients are off treatment (*i.e.*, when study status changes to Closed to Accrual and Treatment). Theradex will produce a report within 90 days of this notification.

REFERENCES

1. Appleman, LA, JH Beumer, Y Jiang, et al., A phase I study of veliparib (ABT-888) in combination with carboplatin and paclitaxel in advanced solid malignancies. J Clin Oncol 2012. suppl; abstr 3049.
2. Rivkin, S, D Iriarte, H Sloan, et al., Phase Ib/II with expansion of patients at the MTD study of olaparib plus weekly (metronomic) carboplatin and paclitaxel in relapsed ovarian cancer. J Clin Oncol 2014. 32:5s (suppl; abstr 5527).
3. Bendell, J, EM O'Reilly, MR Middleton, et al., Phase I study of olaparib plus gemcitabine in patients with advanced solid tumours and comparison with gemcitabine alone in patients with locally advanced/metastatic pancreatic cancer. Ann Oncol., 2015. Jan 7. pii: mdu581. [Epub ahead of print].
4. Siegel, R, E Ward, O Brawley, et al., Cancer statistics, 2011: the impact of eliminating socioeconomic and racial disparities on premature cancer deaths. CA Cancer J Clin, 2011. 61(4): p. 212-36.
5. Chambon, P, JD Weill, and P Mandel, Nicotinamide mononucleotide activation of new DNA-dependent polyadenylic acid synthesizing nuclear enzyme. Biochem Biophys Res Commun, 1963. 11: p. 39-43.
6. Schreiber, V, JC Amé, P Dollé, et al., Poly(ADP-ribose) polymerase-2 (PARP-2) is required for efficient base excision DNA repair in association with PARP-1 and XRCC1. J Biol Chem, 2002. 277(25): p. 23028-36.
7. Curtin, NJ, PARP inhibitors for cancer therapy. Expert Rev Mol Med, 2005. 7(4): p. 1-20.
8. Amé, JC, V Rolli, V Schreiber, et al., PARP-2, A novel mammalian DNA damage-dependent poly(ADP-ribose) polymerase. J Biol Chem, 1999. 274(25): p. 17860-8.
9. Johansson, M, A human poly(ADP-ribose) polymerase gene family (ADPRTL): cDNA cloning of two novel poly(ADP-ribose) polymerase homologues. Genomics, 1999. 57(3): p. 442-5.
10. Fernet, M, V Ponette, E Deniaud-Alexandre, et al., Poly(ADP-ribose) polymerase, a major determinant of early cell response to ionizing radiation. Int J Radiat Biol, 2000. 76(12): p. 1621-9.
11. Shall, S and G de Murcia, Poly(ADP-ribose) polymerase-1: what have we learned from the deficient mouse model? Mutat Res, 2000. 460(1): p. 1-15.
12. Masutani, M, T Nozaki, K Nakamoto, et al., The response of Parp knockout mice against DNA damaging agents. Mutat Res, 2000. 462(2-3): p. 159-66.
13. Ménissier de Murcia, J, M Ricoul, L Tartier, et al., Functional interaction between PARP-1 and PARP-2 in chromosome stability and embryonic development in mouse. EMBO J, 2003. 22(9): p. 2255-63.
14. Yu, SW, H Wang, MF Poitras, et al., Mediation of poly(ADP-ribose) polymerase-1-dependent cell death by apoptosis-inducing factor. Science, 2002. 297(5579): p. 259-63.
15. D'Amours, D, FR Sallmann, VM Dixit, et al., Gain-of-function of poly(ADP-ribose) polymerase-1 upon cleavage by apoptotic proteases: implications for apoptosis. J Cell Sci, 2001. 114(Pt 20) p. 3771-8.
16. Memisoglu, A and L Samson, Base excision repair in yeast and mammals. Mutat Res, 2000. 451(1-2): p. 39-51.
17. Tomoda, T, T Kurashige, T Moriki, et al., Enhanced expression of poly(ADP-ribose)

synthetase gene in malignant lymphoma. Am J Hematol, 1991. 37(4): p. 223-7.
18. Shiobara, M, M Miyazaki, H Ito, et al., Enhanced polyadenosine diphosphate-ribosylation in cirrhotic liver and carcinoma tissues in patients with hepatocellular carcinoma. J Gastroenterol Hepatol, 2001. 16(3): p. 338-44.
19. Fukushima, M, K Kuzuya, K Ota, et al., Poly(ADP-ribose) synthesis in human cervical cancer cell-diagnostic cytological usefulness. Cancer Lett, 1981. 14(3): p. 227-36.
20. Alderson, T, New targets for cancer chemotherapy--poly(ADP-ribosylation) processing and polyisoprene metabolism. Biol Rev Camb Philos Soc, 1990. 65(4): p. 623-41.
21. Wielckens, K, M Garbrecht, M Kittler, et al., ADP-ribosylation of nuclear proteins in normal lymphocytes and in low-grade malignant non-Hodgkin lymphoma cells. Eur J Biochem, 1980. 104(1): p. 279-87.
22. Turner, N, A Tutt, and A Ashworth, Hallmarks of 'BRCAness' in sporadic cancers. Nat Rev Cancer, 2004. 4(10): p. 814-9.
23. Bryant, HE, N Schultz, HD Thomas, et al., Specific killing of BRCA2-deficient tumours with inhibitors of poly(ADP-ribose) polymerase. Nature, 2005. 434(7035): p. 913-7.
24. Farmer, H, N McCabe, CJ Lord, et al., Targeting the DNA repair defect in BRCA mutant cells as a therapeutic strategy. Nature, 2005. 434(7035): p. 917-21.
25. Hay, T and AR Clarke, DNA damage hypersensitivity in cells lacking BRCA2: a review of in vitro and in vivo data. Biochem Soc Trans, 2005. 33(Pt 4): p. 715-7.
26. Ashworth, A, A synthetic lethal therapeutic approach: poly(ADP) ribose polymerase inhibitors for the treatment of cancers deficient in DNA double-strand break repair. J Clin Oncol, 2008. 26(22): p. 3785-90.
27. Fong, PC, DS Boss, TA Yap, et al., Inhibition of poly(ADP-ribose) polymerase in tumors from BRCA mutation carriers. N Engl J Med, 2009. 361(2): p. 123-34.
28. Fong, PC, TA Yap, DS Boss, et al., Poly(ADP)-ribose polymerase inhibition: frequent durable responses in BRCA carrier ovarian cancer correlating with platinum-free interval. J Clin Oncol, 2010. 28(15): p. 2512-9.
29. Audeh, MW, J Carmichael, RT Penson, et al., Oral poly(ADP-ribose) polymerase inhibitor olaparib in patients with BRCA1 or BRCA2 mutations and recurrent ovarian cancer: a proof-of-concept trial. Lancet, 2010. 376(9737): p. 245-51.
30. Tutt, A, M Robson, JE Garber, et al., Oral poly(ADP-ribose) polymerase inhibitor olaparib in patients with BRCA1 or BRCA2 mutations and advanced breast cancer: a proof-of-concept trial. Lancet, 2010. 376(9737): p. 235-44.
31. Dedes, KJ, D Wetterskog, AM Mendes-Pereira, et al., Preclinical evaluation of PARP inhibition as a treatment for endometrioid endometrial carcinomas. J Clin Oncol, 2010. 28:15s (suppl; abstr 5065).
32. Mendes-Pereira, AM, SA Martin, R Brough, et al., Synthetic lethal targeting of PTEN mutant cells with PARP inhibitors. EMBO Mol Med, 2009. 1(6-7): p. 315-22.
33. Shen, Y, Y Feng, B Wang, et al., LT-00673, a highly potent PARPIs, shows antitumor activity against tumor cells with DNA repair deficiencies. AACR 101st Annual Meeting. AACR 101st Annual Meeting, Washington DC Poster # 3514, 2010.
34. Vilar Sanchez, E, A Chow, L Raskin, et al., Preclinical testing of the PARP inhibitor

ABT-888 in microsatellite instable colorectal cancer J Clin Oncol, 2009. 27:15s, (suppl; abstr 11028).
35. Zhao, W, W Duan, ME Leon, et al., Targeting fanconi anemia (FA) repair pathway deficiency for treatment with PARP inhibitors. J Clin Oncol 2010. 28: p. 15s (suppl; abstr TPS168)
36. Gaymes, TJ, S Shall, F Farzaneh, et al., Chromosomal instability syndromes are sensitive to poly ADP-ribose polymerase inhibitors. Haematologica, 2008. 93(12): p. 1886-9.
37. Byers, LA, MB Nilsson, F Masrorpour, et al., Investigation of PARP1 as a therapeutic target in small cell lung cancer. J Clin Oncol, 2012. 30,(suppl; abstr 7096).
38. Brochure, Is, BMN 637 (Version 5.0, 2014). BioMarin Pharmaceutical Inc, Novato, CA.
39. De Bono, JS, LA Mina, M Gonzalez, et al., First-in-human trial of novel oral PARP inhibitor BMN 673 in patients with solid tumors. J Clin Oncol 2013. (suppl; abstr 2580).
40. Belani, CP, CM Kearns, EG Zuhowski, et al., Phase I trial, including pharmacokinetic and pharmacodynamic correlations, of combination paclitaxel and carboplatin in patients with metastatic non-small-cell lung cancer. J Clin Oncol, 1999. 17(2): p. 676-84.
41. Schiller, JH, D Harrington, CP Belani, et al., Comparison of four chemotherapy regimens for advanced non-small-cell lung cancer. N Engl J Med, 2002. 346(2): p. 92-8.
42. Rosell, R, U Gatzemeier, DC Betticher, et al., Phase III randomised trial comparing paclitaxel/carboplatin with paclitaxel/cisplatin in patients with advanced non-small-cell lung cancer: a cooperative multinational trial. Ann Oncol, 2002. 13(10): p. 1539-49.
43. Belani, CP, JS Lee, MA Socinski, et al., Randomized phase III trial comparing cisplatin-etoposide to carboplatin-paclitaxel in advanced or metastatic non-small cell lung cancer. Ann Oncol, 2005. 16(7): p. 1069-75.
44. Kelly, K, J Crowley, PA Bunn, et al., Randomized phase III trial of paclitaxel plus carboplatin versus vinorelbine plus cisplatin in the treatment of patients with advanced non--small-cell lung cancer: a Southwest Oncology Group trial. J Clin Oncol, 2001. 19(13): p. 3210-8.
45. Ozols, RF, BN Bundy, BE Greer, et al., Phase III trial of carboplatin and paclitaxel compared with cisplatin and paclitaxel in patients with optimally resected stage III ovarian cancer: a Gynecologic Oncology Group study. J Clin Oncol, 2003. 21(17): p. 3194-200.
46. Vokes, EE, K Stenson, FR Rosen, et al., Weekly carboplatin and paclitaxel followed by concomitant paclitaxel, fluorouracil, and hydroxyurea chemoradiotherapy: curative and organ-preserving therapy for advanced head and neck cancer. J Clin Oncol, 2003. 21(2): p. 320-6.
47. Keresztes, RS, JL Port, MW Pasmantier, et al., Preoperative chemotherapy for esophageal cancer with paclitaxel and carboplatin: results of a phase II trial. J Thorac Cardiovasc Surg, 2003. 126(5): p. 1603-8.
48. Fountzilas, G, HP Kalofonos, U Dafni, et al., Paclitaxel and epirubicin versus paclitaxel and carboplatin as first-line chemotherapy in patients with advanced breast cancer: a phase III study conducted by the Hellenic Cooperative Oncology Group.

Ann Oncol, 2004. 15(10): p. 1517-26.

49. Vaughn, DJ, J Manola, R Dreicer, et al., Phase II study of paclitaxel plus carboplatin in patients with advanced carcinoma of the urothelium and renal dysfunction (E2896): a trial of the Eastern Cooperative Oncology Group. Cancer, 2002. 95(5): p. 1022-7.
50. Juarez-Salinas, H, JL Sims, and MK Jacobson, Poly(ADP-ribose) levels in carcinogen- treated cells. Nature, 1979. 282(5740): p. 740-1.
51. Pignata, S, G Scambia, D Katsaros, et al., Carboplatin plus paclitaxel once a week versus every 3 weeks in patients with advanced ovarian cancer (MITO-7): a randomised, multicentre, open-label, phase 3 trial. The lancet oncology, 2014. 15(4): p. 396-405.
52. Katsumata, N, M Yasuda, S Isonishi, et al., Long-term results of dose-dense paclitaxel and carboplatin versus conventional paclitaxel and carboplatin for treatment of advanced epithelial ovarian, fallopian tube, or primary peritoneal cancer (JGOG 3016): a randomised, controlled, open-label trial. The lancet oncology, 2013. 14(10): p. 1020-6.
53. Belani, CP, S Ramalingam, MC Perry, et al., Randomized, phase III study of weekly paclitaxel in combination with carboplatin versus standard every-3-weeks administration of carboplatin and paclitaxel for patients with previously untreated advanced non-small- cell lung cancer. J Clin Oncol, 2008. 26(3): p. 468-73.
54. Durkacz, BW, O Omidiji, DA Gray, et al., (ADP-ribose)n participates in DNA excision repair. Nature, 1980. 283(5747): p. 593-6.
55. Rottenberg, S, JE Jaspers, A Kersbergen, et al., High sensitivity of BRCA1-deficient mammary tumors to the PARP inhibitor AZD2281 alone and in combination with platinum drugs. Proceedings of the National Academy of Sciences of the United States of America, 2008. 105(44): p. 17079-84.
56. Donawho, CK, Y Luo, TD Penning, et al., ABT-888, an orally active poly(ADP-ribose) polymerase inhibitor that potentiates DNA-damaging agents in preclinical tumor models. Clin Cancer Res, 2007. 13(9): p. 2728-37.
57. Donawho, CK, Y Luo, TD Penning, et al., ABT-888, an orally active poly(ADP-ribose) polymerase inhibitor that potentiates DNA-damaging agents in preclinical tumor models. Clinical cancer research : an official journal of the American Association for Cancer Research, 2007. 13(9): p. 2728-37.
58. Ivashkevich, A, CE Redon, AJ Nakamura, et al., Use of the gamma-H2AX assay to monitor DNA damage and repair in translational cancer research. Cancer Letters, 2012. 327(1-2): p. 123-33.
59. Lee, SA, C Roques, AC Magwood, et al., Recovery of deficient homologous recombination in Brca2-depleted mouse cells by wild-type Rad51 expression. DNA repair, 2009. 8(2): p. 170-81.
60. Schultz, N, E Lopez, N Saleh-Gohari, et al., Poly(ADP-ribose) polymerase (PARP-1) has a controlling role in homologous recombination. Nucleic Acids Research, 2003. 31(17): p. 4959-64.
61. Kinders, RJ, M Hollingshead, S Khin, et al., Preclinical modeling of a phase 0 clinical trial: qualification of a pharmacodynamic assay of poly (ADP-ribose) polymerase in tumor biopsies of mouse xenografts. Clinical cancer research : an official journal of the American Association for Cancer Research, 2008. 14(21): p. 6877-85.

62. Balmana, J, NM Tung, SJ Isakoff, et al., Phase I trial of olaparib in combination with cisplatin for the treatment of patients with advanced breast, ovarian and other solid tumors. Annals of oncology : official journal of the European Society for Medical Oncology / ESMO, 2014. 25(8): p. 1656-63.
63. Dent, RA, GJ Lindeman, M Clemons, et al., Phase I trial of the oral PARP inhibitor olaparib in combination with paclitaxel for first- or second-line treatment of patients with metastatic triple-negative breast cancer. Breast cancer research : BCR, 2013. 15(5): p. R88.
64. Puhalla, S, L Appleman, J Beumer, et al., Two phase I trials exploring different dosing schedules of carboplatin (C), paclitaxel (P), and the poly-ADP-ribose polymerase (PARP) inhibitor, veliparib (ABT-888) (V) with activity in triple negative breast cancer (TNBC) Cancer Research: December 15, 2012; Volume 72, Issue 24, Supplement 3.
65. Henshaw, J, H Zhou, and A Herriott, Inhibition of PBMC PARP activity with the novel PARP 1/2 inhibitor BMN 673 in patients with advanced solid tumors. Proceedings of the AACR-NCI-EORTC International Conference: AACR. Mol Cancer Ther. 12: Abstract A220. 2013.
66. Kummar, S, R Kinders, ME Gutierrez, et al., Phase 0 clinical trial of the poly (ADP-ribose) polymerase inhibitor ABT-888 in patients with advanced malignancies. Journal of clinical oncology : official journal of the American Society of Clinical Oncology, 2009. 27(16): p. 2705-11.
67. Bunting, SF, E Callen, N Wong, et al., 53BP1 inhibits homologous recombination in Brca1-deficient cells by blocking resection of DNA breaks. Cell, 2010. 141(2): p. 243-54.
68. Bouwman, P, A Aly, JM Escandell, et al., 53BP1 loss rescues BRCA1 deficiency and is associated with triple-negative and BRCA-mutated breast cancers. Nature structural & molecular biology, 2010. 17(6): p. 688-95.
69. Aly, A and S Ganesan, BRCA1, PARP, and 53BP1: conditional synthetic lethality and synthetic viability. Journal of molecular cell biology, 2011. 3(1): p. 66-74.
70. Jaspers, JE, A Kersbergen, U Boon, et al., Loss of 53BP1 causes PARP inhibitor resistance in Brca1-mutated mouse mammary tumors. Cancer discovery, 2013. 3(1): p. 68-81.
71. Callen, E, M Di Virgilio, MJ Kruhlak, et al., 53BP1 mediates productive and mutagenic DNA repair through distinct phosphoprotein interactions. Cell, 2013. 153(6): p. 1266-80.
72. Zimmermann, M, F Lottersberger, SB Buonomo, et al., 53BP1 regulates DSB repair using Rif1 to control 5' end resection. Science, 2013. 339(6120): p. 700-4.
73. Edwards, SL, R Brough, CJ Lord, et al., Resistance to therapy caused by intragenic deletion in BRCA2. Nature, 2008. 451(7182): p. 1111-5.
74. Sakai, W, EM Swisher, BY Karlan, et al., Secondary mutations as a mechanism of cisplatin resistance in BRCA2-mutated cancers. Nature, 2008. 451(7182): p. 1116-20.
75. Gibbons, J, MJ Egorin, RK Ramanathan, et al., Phase I and pharmacokinetic study of imatinib mesylate in patients with advanced malignancies and varying degrees of renal dysfunction: a study by the National Cancer Institute Organ Dysfunction Working Group. J Clin Oncol, 2008. 26(4): p. 570-6.
76. Lu, D, A Joshi, B Wang, et al., An integrated multiple-analyte pharmacokinetic model

to characterize trastuzumab emtansine (T-DM1) clearance pathways and to evaluate reduced pharmacokinetic sampling in patients with HER2-positive metastatic breast cancer. Clinical Pharmacokinetics, 2013. 52(8): p. 657-72.

77. Mukhopadhyay, A, A Elattar, A Cerbinskaite, et al., Development of a functional assay for homologous recombination status in primary cultures of epithelial ovarian tumor and correlation with sensitivity to poly(ADP-ribose) polymerase inhibitors. Clinical cancer research : an official journal of the American Association for Cancer Research, 2010. 16(8): p. 2344-51.
78. Wang, LH, TD Pfister, RE Parchment, et al., Monitoring drug-induced gammaH2AX as a pharmacodynamic biomarker in individual circulating tumor cells. Clinical cancer research : an official journal of the American Association for Cancer Research, 2010. 16(3): p. 1073-84.
79. Eisenhauer, EA, P Therasse, J Bogaerts, et al., New response evaluation criteria in solid tumours: revised RECIST guideline (version 1.1). Eur J Cancer, 2009. 45(2): p. 228-47.
80. Rustin, GJ, M Quinn, T Thigpen, et al., Re: New guidelines to evaluate the response to treatment in solid tumors (ovarian cancer). J Natl Cancer Inst, 2004. 96(6): p. 487-8.
81. Bubley, GJ, M Carducci, W Dahut, et al., Eligibility and response guidelines for phase II clinical trials in androgen-independent prostate cancer: recommendations from the Prostate-Specific Antigen Working Group. J Clin Oncol, 1999. 17(11): p. 3461-7.
82. Scher, HI, S Halabi, I Tannock, et al., Design and end points of clinical trials for patients with progressive prostate cancer and castrate levels of testosterone: recommendations of the Prostate Cancer Clinical Trials Working Group. J Clin Oncol, 2008. 26(7): p. 1148-59.
83. Vergote, I, GJ Rustin, EA Eisenhauer, et al., Re: new guidelines to evaluate the response to treatment in solid tumors [ovarian cancer]. Gynecologic Cancer Intergroup. J Natl Cancer Inst, 2000. 92(18): p. 1534-5.

APPENDIX A PERFORMANCE STATUS CRITERIA

**ECOG Performance Status Scale**		**Karnofsky Performance Scale**	
Grade	Descriptions	Percent	Description
0	Normal activity. Fully active, able to carry on all pre-disease performance without restriction.	100	Normal, no complaints, no evidence of disease.
		90	Able to carry on normal activity; minor signs or symptoms of disease.
1	Symptoms, but ambulatory. Restricted in physically strenuous activity, but ambulatory and able to carry out work of a light or sedentary nature (*e.g.*, light housework, office work).	80	Normal activity with effort; some signs or symptoms of disease.
		70	Cares for self, unable to carry on normal activity or to do active work.
2	In bed <50% of the time. Ambulatory and capable of all self-care, but unable to carry out any work activities. Up and about more than 50% of waking hours.	60	Requires occasional assistance, but is able to care for most of his/her needs.
		50	Requires considerable assistance and frequent medical care.
3	In bed >50% of the time. Capable of only limited self-care, confined to bed or chair more than 50% of waking hours.	40	Disabled, requires special care and assistance.
		30	Severely disabled, hospitalization indicated. Death not imminent.
4	100% bedridden. Completely disabled. Cannot carry on any self-care. Totally confined to bed or chair.	20	Very sick, hospitalization indicated. Death not imminent.
		10	Moribund, fatal processes progressing rapidly.
5	Dead.	0	Dead.

APPENDIX B Talazoparib (BMN 673) MEDICATION DIARY

NCI 9782 - MEDICATION DIARY for Talazoparib (BMN 673)

Cycle 1- Dose Escalation and Dose Expansion

Patient Initials:____________

This is a medication diary on which you are to record the Talazoparib (BMN 673) medication you take. Enter the time when the medication is taken, as well as the dose of medication taken. You will begin taking the Talazoparib (BMN 673) on Study Day 1 and continuing through Day 3 or Day 7, depending on the dosing schedule you are assigned to.

______Schedule A –Take Talazoparib (BMN 673) for the first 7 days of each 21-day cycle.

______Schedule B –Take Talazoparib (BMN 673) for the first 3 days of each 21-day cycle.

- **For your Talazoparib (BMN 673) dose of___mcg once per day, you need to take____of the______________mcg tablets and________________________of the_____mcg tablets- once daily as instructed with a glass of water.**
- **Tablets can be taken without regard to meals.**
- **If the medication is not taken, please specify the reason. If you vomit after taking the dose, please indicate on study diary, but do not repeat dose.**
- **You are to return this diary and your medication bottle to your Research Nurse. Please sign and date this medication diary once it is completed (on the bottom of the next page).**
- **Daily dose may be taken up to 12 hours past the scheduled 24 hour time interval. Doses not taken before the end of this 12 hour extension period will be considered missed. Missed doses should NOT be made up.**
- **Daily dose should be taken in the morning.**

*** On Cycle 1, Day 1-your study medication (Talazoparib (BMN 673)) will be dispensed to you and your nurse will instruct you when to take your study medication. On Cycle 1, Day 3 (or Day 7 depending on the last day of your assigned dosing schedule)- bring study medication with you to your research visit and don't take your Talazoparib (BMN 673) dose until instructed by the nurse.**

Schedule A- Talazoparib (BMN 673) for first 7 days of each 21-day cycle. **Cycle 1**

Study Day	Date MM/DAY/YEAR		Time Taken	Dose (mcg)	Comments (Side effects, complaints, other
1*		Talazoparib (BMN 673) Dose	Time:______	________mcg	
2		Talazoparib (BMN 673) Dose	Time:______	________mcg	
3		Talazoparib (BMN 673) Dose	Time:______	________mcg	
4		Talazoparib BMN 673 Dose	Time:______	________mcg	
5		Talazoparib BMN 673 Dose	Time:______	________mcg	
6		Talazoparib BMN 673 Dose	Time:______	________mcg	
7*		Talazoparib BMN 673 Dose	Time:______	________mcg	

Schedule B- Talazoparib (BMN 673) for first 3 days of each 21-day cycle. **Cycle 1**

Study Day	Date MM/DAY/YEAR		Time Taken	Dose (mcg)	Comments (Side effects, complaints, other
1*		Talazoparib (BMN 673) Dose	Time:______	________mcg	
2		Talazoparib (BMN 673) Dose	Time:______	________mcg	
3*		Talazoparib (BMN 673) Dose	Time:______	________mcg	

______________________________ ______________
Patient Signature **Date**

NCI 9782 - MEDICATION DIARY for Talazoparib (BMN 673)

Cycles 2 and beyond- Dose Escalation and Dose Expansion

Patient Initials:____________ Cycle:_______________

This is a medication diary on which you are to record the Talazoparib (BMN 673) medication you take. Enter the time when the medication is taken, as well as the dose of medication taken. You will begin taking the Talazoparib (BMN 673) on Cycle Day 1 and continuing through Day 3 or Day 7, depending on the dosing schedule you are assigned to.

______Schedule A –Take Talazoparib (BMN 673) for the first 7 days of each 21-day cycle.

______Schedule B –Take Talazoparib (BMN 673) for the first 3 days of each 21-day cycle.

- **For your Talazoparib (BMN 673) dose of____mcg once per day, you need to take_____of the_______________mcg tablets and_________________________of the_____mcg tablets- once daily as instructed with a glass of water.**
- **Tablets can be taken without regard to meals.**
- **If the medication is not taken, please specify the reason. If you vomit after taking the dose, please indicate on study diary, but do not repeat dose.**
- **You are to return this diary and your medication bottle to your Research Nurse. Please sign and date this medication diary once it is completed (on the bottom of the next page).**
- **Daily dose may be taken up to 12 hours past the scheduled 24 hour time interval. Doses not taken before the end of this 12 hour extension period will be considered missed. Missed doses should NOT be made up.**
- **Daily dose should be taken in the morning.**

*** On Day 1 of each cycle, your study medication (Talazoparib (BMN 673)) will be dispensed to you. Your nurse will instruct you when to take your study medication on the first day. Research blood samples also may be drawn on Day 1.**

Schedule A-Talazoparib (BMN 673) for first 7 days of each 21-day cycle.
Cycle___________________

Study Day	Date MM/DAY/YEAR		Time Taken	Dose (mcg)	Comments (Side effects, complaints, other
1*		Talazoparib (BMN 673) Dose	Time:______	________mcg	
2		Talazoparib (BMN 673) Dose	Time:______	________mcg	
3		Talazoparib (BMN 673) Dose	Time:______	________mcg	
4		Talazoparib (BMN 673) Dose	Time:______	________mcg	
5		Talazoparib (BMN 673) Dose	Time:______	________mcg	
6		Talazoparib (BMN 673) Dose	Time:______	________mcg	
7		Talazoparib (BMN 673) Dose	Time:______	________mcg	

Schedule B- Talazoparib (BMN 673) for first 3 days of each 21-day cycle.
Cycle___________________

Study Day	Date MM/DAY/YEAR		Time Taken	Dose (mcg)	Comments (Side effects, complaints, other
1*		Talazoparib (BMN 673) Dose	Time:______	________mcg	
2		Talazoparib (BMN 673) Dose	Time:______	________mcg	
3		Talazoparib (BMN 673) Dose	Time:______	________mcg	

______________________________ ______________
Patient Signature **Date**

NCI 9782 - MEDICATION DIARY for Talazoparib (BMN 673)

Talazoparib (BMN 673) continuous dosing- Dose Escalation and Dose Expansion

Patient Initials:____________ Cycle:______________

This is a medication diary on which you are to record the Talazoparib (BMN 673) medication you take. Enter the time when the medication is taken, as well as the dose of medication taken. You will begin taking the Talazoparib (BMN 673) on Cycle_____Day 1 and continuing through Day 21 of each 21-day cycle.

- **For your Talazoparib (BMN 673) dose of____mcg once per day, you need to take_____of the______________mcg tablets and_________________________of the______mcg tablets- once daily as instructed with a glass of water.**
- **Tablets can be taken without regard to meals.**
- **If the medication is not taken, please specify the reason. If you vomit after taking the dose, please indicate on study diary, but do not repeat dose.**
- **You are to return this diary and your medication bottle to your Research Nurse. Please sign and date this medication diary once it is completed (on the bottom of the next page).**
- **Daily dose may be taken up to 12 hours past the scheduled 24 hour time interval. Doses not taken before the end of this 12 hour extension period will be considered missed. Missed doses should NOT be made up.**
- **Daily dose should be taken in the morning.**

*** On Day 1 of each cycle, your study medication (Talazoparib (BMN 673)) will be dispensed to you. Your nurse will instruct you when to take your study medication on the first day. Research blood samples also may be drawn on Day 1.**

Talazoparib (BMN 673) for 21 days of each 21-day cycle. **Cycle_________________**

**Study Day**	**Date MM/DAY/YEAR**		**Time Taken**	**Dose (mcg)**	**Comments** (Side effects, complaints, other medication)
1*		Talazoparib (BMN 673) Dose	Time:______	________mcg	
2		Talazoparib (BMN 673) Dose	Time:______	________mcg	
3		Talazoparib (BMN 673) Dose	Time:______	________mcg	
4		Talazoparib (BMN 673) Dose	Time:______	________mcg	
5		Talazoparib (BMN 673) Dose	Time:______	________mcg	
6		Talazoparib (BMN 673) Dose	Time:______	________mcg	
7		Talazoparib (BMN 673) Dose	Time:______	________mcg	
8		Talazoparib (BMN 673) Dose	Time:______	________mcg	
9		Talazoparib (BMN 673) Dose	Time:______	________mcg	
10		Talazoparib (BMN 673) Dose	Time:______	________mcg	
11		Talazoparib (BMN 673) Dose	Time:______	________mcg	
12		Talazoparib (BMN 673) Dose	Time:______	________mcg	
13		Talazoparib (BMN 673) Dose	Time:______	________mcg	
14		Talazoparib (BMN 673) Dose	Time:______	________mcg	
15		Talazoparib (BMN 673) Dose	Time:______	________mcg	

16		Talazoparib (BMN 673) Dose	Time:_______	________mcg	
17		Talazoparib (BMN 673) Dose	Time:_______	________mcg	
18		Talazoparib (BMN 673) Dose	Time:_______	________mcg	
19		Talazoparib (BMN 673) Dose	Time:_______	________mcg	
20		Talazoparib (BMN 673) Dose	Time:_______	________mcg	
21		Talazoparib (BMN 673) Dose	Time:_______	________mcg	

______________________________ ______________

Patient Signature **Date**

NCI 9782 - MEDICATION DIARY for Talazoparib (BMN 673)

Talazoparib (BMN 673) continuous dosing- Dose Escalation and Dose Expansion
42-day cycle

Patient Initials:___ ___ ___ Cycle:______________

This is a medication diary on which you are to record the Talazoparib (BMN 673) medication you take. Enter the time when the medication is taken, as well as the dose of medication taken. You will begin taking the Talazoparib (BMN 673) on Cycle ____ Day 1 and continuing through Day 42 of each 42-day cycle.

- **For your Talazoparib (BMN 673) dose of ______mcg once per day, you need to take _____ of the _____ mcg tablets and _____ of the _____ mcg tablets- once daily as instructed with a glass of water.**
- **Tablets can be taken without regard to meals.**
- **If the medication is not taken, please specify the reason. If you vomit after taking the dose, please indicate on study diary, but do not repeat dose.**
- **You are to return this diary and your medication bottle to your Research Nurse. Please sign and date this medication diary once it is completed (on the bottom of the next page).**
- **Daily dose may be taken up to 12 hours past the scheduled 24 hour time interval. Doses not taken before the end of this 12 hour extension period will be considered missed. Missed doses should NOT be made up.**
- **Daily dose should be taken in the morning.**

*** On Day 1 of each cycle, your study medication (Talazoparib (BMN 673)) will be dispensed to you. Your nurse will instruct you when to take your study medication on the first day.**

Talazoparib (BMN 673) for 42 days of each 42-day cycle.   Cycle__________________

Study Day	Date MM/DAY/YEAR		Time Taken	Dose (mcg)	**Comments** (Side effects, complaints, other medication)
1*		Talazoparib (BMN 673) Dose	Time:______	________mcg	
2		Talazoparib (BMN 673) Dose	Time:______	________mcg	
3		Talazoparib (BMN 673) Dose	Time:______	________mcg	
4		Talazoparib (BMN 673) Dose	Time:______	________mcg	
5		Talazoparib (BMN 673) Dose	Time:______	________mcg	
6		Talazoparib (BMN 673) Dose	Time:______	________mcg	
7		Talazoparib (BMN 673) Dose	Time:______	________mcg	
8		Talazoparib (BMN 673) Dose	Time:______	________mcg	
9		Talazoparib (BMN 673) Dose	Time:______	________mcg	
10		Talazoparib (BMN 673) Dose	Time:______	________mcg	
11		Talazoparib (BMN 673) Dose	Time:______	________mcg	
12		Talazoparib (BMN 673) Dose	Time:______	________mcg	
13		Talazoparib (BMN 673) Dose	Time:______	________mcg	
14		Talazoparib (BMN 673) Dose	Time:______	________mcg	

15		Talazoparib (BMN 673) Dose	Time:______	________mcg	
16		Talazoparib (BMN 673) Dose	Time:______	________mcg	
17		Talazoparib (BMN 673) Dose	Time:______	________mcg	
18		Talazoparib (BMN 673) Dose	Time:______	________mcg	
19		Talazoparib (BMN 673) Dose	Time:______	________mcg	
20		Talazoparib (BMN 673) Dose	Time:______	________mcg	
21		Talazoparib (BMN 673) Dose	Time:______	________mcg	
22		Talazoparib (BMN 673) Dose	Time:______	________mcg	
23		Talazoparib (BMN 673) Dose	Time:______	________mcg	
24		Talazoparib (BMN 673) Dose	Time:______	________mcg	
25		Talazoparib (BMN 673) Dose	Time:______	________mcg	
26		Talazoparib (BMN 673) Dose	Time:______	________mcg	
27		Talazoparib (BMN 673) Dose	Time:______	________mcg	
28		Talazoparib (BMN 673) Dose	Time:______	________mcg	
29		Talazoparib (BMN 673) Dose	Time:______	________mcg	
30		Talazoparib (BMN 673) Dose	Time:______	________mcg	

31		Talazoparib (BMN 673) Dose	Time:______	________mcg	
32		Talazoparib (BMN 673) Dose	Time:______	________mcg	
33		Talazoparib (BMN 673) Dose	Time:______	________mcg	
34		Talazoparib (BMN 673) Dose	Time:______	________mcg	
35		Talazoparib (BMN 673) Dose	Time:______	________mcg	
36		Talazoparib (BMN 673) Dose	Time:______	________mcg	
37		Talazoparib (BMN 673) Dose	Time:______	________mcg	
38		Talazoparib (BMN 673) Dose	Time:______	________mcg	
39		Talazoparib (BMN 673) Dose	Time:______	________mcg	
40		Talazoparib (BMN 673) Dose	Time:______	________mcg	
41		Talazoparib (BMN 673) Dose	Time:______	________mcg	
42		Talazoparib (BMN 673) Dose	Time:______	________mcg	

______________________________ ______________
Patient Signature **Date**

APPENDIX C: LIST OF CONTRAINDICATED MEDICATIONS*

**CYP2C8 strong inhibitors**	gemfibrozil
**CYP2C8 moderate inhibitors**	trimethoprim, glitazones, montelukast, quercetin
**CYP2C8 inducers**	rifampin
**CYP3A4 strong inhibitors**	indinavir, nelvinavir, ritonavir, clarithromycin, itraconazole, ketoconazole, nefazodone, saquinavir, suboxone, telithromycin
**CYP3A4 moderate inhibitors**	aprepitant, erythromycin, fluconazole, grapefruit juice, verapamil, diltiazem
**CYP3A4 inducers**	efavirenz, nevirapine, barbiturates, carbamazepine, glucocorticoids, modafinil, oxcarbazepine, phenobarbital, phenytoin, pioglitazone, rifabutin, rifampin, St. John's wort, troglitazone
**CYP3A4 sensitive substrates**	clarithromycin, erythromycin, quinidine, alprazolam, diazepam, midazolam, triazolam, cyclosporine, tacrolimus, indinavir, nelfinavir, ritonavir, saquinavir, cisapride, astemizole, chlorpheniramine, terfenadine, amlodipine, diltiazem, felodipine, lercanidipine, nifedipine, nisoldipine, nitrendipine, verapamil, atorvastatin, cerivastatin, lovastatin, simvastatin, estradiol, hydrocortisone, progesterone, testosterone, alfentanil, aprepitant, aripiprazole, boceprevir, buspirone, carbamazepine, cafergot, caffeine -> TMU, cilostazol, cocaine, codeine-N-demethylation, dapsone, dexamethasone, dextromethorphan, docetaxel, domperidone, eplerenone, fentanyl, finasteride, gleevec, haloperidol, irinotecan, LAAM, lidocaine, methadone, nateglinide, nevirapine, ondansteron, primozide, propranolol, quetiapine, quinine, risperidone, romidepsin, salmeterol, sildenafil, sirolimus, sorafenib, sunitinib, tamoxifen, taxol, telaprevir, terfenadine, torisel, trazodone, vemurafenib, vincristine, zaleplon, ziprasidone, zolpidem

* Because the lists of these agents are constantly changing, it is important to regularly consult a frequently-updated medical reference for a list of drugs to avoid.

APPENDIX D: INFORMATION ON POSSIBLE DRUG INTERACTIONS

Information on Possible Interactions with Other Agents for Patients and Their Caregivers and Non-Study Healthcare Team

The patient_____________________________is enrolled on a clinical trial using the experimental agent Talazoparib (BMN 673) along with the FDA approved medications **paclitaxel** and **carboplatin.** *This clinical trial is sponsored by the National Cancer Institute. This form is addressed to the patient, but includes important information for others who care for this patient.*

Paclitaxel interacts with many drugs that are processed by your liver. Because of this, it is very important to tell your study doctors about all of your medicine before you start this study. It is also very important to tell them if you stop taking any regular medicine, or if you start taking a new medicine while you take part in this study. When you talk about your medicine with your study doctor, include medicine you buy without a prescription at the drug store (over-the-counter remedy), or herbal supplements such as St. John's wort.

Many health care providers can write prescriptions. You must also tell your other providers (doctors, physicians' assistants or nurse practitioners) that you are taking part in a clinical trial. **Bring this paper with you and keep the attached information card in your wallet**. These are the things that you and they need to know:

Paclitaxel interacts with two specific enzymes in your liver.

- These two enzymes are called CYP3A4 and CYP2C8
- Paclitaxel must be used very cautiously if given with any other medications whose metabolism involves CYP3A4 or CYP2C8
  - Any medication that increases CYP3A4 or CYP2C8's activity (called inducers) could result in high levels of the active drug, increasing the chance of harmful side effects
  - Any medication that decreases CYP3A4 or CYP2C8's activity (called inhibitors) may reduce the effectiveness of the drug
  - Finally other medications that use the same enzymes for their own metabolism must also be used with caution (these are called substrates)
- Before you start the study, your study doctor will work with your regular provider to switch any medicines that are in the following categories to an alternative as the following are **PROHIBITED:**
  - **Moderate and Strong inducers of CYP2C8**
  - **Inducers of CYP2C8**
  - **Moderate and Strong inducers of CYP3A4**
  - **Inducers of CYP3A4**
  - **Sensitive substrates of CYP3A4**
- Before you start the study, your study doctor will work with your regular provider to evaluate the need for the following medications. If warranted, these may continue to be used with caution while you are on trial. This is at your study doctor's discretion as these are to be **used with CAUTION:**

  - **Sensitive substrates of CYP2C8**
  - **Weak inhibitors of CYP2C8**
  - **Non-sensitive substrates of CYP3A4**
  - **Weak inhibitors of CYP3A4**
- Your prescribers should check a frequently updated medical reference or call your study doctor before prescribing any new medicine or discontinuing any medicine. (per the above)

Other medications that may be **PROHIBITED** while you are on this clinical trial **(do not start taking these without first consulting your study physician)**

- Anticoagulants or antiplatelet agents including warfarin, low molecular weight heparin, factor Xa inhibitors, aspirin and NSAIDs, unless it is for an urgent or life-threatening medical event

- Caution: NSAIDS which may be purchased over the counter include ibuprofen, naproxen and aspirin. Check all labels carefully.

General guidelines

- Please be very careful! Over-the-counter drugs have a brand name on the label—it's usually big and catches your eye. They also have a generic name—it's usually small and located above or below the brand name, and printed in the ingredient list. Find the generic name and determine, with the pharmacist's help, whether there could be an adverse interaction (based upon the information provided above)
- You should check with your doctor or pharmacist FIRST before using an over-the-counter medicine or herbal supplement.
- Be careful:
  - If you take acetaminophen regularly: You should not take more than 4 grams a day if you are an adult or 2.4 grams a day if you are older than 65 years of age. Read labels carefully! Acetaminophen is an ingredient in many medicines for pain, flu, and cold.
  - If you drink grapefruit juice or eat grapefruit: Avoid these until the study is over and discuss with your physician
  - If you take herbal medicine regularly: You should not take St. John's wort while you are receiving paclitaxel

Your study doctor's name is ____

For questions please call the Study Nurse at 608-263-6222.

INFORMATION ON POSSIBLE DRUG INTERACTIONS

You are enrolled on a clinical trial using the experimental agent Talazoparib (BMN 673) along with paclitaxel and carboplatin**.** This clinical trial is sponsored by the NCI. *Paclitaxel* interacts with drugs that are processed by your liver. Because of this, it is very important to:

- Tell your doctors if you stop taking regular medicine or if you start taking a new medicine.
- Tell all of your providers (doctor, physicians' assistant, nurse practitioner, pharmacist) that you are taking part in a clinical trial.
- Check with your doctor or pharmacist whenever you need to use an over-the-counter medicine or herbal supplement.

Paclitaxel interacts with 2 specific liver enzymes called **CYP3A4 and CYP2C8;** caution must be exercised before taking other medications. Before you start the study, your study doctor will work with your regular provider to switch any medicines that are considered "strong inducers/inhibitors or substrates of **CYP3A4 and CYP2C8**."

- Before prescribing new medicines, your regular prescribers should check a frequently-updated drug resource for a list of drugs to avoid, or contact your study doctor.
- **Subjects must avoid anticoagulants and antiplatelet agents including (but not limited to) warfarin, heparin, aspirin and NSAIDS. Physician name: ______________________________**
- CONTACT UW Study RN at 608-263-6222 with questions
